# Photocatalytic sequential C–H functionalization expediting acetoxymalonylation of imidazo heterocycles

**DOI:** 10.3762/bjoc.19.48

**Published:** 2023-05-12

**Authors:** Deepak Singh, Shyamal Pramanik, Soumitra Maity

**Affiliations:** 1 Department of Chemistry and Chemical Biology, Indian Institute of Technology (ISM) Dhanbad, JH 826004, India

**Keywords:** C–H functionalization, imidazo heterocycles, photoredox, regioselective, relay catalysis

## Abstract

The importance of functionalized imidazo heterocycles has often been featured in several impactful research both from academia and industry. Herein, we report a direct C-3 acetoxymalonylation of imidazo heterocycles using relay C–H functionalization enabled by organophotocatalysis starring zinc acetate in the triple role of an activator, ion scavenger as well as an acetylating reagent. The mechanistic investigation revealed a sequential sp^2^ and sp^3^ C–H activation, followed by functionalization driven by zinc acetate coupled with the photocatalyst PTH. A variety of imidazo[1,2-*a*]pyridines and related heterocycles were explored as substrates along with several active methylene reagents, all generating the products with excellent yields and regioselectivity, thus confirming excellent functional group tolerability.

## Introduction

Among all N-fused heterocycles, imidazo[1,2-*a*]pyridines (IPs) are the prevalent moieties in several bioactive pharmaceuticals and natural products [1–4]. Moreover, due to their susceptibility towards 'exited-state intramolecular proton transfer' phenomena, IPs are also effective in optoelectronics and materials sciences [5–6]. C-3-functionalized imidazo[1,2-*a*]pyridines are particularly familiar due to their biological and medicinal attributes [7–11]. Not surprisingly, the C-3 functionalization of IPs is a continuing interest of research in the synthetic community [12–16].

**Scheme 1:**
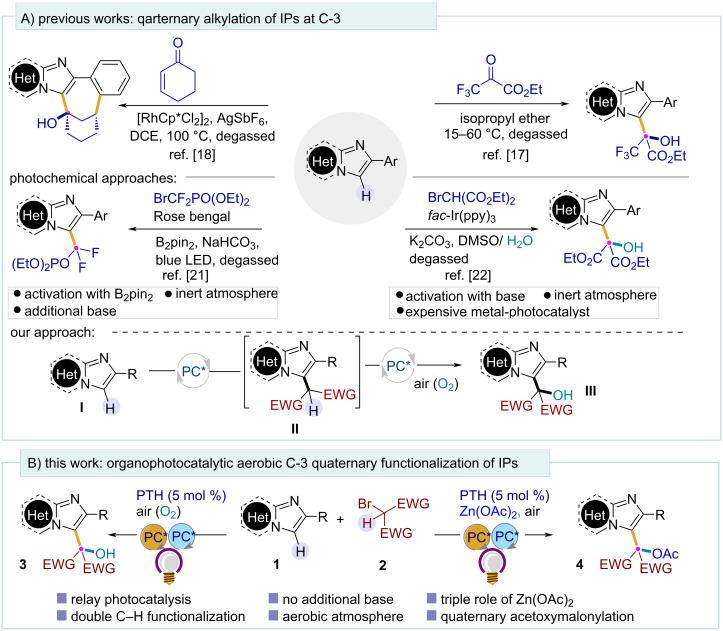
Strategies of C-3 functionalizations of IPs and present work.

Despite many successful strategies in this field, the regioselective C–H functionalization is still challenging for chemists to combine a C(sp^3^) carbon of incoming functionalities and C(sp^2^) carbon of the IP core. The direct C-3 alkylation of imidazopyridines using active malonates and related moieties has been achieved by different routes [17–20]. However, these reactions rely either on harsh reaction conditions or require the preactivation of substrates, which limits their synthetic efficiency. A photocatalytic quaternary C-3 alkylation has also been reported recently (Scheme 1A) [21–22]. During the course of our study, the Wu group reported a solvent-controlled chemodivergent formation of C-3 ethoxycarbonylmethylated and hydroxyalkylated IPs under visible light using water or alcohol as the source of the oxygenated group under degassed conditions [22]. However, all these photochemical methods require the usage of a substantial amount of base, the preactivation with a boron complex (B_2_pin_2_), and using an expensive metal-based photocatalyst [*fac*-Ir(ppy)_3_] under inert atmosphere. We have recently demonstrated that aerial oxygen could be captured by alkyl radicals to install a keto-functionality onto alkenes in an organophotocatalytic way [23]. We aimed to extend this aerobic oxygenation approach to imidazo heterocycles **II** to install the hydroxymalonate unit onto **I** through sequential photoredox C–H functionalization.

Till date, there is no report of the direct incorporation of a quaternary hydroxyalkyl, specifically a hydroxymalonyl group at the C-3 position of IPs using air as the sole oxygen source.

Keeping in mind the progress in photochemical relay catalysis [24] and the attention paid to photocatalytic carbon-bond functionalization in the past several years [25], here we developed an organophotoredox-catalyzed C–H functionalization of imidazo[1,2-*a*]pyridines and related heterocycles with active bromomethylenes under mild conditions (Scheme 1B). Importantly, using simple Zn(OAc)_2_ as the additive, the first photocatalytic direct acetoxymalonylation of imidazo heterocycles was developed under aerobic conditions. Here, the additive Zn(OAc)_2_ plays a crucial triple role as activator of IPs, halide scavenger, and acetylating agent.

## Results and Discussion

### Optimization

In the quest for the optimal reaction conditions, we started our investigations with 2-phenylimidazo[1,2-*a*]pyridine (**1a**) and diethyl bromomalonate (**2a**) as model substrates. Initially, the reaction was carried out between **1a** and **2a** in dry CH_3_CN as solvent under N_2_ atmosphere using 4CzIPN as the photocatalyst. Irradiating the reaction mixture for 10 h under blue LEDs (450 nm) led to the isolation of products **5** (54%) and **6** (28%) (Table 1, entry 1). However, the same reaction, under aerobic conditions, delivered compounds **3a** (47%) and **6** (22%) (Table 1, entry 2). Keeping in mind the ability of Zn(OAc)_2_ as a bromide ion scavenger [26], we used Zn(OAc)_2_ (2 equiv) as an additive to prevent the formation of the bromo product **6**. While the additive successfully prevented the formation of compound **6**, we were delighted to isolate the unexpected acetylated product **4a** with a promising yield of 38% (Table 1, entry 3), reflecting the ability of Zn(OAc)_2_ to act as an acetylating agent. While screening other organophotocatalysts, we detected no desired product **4a** (Table 1, entries 4–6) [27], except for photocatalyst 10-phenylphenothiazine (PTH) under violet LEDs which uplifted the yield up to 52% (Table 1, entry 7). Now with the optimal catalyst in hand, we screened some common solvents, out of which 1,2-DCE positively impacted the yield (Table 1, entries 8–11). However, the best result was obtained when 3.0 equiv of Zn(OAc)_2_ was used as an additive (Table 1, entry 12). To check the viability of other acetylating agents, Zn(OAc)_2_ was replaced with AcOH, generating the desired product in a comparatively lower yield (Table 1, entry 13). Finally, control experiments without a catalyst (Table 1, entry 14), light (entry 15) or acetylation agent (entry 16) failed to provide the desired product **4a**, displaying the necessity of each component for developing the reaction.

**Table 1:** Reaction optimization.^a^

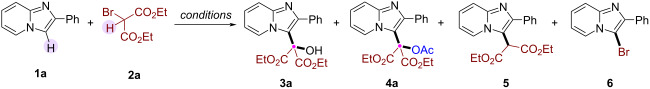				

Entry	Catalyst	Solvent	Additive	Yield (%)^b^ **3a**:**4a**:**5**:**6**

1^c^	4-CzIPN	CH_3_CN	–	0:0:54:28
2	4-CzIPN	CH_3_CN	–	47:0:0:22
3	4-CzIPN	CH_3_CN	Zn(OAc)_2_	0:38:0:0
4	Rose Bengal	CH_3_CN	Zn(OAc)_2_	–
5	eosin-Y	CH_3_CN	Zn(OAc)_2_	–
6	rhodamine-B	CH_3_CN	Zn(OAc)_2_	–
7^d^	PTH	CH_3_CN	Zn(OAc)_2_	0:52:0:0
8	PTH	1,4-dioxane	Zn(OAc)_2_	0:34:0:0
9	PTH	DMF	Zn(OAc)_2_	0:25:0:0
10	PTH	toluene	Zn(OAc)_2_	0:18:0:0
11	PTH	1,2-DCE	Zn(OAc)_2_	0:70:0:0
**12** **^e^**	**PTH**	**1,2-DCE**	**Zn(OAc)** **_2_**	**0:94:0:0**
13	PTH	1,2-DCE	AcOH	0:64:0:0
14	–	1,2-DCE	Zn(OAc)_2_	–
15^f^	PTH	1,2-DCE	Zn(OAc)_2_	–
16	PTH	1,2-DCE	–	57:0:0:24

^a^Reaction conditions: **1a** (0.2 mmol), **2a** (0.4 mmol), catalyst (5 mol %), additive (0.4 mmol) in dry solvent (2 mL) under aerobic conditions, irradiation with 12 W blue LEDs for 10 h. ^b^Isolated yield. ^c^Under N_2_ atmosphere. ^d^Irradiation with violet LEDs (λ_max_ = 390 nm). ^e^3.0 equiv of zinc acetate used. ^f^In the dark, without light source.

### Substrate scope

**Scheme 2:**
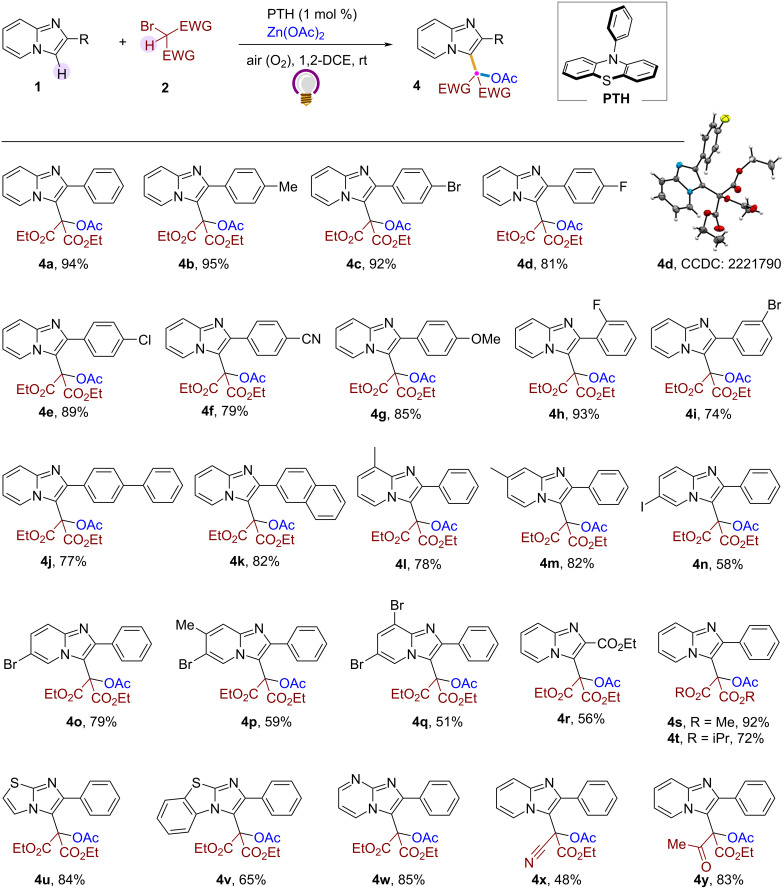
Substrate scope. Conditions: unless otherwise noted, all reactions were carried out with **1** (0.2 mmol), **2** (0.4 mmol), PTH (5 mol %), Zn(OAc)_2_ (0.6 mmol), dry 1,2-DCE (2 mL), irradiation with LEDs (λ_max_ = 390 nm), under air for 10 h.

With suitable reaction conditions (Table 1, entry 12), we systematically investigated the scope of this acetoxymalonylation strategy with substrate **2a** (Scheme 2). Several imidazo[1,2-*a*]pyridines with diverse aryl substituents in the C-2 position were acetoxymalonylated, providing the desired products **4a**–**k** regioselectively in good to excellent yields. Reflection of electronic properties was shown by the substituents attached to the aryl ring – as electron-releasing groups (Me, OMe) showed little more reactivity than electron-withdrawing groups (CN) at the same position (**4b, 4f**, and **4g**). Halogen-substituted IPs also followed the general reactivity trend of the respective halogens (**4c**–**e**). Excellent reactivity was found for *o*-F and *m*-Br-substituted IPs (**4h** and **4i**). Similarly, IPs associated with biphenyl and naphthyl groups in the C-2 position were also suitable substrates giving the corresponding products **4j** and **4k** in 77% and 82% yield, respectively. However, the yield of the products varied when different groups with diverse electronic properties were present in the pyridine ring of the IP moieties (**4l**–**q**). With substrates having a methyl substitution at C-7 and C-8 of the pyridine ring, the yields and regioselectivity were still excellent (**4l** and **4m**), but reduced significantly upon introducing a halogen group onto the pyridine ring. Except for the 6-bromo-substituted compound (**4o**), all other substrates having a halogen substituent in the pyridine ring showed reduced yields (**4n**, and **4p**,**q**). The number of substituents also seemed to negatively affect the yield, as observed for products **4p** and **4q**, featuring two substituents each on the pyridine ring. Moreover, IPs with a non-aromatic C-2 substituent like an ester group were also included (**4r**). We also explored bromo analogues of other active methylenes such as ethyl cyanoacetate, ethyl acetoacetate, dimethyl, and diisopropyl malonates, as extension of diethyl malonate (**4s**,**t** and **4x**,**y)**. Lastly, we explored a few heterocycles that resemble imidazo[1,2-*a*]pyridine to vindicate the generality of this method. Gratifyingly, 6-phenylimidazo[2,1-*b*]thiazole, 2-phenylbenzo[*d*]imidazo[2,1-*b*]thiazole, and 2-phenylimidazo[1,2-*a*]pyrimidine participated well under the standard reaction conditions, generating the corresponding acetoxymalonylated products **4u**–**w** in good to excellent yields.

**Scheme 3:**
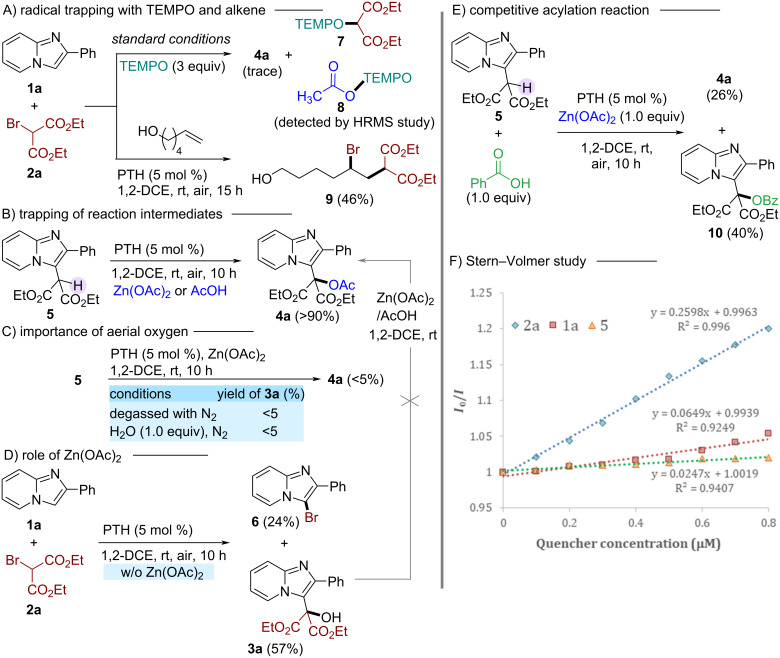
Mechanistic investigations.

Several control experiments were performed to gain insights into the mechanistic pathway of this reaction. Firstly, a radical scavenging experiment using the radical scavenger TEMPO was performed (Scheme 3A). Upon analyzing the reaction mixture of **1a** and **2a** under standard conditions in the presence of TEMPO, we found only a trace of the desired product **4a**. At the same time, a TEMPO-DEM adduct **7** and TEMPO-OAc adduct **8** were identified by the HRMS analysis of the crude reaction mixture, indicating the involvement of a malonyl radical and an acetyl radical in the course of the reaction (see Supporting Information File 1 for details). Additionally, when an aliphatic alkene, 5-hexen-1-ol was introduced into the reaction mixture under standard conditions without Zn(OAc)_2_, an ATRA product **9** was isolated, further confirming the involvement of a malonyl radical generated by the cleavage of the C–Br bond of **2a** [28]. Next, an attempt was made to identify the key intermediate of the reaction (Scheme 3B). When compound **5** was subjected to the acetylation reaction individually with Zn(OAc)_2_ and AcOH under optimized reaction conditions, the acetylated product **4a** was produced with excellent conversion (>90%). These results suggest the involvement of compound **5** as an intermediate, and Zn(OAc)_2_ or AcOH may be effective acetylating agents via generation of acetyl radicals. Control experiments under degassed conditions with or without water only delivered a trace amount (<5%) of the desired products, indicating that aerial oxygen plays a crucial role in the second catalytic cycle for the conversion of **5** to **3a** or **4a** (Scheme 3C). To determine the role of zinc acetate, a standard reaction of **1a** and **2a** in the absence of Zn(OAc)_2_ was conducted (Scheme 3D). The results showed the formation of hydroxymalonated product **3a** (57%) and bromo derivative **6** (24%). Notably, the hydroxymalonated product **3a** under the reaction conditions was not converted to the acetylated derivative **4a**, confirming **3a** is not the intermediate for the final product **4a**. So, Zn(OAc)_2_ is crucial in shutting down the formation of **6** by scavenging free bromide in the reaction as ZnBr_2_ salt (confirmed by HRMS). In addition, an excellent yield of the final product **4a** [**4a**: 94% vs (**6**, 24% + **3a**, 57%)] with additive indicates that zinc acetate plays a crucial role in activating IP towards the photoredox coupling reaction. Shifting of protons in the ^1^H NMR spectrum of 2-phenylimidazo[1,2-a]pyridine (**1a**) in the presence of Zn(OAc)_2_ in CDCl_3_ indicates a weak interaction of Zn(OAc)_2_ with **1a** (see Supporting Information File 1 for details) [20–21]. Finally, the reaction of **5** with benzoic acid and zinc acetate (in a 1:1 ratio) under standard reaction protocol resulted in the competitive formation of products **4a** and **10** (Scheme 3E), indicating the susceptibility of other acids towards this method. These results, along with the Stern–Volmer fluorescence quenching study (Scheme 3F), expressed that the photoredox reaction started with the reductive generation of a malonyl radical from bromomalonate by interaction with the photocatalyst.

**Scheme 4:**
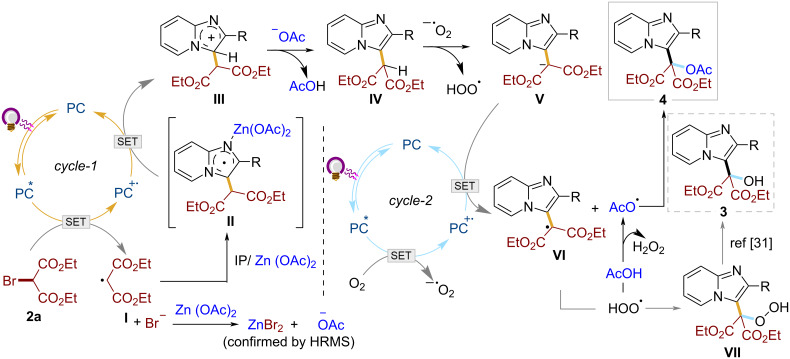
Plausible reaction mechanism.

Analyzing all the observations from the above mechanistic studies, we propose a plausible mechanism involving sequential activation and functionalization of sp^2^ and sp^3^ C–H bonds via relay catalysis (Scheme 4). The relay can be divided into two cycles; the first cycle (*cycle-1*) deals with the C(sp^2^)–H functionalization at the C-3 position of the imidazo heterocycles, while the second cycle (*cycle-2*) is all about the C(sp^3^*)*–H functionalization at the newly incorporated active methylene center.

*Cycle-1* is initiated with the reduction of bromomalonate **2a** by the photoexcited catalyst PC^*^ to malonyl radical **I**. This is followed by the Minisci-type addition of radical **I** to the imidazopyridine, preactivated by Lewis acidic Zn(OAc)_2_ [29]. **PC^∙+^** then oxidizes the resulting radical **II** to carbocation **III** which rearomatizes by losing a proton to generate the intermediate **IV** and closing the first catalytic cycle. Meanwhile, the bromide ions in the medium undergo anion exchange with the Zn(OAc)_2_ to release free acetate ions, along with the conversion into ZnBr_2_ (confirmed by HRMS). These in situ-generated free acetate ions function as a base, deprotonating carbocation **III** to produce the intermediate **IV** and AcOH.

The first step of *cycle-2* involves the oxidation of the excited photocatalyst by aerial oxygen to generate superoxide anion and **PC^∙+^**. The superoxide anion (O_2_**^·−^**) then captures the proton from the active methylene center of intermediate **IV** to generate the malonyl anion **V**, which undergoes single electron oxidation by **PC^∙+^** generating the malonyl radical **VI** [30–31]. Meanwhile, the hydroperoxy radical (**^∙^**OOH) formed, reacts with AcOH produced in *cycle-1* to give the acetoxy radical (**^∙^**OAc) and H_2_O_2_. Then, the radical recombination between AcO**^∙^** and radical **VI** furnishes the desired product **4**. In the absence of the acetoxy radical (**^∙^**OAc), the hydroperoxy radical (∙OOH) may combine with radical **VI** to produce **VII**, which then easily converts into hydroxymalonated product **3** [31].

## Conclusion

Thus, we have reported the successful C-3 acetoxymalonylation of imidazo[1,2-*a*]pyridines and related heterocycles by an organophotocatalytic relay C–H functionalization strategy with Zn(OAc)_2_ in the triple role of an activator, bromide scavenger, and acetylating agent. The developed method is heavy-metal free, as shown by the use of inexpensive PTH, as well as a base-free approach, and involves aerial oxygen to generate exciting derivatives, which may prove to be valuable in the field of radical chemistry research in future.

## Supporting Information

File 1 Experimental section and characterization of synthesized compounds.
