# Supplementary material for: Photocatalytic sequential C–H functionalization expediting acetoxymalonylation of imidazo heterocycles
Source: Beilstein J Org Chem. 2023 May 12;19:666–73. doi: 10.3762/bjoc.19.48 (PMC10186259; doi:10.3762/bjoc.19.48)

BEILSTEIN JOURNAL OF ORGANIC CHEMISTRY

Supporting Information

for

Photocatalytic sequential C–H functionalization expediting acetoxymalonylation of imidazo heterocycles

Deepak Singh, Shyamal Pramanik and Soumitra Maity

Beilstein J. Org. Chem. **2023,** *19,* 666–673. doi:10.3762/bjoc.19.48

Experimental section and characterization of synthesized compounds

License and Terms: This is a supporting information file under the terms of the Creative Commons Attribution License (https://creativecommons.org/licenses/by/4.0). Please note that the reuse, redistribution and reproduction in particular requires that the author(s) and source are credited and that individual graphics may be subject to special legal provisions.
The license is subject to the *Beilstein Journal of Organic Chemistry* terms and conditions: (https://www.beilstein-journals.org/bjoc/terms)

Table of contents

	Table of contents	*Page*
1.	General	S2
2.	Preparation of starting materials	S3
3.	Reaction optimization	S3
4.	Experimental procedures and compound characterization data	S4
5.	Mechanistic studies and control experiments	S14
6.	X-ray crystallographic data of compound **4d**	S19
7.	NMR spectra	S21

1. General

All commercially available chemicals and reagents were used without further purification unless otherwise stated. Solvents for extraction or column chromatography were of technical quality. All water used was purified via a Merck Millipore reverse osmosis purification system prior to use. All reactions were performed in oven-dried glassware under a positive pressure of nitrogen with freshly distilled anhydrous solvents.[1] Solvents were transferred via syringe and were introduced into the reaction vessels through a rubber septum. Solvents were removed under reduced pressure using IKA and Büchi rotary evaporator.

Thin-layer chromatography (TLC): The progress of the reaction was monitored by thin-layer chromatography (TLC) using SiO_2-60 UV254 coated aluminum sheets (Merck, TLC Silica gel 60 F_{254}). Visualization was achieved using UV light, iodine, and/or chemical staining with vanillin or basic potassium permanganate solutions as appropriate.

Flash column chromatography (FC): Purification of reaction mixtures was carried out with flash column chromatography on silica gel 230-400 mesh (Merck, 37–63 µm). Solvents for extraction and chromatography were of technical quality. Eluting solvent mixtures are individually reported in parenthesis.

NMR spectra: Proton, carbon, and fluorine nuclear magnetic resonance (^{1}H, ^{13}C, and ^{19}F NMR) spectra were recorded on a Bruker Avance III HD (400, 101, and 377 MHz) spectrometer at 25 °C. Chemical shifts (δ) are given in ppm and reported as follows: multiplicity (s (singlet), d (doublet), t (triplet), q (quartet), dd (doublet of doublets), dt (doublet of triplets), and m (multiplet)), coupling constants (J) in Hz, number of protons; suggested assignment. The residual deuterated solvent was used as internal standard ($CDCl_3$: δ_H = 7.26 ppm; δ_C = 77.16 ppm).

Melting point (Mp): Melting points were measured using Tempstar melting point instrument Remco-Kolkata apparatus using open glass capillaries and are reported uncorrected.

High-resolution mass spectrometry (HRMS): HRMS were recorded using a QTOF micro MS system by ESI technique.

GC–MS: GC–MS analysis was done by a Thermo Scientific ISQ 7000 single quadrupole mass spectrometer fitted with TRACE 1310 gas chromatograph using a TG-5MS column (30 m × 0.25 mm × 0.25 µm).

Photoreactions: Photoreactions were carried out in borosilicate made culture tube using light source (PAR38 12W blue LED bulb / Kessil violet LEDs 390 nm).

UV–vis spectroscopy: UV–vis absorption spectra were recorded using a Shimadzu UV Spectrophotometer (model: UV-1800).

Luminescence spectrometer: Fluorescence quenching studies were carried out using a Shimadzu RF-6000 spectrophotometer (model no.- A40246002251SA).

[1] W. L. F. Armarego, C. Chai. *Purification of Laboratory Chemicals*; 7th ed. Butterworth-Heinemann: Oxford, **2012**.

2. Preparation of starting materials

2.1. General procedure for the synthesis of 2-arylimidazo[1,2-*a*]pyridines: All 2-arylimidazo[1,2-*a*]pyridines were prepared either from the corresponding methyl ketone and 2-aminopyridines (or compounds **1a**–**q** and **w** also prepared following the same procedure),[2] or from the corresponding 2-bromoacetophenone and 2-aminopyridines (for compounds **1r**,[3] **1u**[4] and **v**[5]) following the reported procedure.

3. Reaction optimization [a]:

General procedure for optimization of reaction conditions: An oven-dried culture tube equipped with a magnetic stirring bar was charged with 2-phenylimidazo[1,2-*a*]pyridine (**1a**, 39 mg, 0.2 mmol, 1.0 equiv), diethyl bromomalonate (**2a**, 96 mg, 0.4 mmol, 2.0 equiv), photocatalyst (5 mol %), additive (0.4 mmol, 2.0 equiv), and dry solvent (2 mL). The resulting reaction mixture was stirred at room temperature under open atmosphere conditions using 12 W blue LEDs or 390 nm violet LEDs for 10 h. A fan cooling was also included to maintain the reaction at room temperature.

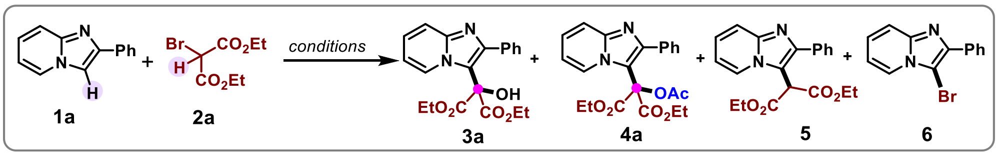

entry	catalyst	solvent	additive	yield (%)[b] **3a**: **4a**: **5**: **6**
1[c]	4-CzIPN	CH_3CN	-	0: 0: 54: 28
2	4-CzIPN	CH_3CN	-	47: 0: 0: 22
3	4-CzIPN	CH_3CN	$Zn(OAc)_2$	0: 38: 0: 0
4	Rose Bengal	CH_3CN	$Zn(OAc)_2$	-
5	Eosin-Y	CH_3CN	$Zn(OAc)_2$	-
6	Rhodamine-B	CH_3CN	$Zn(OAc)_2$	-
7[d]	PTH	CH_3CN	$Zn(OAc)_2$	0: 52: 0: 0
8	PTH	1,4-dioxane	$Zn(OAc)_2$	0: 34: 0: 0
9	PTH	DMF	$Zn(OAc)_2$	0: 25: 0: 0
10	PTH	Toluene	$Zn(OAc)_2$	0: 18: 0: 0
11	PTH	1,2-DCE	$Zn(OAc)_2$	0: 70: 0: 0
12[e]	PTH	1,2-DCE	$Zn(OAc)_2$	0: 94: 0: 0
13[f]	PTH	1,2-DCE	$Zn(OAc)_2$	0: 52: 0: 0
14[g]	PTH	1,2-DCE	$Zn(OAc)_2$	0: 88: 0: 0
15	PTH	1,2-DCE	AcOH	0: 64: 0: 0
16	-	1,2-DCE	$Zn(OAc)_2$	-
17[h]	PTH	1,2-DCE	$Zn(OAc)_2$	-

[2] Mohan, D. C.; Donthiri, R. R.; Rao, S. N.; Adimurthy, S. *Adv. Syn. Cat.* **2013**, *355*, 2217.
[3] Cai, S.; Yang. X; Chen, P; Liu, X; Zhou, J; Zhang, H. *Bioorg. Chem.* **2020**, *94*, 103356.
[4] Baig, M. F.; Nayak, V. L.; Budaganaboyina, P.; Mullagiri, K.; Sunkari. S.; Gour, J.; Kamal, A. *Bioorg. Chem.* **2018**, *77*, 515.
[5] Mishra, S.; Monir, K.; Mitra, S.; Hajra, A. *Org. lett.* **2014**, *16*, 6084.

18	PTH	1,2-DCE	-	57: 0: 0: 24
19[i]	PTH	1,2-DCE	$Zn(OAc)_2$	-
20[j]	PTH	1,2-DCE	$Zn(OAc)_2$	-
21[k]	PTH	1,2-DCE	$Zn(OAc)_2$	0: trace: 0: 0

[a]Reaction conditions: **1a** (0.2 mmol), **2a** (0.4 mmol), catalyst (5 mol %), additive (0.4 mmol) in dry solvent (2 mL) under aerobic conditions, irradiation with 12 W blue LEDs for 10 h. [b]Isolated yield. [c]Under N_2 atmosphere. [d]Irradiation with violet LEDs (λ_{max}= 390 nm), [e]3.0 equiv of zinc acetate used. [f]2 mol % catalyst used. [g]10 mol % catalyst used. [h]In the dark, without light source. [i]Blue LEDs used as light source. [j]Green LEDs used as light source. [k]Using house hold white CFL bulb as light source.

4. Experimental procedures & compound characterization data

4.1. General procedure for the synthesis of acetoxymalonylated imidazo-heterocycles: An oven-dried culture tube equipped with a magnetic stirring bar was charged with 2-substituted imidazo-heterocycles **1** (0.2 mmol, 1.0 equiv), active bromo-methylene **2** (0.4 mmol, 2.0 equiv), additive $Zn(OAc)_2$ (132 mg, 0.6 mmol, 3.0 equiv) and photocatalyst **PTH** (5 mol %) in dry 1,2-DCE (2 mL). The resulting reaction mixture was stirred at room temperature under open atmosphere using 390 nm violet LEDs for 10 h. A fan cooling was also included to maintain the reaction at room temperature. Completion of the reaction was confirmed by TLC. Then, the crude reaction mixture was poured into H_2O (10 mL) and extracted with CH_2Cl_2 (3 × 6 mL). The combined organic layer was washed with brine solution and dried using oven dried anhydrous Na_2SO_4. Then the mixture was concentrated in a rotary evaporator under reduced pressure. The crude residue was then purified by column chromatography to obtain the desired product **4**.

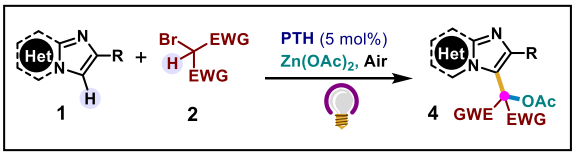

4.2. Compound characterization data

Diethyl 2-acetoxy-2-(2-phenylimidazo[1,2-*a*]pyridin-3-yl)malonate (4a):

Yield: 94% (77 mg).

Nature: yellow solid

Mp: 121-123 °C

R_f value = 0.2 [EtOAc: Petroleum ether = 3:7 (v/v)].

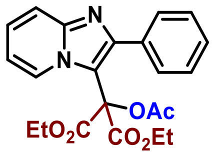

4a

^{1}H NMR (400 MHz, $CDCl_3$) δ (ppm): 8.28 (dd, J = 4.6, 3.5 Hz, 1H), 7.73 (d, J = 9.1 Hz, 1H), 7.60 – 7.58 (m, 2H), 7.41 (d, J = 7.1 Hz, 3H), 7.33 (ddd, J = 9.1, 6.8, 1.1 Hz, 1H), 6.90 (td, J = 6.9, 1.2 Hz, 1H), 3.88 (tdd, J = 10.7, 7.1, 3.6 Hz, 4H), 2.14 (s, 3H), 1.13 (t, J = 7.1 Hz, 6H).

^{13}C{^{1}H} NMR (101 MHz, $CDCl_3$) δ (ppm): 168.8, 164.2, 146.0, 145.1, 133.7, 130.3, 128.7, 127.9, 126.4, 126.3, 117.8, 113.4, 113.3, 80.0, 63.3, 20.5, 13.8.

HRMS (ESI) *m/z* calcd for $C_{22}H_{23}N_2O_6$ $[M+H]^+$: 411.1556; found: 411.1554

**Diethyl 2-acetoxy-2-(2-(*p*-tolyl)imidazo[1,2-*a*]pyridin-3-yl)malonate (4b):**	
**Yield:** 95% (81 mg).<br>**Nature:** white solid<br>**Mp:** 168-170 °C<br>**Rf value** = 0.2 [EtOAc: Petroleum ether = 3:7 (v/v)].	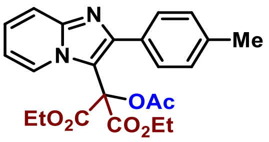<br>**4b**

^{1}**H NMR (400 MHz, CDCl$_3$) *δ* (ppm):** 8.27 (d, *J* = 7.1 Hz, 1H), 7.73 (d, *J* = 9.0 Hz, 1H), 7.48 (d, *J* = 8.1 Hz, 2H), 7.32 (ddd, *J* = 8.8, 6.7, 1.0 Hz, 1H), 7.22 (d, *J* = 7.9 Hz, 2H), 6.89 (td, *J* = 6.9, 1.2 Hz, 1H), 3.94 – 3.85 (m, 4H), 2.38 (s, 3H), 2.14 (s, 3H), 1.13 (t, *J* = 7.1 Hz, 6H).

^{13}**C{^{1}H} NMR (101 MHz, CDCl$_3$) *δ* (ppm):** 168.8, 164.2, 146.1, 145.0, 138.5, 130.6, 130.2, 128.5, 126.4, 126.2, 117.7, 113.3, 113.2, 80.1, 63.3, 21.5, 20.5, 13.8.

HRMS (ESI) *m/z* calcd for $C_{23}H_{25}N_2O_6$ $[M+H]^+$: 425.1713; found: 425.1710

**Diethyl 2-acetoxy-2-(2-(4-bromophenyl)imidazo[1,2-*a*]pyridin-3-yl)malonate (4c):**	
**Yield:** 92% (90 mg).<br>**Nature:** white solid<br>**Mp:** 160-162 °C<br>**Rf value** = 0.2 [EtOAc: Petroleum ether = 3:7 (v/v)].	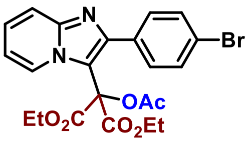<br>**4c**

^{1}**H NMR (400 MHz, CDCl$_3$) *δ* (ppm):** 8.27 (dd, *J* = 8.1, 0.9 Hz, 1H), 7.70 (dd, *J* = 5.6, 4.5 Hz, 1H), 7.57 – 7.54 (m, 2H), 7.49 – 7.46 (m, 2H), 7.33 (ddd, *J* = 9.1, 6.8, 1.1 Hz, 1H), 6.90 (td, *J* = 6.9, 1.2 Hz, 1H), 3.98 – 3.89 (m, 4H), 2.14 (s, 3H), 1.14 (t, *J* = 7.1 Hz, 6H).

^{13}**C{^{1}H} NMR (101 MHz, CDCl$_3$) *δ* (ppm):** 168.7, 164.1, 145.3, 144.9, 132.9, 131.9, 131.0, 126.4, 126.3, 123.1, 117.8, 113.5, 113.3, 79.9, 63.4, 20.5, 13.8.

HRMS (ESI) *m/z* calcd for $C_{22}H_{22}BrN_2O_6$ $[M+H]^+$: 489.0661; found: 489.0668

**Diethyl 2-acetoxy-2-(2-(4-fluorophenyl)imidazo[1,2-*a*]pyridin-3-yl)malonate (4d):**	
**Yield:** 81% (70 mg).<br>**Nature:** white solid<br>**Mp:** 134-136 °C<br>**Rf value** = 0.2 [EtOAc: Petroleum ether = 3:7 (v/v)].	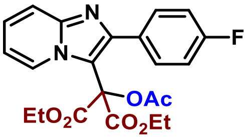<br>**4d**

^{1}**H NMR (400 MHz, CDCl$_3$) *δ* (ppm):** 8.26 (d, *J* = 7.2 Hz, 1H), 7.68 (d, *J* = 9.1 Hz, 1H), 7.57 (dd, *J* = 8.7, 5.5 Hz, 2H), 7.31 (ddd, *J* = 8.9, 6.7, 1.0 Hz, 1H), 7.10 (t, *J* = 8.7 Hz, 2H), 6.88 (td, *J* = 7.0, 1.1 Hz, 1H), 3.97 – 3.87 (m, 4H), 2.14 (s, 3H), 1.14 (t, *J* = 7.2 Hz, 6H).

^{13}**C{^{1}H} NMR (101 MHz, CDCl$_3$) *δ* (ppm):** 168.8, 164.2, 163.1 (d, *J* = 248.8 Hz), 145.3 (d, *J* = 4.2 Hz), 132.1 (d, *J* = 8.6 Hz), 130.1, 126.3 (d, *J* = 21.3 Hz), 117.8, 114.9, 114.7, 113.4, 113.2, 79.9, 63.3, 20.5, 13.8.

^{19}**F NMR (376 MHz, CDCl$_3$) δ (ppm):** −113.1

HRMS (ESI) *m/z* calcd for $C_{22}H_{22}FN_2O_6$ $[M+H]^+$: 429.1462; found: 429.1461

Diethyl 2-acetoxy-2-(2-(4-chlorophenyl)imidazo[1,2-*a*]pyridin-3-yl)malonate (4e):

Yield: 89% (79 mg).

Nature: white solid

Mp: 158-160 °C

R$_f$ value = 0.2 [EtOAc: Petroleum ether = 3:7 (v/v)].

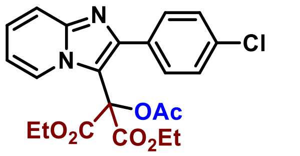

4e

^{1}H NMR (400 MHz, CDCl$_3$) *δ* (ppm): 8.28 – 8.26 (m, 1H), 7.68 (dd, *J* = 10.0, 0.9 Hz, 1H), 7.55 – 7.52 (m, 2H), 7.41 – 7.38 (m, 2H), 7.31 (ddd, *J* = 9.1, 6.8, 1.2 Hz, 1H), 6.89 (td, *J* = 6.9, 1.2 Hz, 1H), 3.97 – 3.89 (m, 4H), 2.14 (s, 3H), 1.14 (t, *J* = 7.1 Hz, 6H).

^{13}C{^{1}H} NMR (101 MHz, CDCl$_3$) *δ* (ppm): 168.7, 164.1, 145.3, 145.0, 134.8, 132.6, 131.63, 128.0, 126.4, 126.3, 117.9, 113.5, 113.2, 79.9, 63.4, 20.5, 13.8.

HRMS (ESI) *m/z* calcd for $C_{22}H_{22}ClN_2O_6$ [M+H]$^+$: 445.1166; found: 445.1165

Diethyl 2-acetoxy-2-(2-(4-cyanophenyl)imidazo[1,2-*a*]pyridin-3-yl)malonate (4f):

Yield: 79% (69 mg).

Nature: yellow solid

Mpt: 133-135 °C

R$_f$ value = 0.2 [EtOAc: Petroleum ether = 3:7 (v/v)].

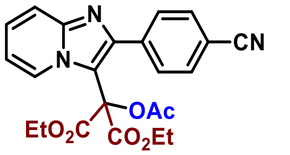

4f

^{1}H NMR (400 MHz, CDCl$_3$) *δ* (ppm): 8.33 – 8.31 (m, 1H), 7.79 – 7.72 (m, 5H), 7.40 (ddd, *J* = 8.8, 6.9, 1.0 Hz, 1H), 6.97 (td, *J* = 6.9, 1.1 Hz, 1H), 3.99 – 3.91 (m, 4H), 2.12 (s, 3H), 1.15 (t, *J* = 7.2 Hz, 6H).

^{13}C{^{1}H} NMR (101 MHz, CDCl$_3$) *δ* (ppm): 168.6, 163.9, 145.1, 143.5, 138.4, 131.7, 131.1, 127.2, 126.6, 118.7, 117.8, 114.2, 113.9, 112.5, 79.6, 63.6, 20.4, 13.8.

HRMS (ESI) *m/z* calcd for $C_{23}H_{22}N_3O_6$ [M+H]$^+$: 436.1509; found: 436.1503

Diethyl 2-acetoxy-2-(2-(4-methoxyphenyl)imidazo[1,2-*a*]pyridin-3-yl)malonate (4g):

Yield: 85% (75 mg).

Nature: white solid

Mp: 177-179 °C

R$_f$ value = 0.2 [EtOAc: Petroleum ether = 3:7 (v/v)].

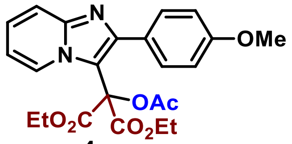

4g

^{1}H NMR (400 MHz, CDCl$_3$) *δ* (ppm): 8.34 (d, *J* = 7.1 Hz, 1H), 8.01 (d, *J* = 8.0 Hz, 1H), 7.58 – 7.56 (m, 2H), 7.51 – 7.47 (m, 1H), 7.04 (t, *J* = 7.0 Hz, 1H), 7.00 – 6.96 (m, 2H), 3.99 – 3.90 (m, 4.3 Hz, 4H), 3.85 (s, 3H), 2.16 (s, 3H), 1.15 (t, *J* = 7.2 Hz, 6H).

^{13}C{^{1}H} NMR (101 MHz, CDCl$_3$) *δ* (ppm): 168.8, 164.3, 160.0, 146.0, 145.2, 131.5, 126.4, 126.2, 126.0, 117.7, 113.3, 113.2, 113.0, 80.1, 63.3, 55.5, 20.5, 13.8.

HRMS (ESI) *m/z* calcd for $C_{23}H_{25}N_2O_7$ [M+H]$^+$: 441.1662; found: 441.1677

Diethyl 2-acetoxy-2-(2-(2-fluorophenyl)imidazo[1,2-*a*]pyridin-3-yl)malonate (4h):

Yield: 93% (80 mg).

Nature: yellow solid

Mp: 94-96 °C

R$_f$ value = 0.2 [EtOAc: Petroleum ether = 3:7 (v/v)].

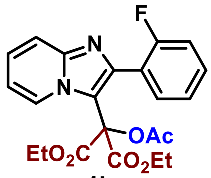

4h

^{1}H NMR (400 MHz, CDCl$_3$) *δ* (ppm): 8.32 – 8.30 (m, 1H), 7.74 (d, *J* = 9.0 Hz, 1H), 7.46 (td, *J* = 7.4, 1.8 Hz, 1H), 7.39 (tdd, *J* = 7.2, 5.1, 1.8 Hz, 1H), 7.33 (ddd, *J* = 8.8, 6.8, 1.0 Hz, 1H), 7.17 (ddd, *J* = 18.5, 8.6, 0.9 Hz, 2H), 6.91 (td, *J* = 7.0, 1.1 Hz, 1H), 3.92 (bs, 4H), 2.06 (s, 3H), 1.14 (t, *J* = 7.1 Hz, 6H).

^{13}C{^{1}H} NMR (101 MHz, CDCl$_3$) *δ* (ppm): 168.8, 164.2, 160.7 (d, *J* = 249.5 Hz), 145.5, 140.1, 132.9 (d, *J* = 2.0 Hz), 130.7 (d, *J* = 8.4 Hz), 126.1 (d, *J* = 10.4 Hz), 123.4 (d, *J* = 3.2 Hz), 122.3 (d, *J* = 15.9 Hz), 118.0, 115.4 (d, *J* = 21.7 Hz), 114.7, 113.2, 79.8, 63.2, 20.3, 13.7.

^{19}F NMR (376 MHz, CDCl$_3$) δ (ppm): −112.4

HRMS (ESI) *m/z* calcd for $C_{22}H_{22}FN_2O_6$ [M+H]$^+$: 429.1462; found: 429.1464

Diethyl 2-acetoxy-2-(2-(3-bromophenyl)imidazo[1,2-*a*]pyridin-3-yl)malonate (4i):

Yield: 74% (73 mg).

Nature: yellow gummy liquid

R$_f$ value = 0.2 [EtOAc: Petroleum ether = 3:7 (v/v)].

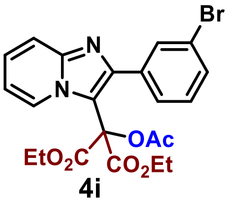

4i

^{1}H NMR (400 MHz, CDCl$_3$) *δ* (ppm): 8.30 – 8.28 (m, 1H), 7.77 (t, *J* = 1.8 Hz, 1H), 7.71 (dd, *J* = 5.5, 4.5 Hz, 1H), 7.54 (ddt, *J* = 6.7, 3.1, 1.0 Hz, 2H), 7.35 – 7.30 (m, 2H), 6.90 (td, *J* = 6.9, 1.3 Hz, 1H), 3.96 (m, 4H), 2.14 (s, 3H), 1.19 (t, *J* = 7.1 Hz, 6H).

^{13}C{^{1}H} NMR (101 MHz, CDCl$_3$) *δ* (ppm): 168.7, 164.2, 145.3, 144.5, 136.0, 133.3, 131.6, 129.5, 128.9, 126.4, 126.3, 121.8, 117.9, 113.7, 113.4, 79.8, 63.5, 20.4, 13.8.

HRMS (ESI) *m/z* calcd for $C_{22}H_{22}BrN_2O_6$ [M+H]$^+$: 489.0661; found: 489.0695

Diethyl 2-(2-([1,1'-biphenyl]-4-yl)imidazo[1,2-*a*]pyridin-3-yl)-2-acetoxymalonate (4j):

Yield: 77% (75 mg).

Nature: white solid

Mp: 197-199 °C

R$_f$ value = 0.2 [EtOAc: Petroleum ether = 3:7 (v/v)].

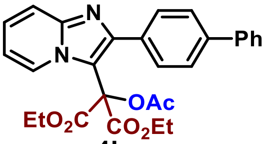

4j

^{1}H NMR (400 MHz, CDCl$_3$) *δ* (ppm): 8.30 – 8.28 (m, 1H), 7.73 – 7.71 (m, 1H), 7.68 (s, 4H), 7.66 – 7.63 (m, 2H), 7.46 (t, *J* = 7.5 Hz, 2H), 7.39 – 7.30 (m, 2H), 6.90 (td, *J* = 6.9, 1.2 Hz, 1H), 3.97 – 3.88 (m, 4H), 2.15 (s, 3H), 1.15 (t, *J* = 7.2 Hz, 6H).

^{13}C{^{1}H} NMR (101 MHz, CDCl$_3$) δ (ppm): 168.8, 164.3 145.9, 145.3, 141.3, 140.8, 133.0, 130.7, 128.9, 127.6, 127.2, 126.5, 126.4, 126.0, 117.9, 113.4, 113.1, 80.1, 63.3, 20.5, 13.8.

HRMS (ESI) *m/z* calcd for $C_{28}H_{27}N_2O_6$ [M+H]$^+$: 487.1869; found: 487.1865

Diethyl 2-acetoxy-2-(2-(naphthalen-2-yl)imidazo[1,2-*a*]pyridin-3-yl)malonate (4k):

Yield: 82% (75 mg).

Nature: white solid

Mp: 158-160 °C

R$_f$ value = 0.2 [EtOAc: Petroleum ether = 3:7 (v/v)].

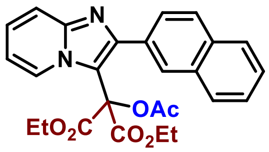

4k

^{1}H NMR (400 MHz, CDCl$_3$) δ (ppm): 8.32 (d, J = 7.2 Hz, 1H), 8.08 (s, 1H), 7.90 (d, J = 8.5 Hz, 1H), 7.88 – 7.84 (m, 2H), 7.77 – 7.73 (m, 2H), 7.52 – 7.50 (m, 2H), 7.34 (ddd, J = 9.0, 6.8, 1.1 Hz, 1H), 6.92 (td, J = 6.9, 1.2 Hz, 1H), 3.79 (q, J = 7.1 Hz, 4H), 2.12 (s, 3H), 1.10 (t, J = 7.2 Hz, 6H).

^{13}C{^{1}H} NMR (101 MHz, CDCl$_3$) δ (ppm): 168.8, 164.30, 146.0, 145.3, 133.2, 132.7, 131.1, 129.9, 128.4, 127.8, 127.7, 127.6, 126.6, 126.5, 126.4, 126.3, 117.8, 113.8, 113.3, 80.1, 63.3, 20.5, 13.7.

HRMS (ESI) *m/z* calcd for $C_{26}H_{25}N_2O_6$ [M+H]$^+$: 461.1713; found: 461.1714

Diethyl 2-acetoxy-2-(8-methyl-2-phenylimidazo[1,2-*a*]pyridin-3-yl)malonate (4l):

Yield: 78% (66 mg).

Nature: light yellow oil

R$_f$ value = 0.2 [EtOAc: Petroleum ether = 3:7 (v/v)].

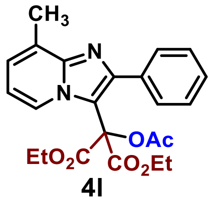

4l

^{1}H NMR (400 MHz, CDCl$_3$) δ (ppm): 8.13 (d, J = 7.0 Hz, 1H), 7.58 (dd, J = 7.9, 1.6 Hz, 2H), 7.43 – 7.36 (m, 3H), 7.09 (dd, J = 6.9, 0.9 Hz, 1H), 6.78 (t, J = 7.0 Hz, 1H), 3.91 – 3.81 (m, 4H), 2.64 (s, 3H), 2.13 (s, 3H), 1.13 (t, J = 7.2 Hz, 6H).

^{13}C{^{1}H} NMR (101 MHz, CDCl$_3$) δ (ppm): 168.8, 164.3, 145.8, 145.6, 134.2, 130.5, 128.5, 127.9, 127.8, 124.8, 124.0, 113.7, 113.1, 80.2, 63.2, 20.5, 17.4, 13.8.

HRMS (ESI) *m/z* calcd for $C_{23}H_{25}N_2O_6$ [M+H]$^+$: 425.1713; found: 425.1707

Diethyl 2-acetoxy-2-(7-methyl-2-phenylimidazo[1,2-*a*]pyridin-3-yl)malonate (4m):

Yield: 82% (70 mg).

Nature: light yellow solid

Mp: 135-137 °C

R$_f$ value = 0.2 [EtOAc: Petroleum ether = 3:7 (v/v)].

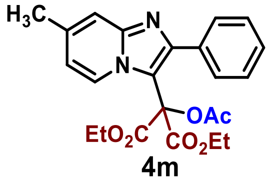

4m

**^{1}H NMR (400 MHz, CDCl$_3$) *δ* (ppm):** 8.15 (d, *J* = 7.1 Hz, 1H), 7.59 – 7.57 (m, 2H), 7.50 (s, 1H), 7.43 – 7.37 (m, 3H), 6.74 (dd, *J* = 7.2, 1.7 Hz, 1H), 3.93 – 3.82 (m, 4H), 2.43 (s, 3H), 2.13 (s, 3H), 1.12 (t, *J* = 7.1 Hz, 6H).<br>**^{13}C{^{1}H} NMR (101 MHz, CDCl$_3$) *δ* (ppm):** 168.8, 164.2, 145.5, 145.4, 137.7, 133.6, 130.3, 128.7, 127.9, 125.6, 116.0, 112.9, 80.0, 63.3, 21.4, 20.5, 13.8.<br>**HRMS** (ESI) *m/z* calcd for $C_{23}H_{25}N_2O_6$ [M+H]$^+$: 425.1713; found: 425.1707

**Diethyl 2-acetoxy-2-(6-iodo-2-phenylimidazo[1,2-*a*]pyridin-3-yl)malonate (4n):**	
**Yield:** 58% (62 mg).<br>**Nature:** white solid<br>**Mp:** 143-145 °C<br>**R$_f$ value:** 0.2 [EtOAc: Petroleum ether = 2:8 (v/v)].	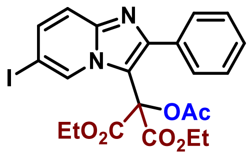<br><br>**4n**
**^{1}H NMR (400 MHz, CDCl$_3$) *δ* (ppm):** 8.60 (s, 1H), 7.67 (d, *J* = 9.2 Hz, 1H), 7.61 – 7.57 (m, 3H), 7.45 – 7.43 (m, 3H), 3.94 (q, *J* = 7.1 Hz, 4H), 2.12 (s, 3H), 1.17 (t, *J* = 7.1 Hz, 6H).<br>**^{13}C{^{1}H} NMR (101 MHz, CDCl$_3$) *δ* (ppm):** 168.6, 163.7, 144.4, 142.6, 135.7, 131.9, 131.3, 130.3, 129.5, 128.2, 117.9, 113.9, 79.5, 63.7, 20.4, 13.8.<br>**HRMS** (ESI) *m/z* calcd for $C_{22}H_{21}IN_2O_6$ [M+H]$^+$: 537.0523; found: 537.0517	

**Diethyl 2-acetoxy-2-(6-bromo-2-phenylimidazo[1,2-*a*]pyridin-3-yl)malonate (4o):**	
**Yield:** 79% (77 mg).<br>**Nature:** white solid<br>**Mp:** 96-98 °C<br>**R$_f$ value** = 0.2 [EtOAc: Petroleum ether = 2:8 (v/v)].	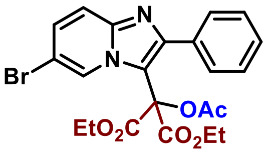<br><br>**4o**
**^{1}H NMR (400 MHz, CDCl$_3$) *δ* (ppm):** 8.48 (s, 1H), 7.74 (d, *J* = 9.5 Hz, 1H), 7.61 – 7.58 (m, 2H), 7.44 (ddd, *J* = 9.5, 5.3, 1.9 Hz, 4H), 3.93 (m, 4H), 2.12 (s, 3H), 1.16 (t, *J* = 7.2 Hz, 6H).<br>**^{13}C{^{1}H} NMR (101 MHz, CDCl$_3$) *δ* (ppm):** 168.7, 163.9, 146.6, 143.5, 133.2, 130.2, 129.8, 128.1, 127.9, 126.7, 118.3, 114.0, 107.9, 79.7, 63.5, 20.4, 13.8.<br>**HRMS** (ESI) *m/z* calcd for $C_{22}H_{22}BrN_2O_6$ [M+H]$^+$: 489.0661; found: 489.0656	

Diethyl 2-acetoxy-2-(6-bromo-7-methyl-2-phenylimidazo[1,2-*a*]pyridin-3-yl)malonate (4p):

Yield: 59% (60 mg).

Nature: light yellow solid

Mp: 137-139 °C

R_f value = 0.2 [EtOAc: Petroleum ether = 3:7 (v/v)].

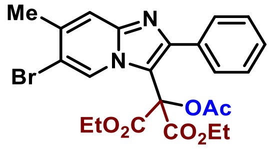

4p

^{1}H NMR (400 MHz, CDCl$_3$) δ (ppm): 8.15 (d, J = 7.1 Hz, 1H), 7.58 (dd, J = 7.8, 1.7 Hz, 2H), 7.50 (s, 1H), 7.41 – 7.39 (m, 2H), 6.73 (dd, J = 7.2, 1.7 Hz, 1H), 3.93 – 3.82 (m, 4H), 2.43 (s, 3H), 2.13 (s, 3H), 1.13 (t, J = 7.1 Hz, 6H).

^{13}C{^{1}H} NMR (101 MHz, CDCl$_3$) δ (ppm): 168.8, 164.3, 145.6, 145.4, 137.7, 133.7, 130.3, 128.7, 127.8, 125.6, 116.0, 115.9, 112.8, 80.0, 63.2, 21.4, 20.5, 13.8.

HRMS (ESI) *m/z* calcd for $C_{23}H_{24}BrN_2O_6$ [M+H]$^+$: 503.0818; found: 503.0812

Diethyl 2-acetoxy-2-(6,8-dibromo-2-phenylimidazo[1,2-*a*]pyridin-3-yl)malonate (4q):

Yield: 51% (58 mg).

Nature: yellow gummy liquid

R_f value = 0.2 [EtOAc: Petroleum ether = 3:7 (v/v)].

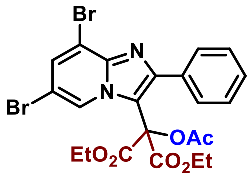

4q

^{1}H NMR (400 MHz, CDCl$_3$) δ (ppm): 8.43 (d, J = 1.5 Hz, 1H), 7.66 (d, J = 1.5 Hz, 1H), 7.59 – 7.56 (m, 2H), 7.40 (dd, J = 5.0, 2.1 Hz, 3H), 3.94 – 3.89 (m, 4H), 2.11 (s, 3H), 1.16 (t, J = 7.2 Hz, 6H).

^{13}C{^{1}H} NMR (101 MHz, CDCl$_3$) δ (ppm): 168.6, 163.8, 147.9, 142.1, 133.5, 131.0, 130.5, 128.9, 127.9, 125.9, 115.7, 112.4, 106.4, 79.8, 63.5, 20.4, 13.8.

HRMS (ESI) *m/z* calcd for $C_{22}H_{21}Br_2N_2O_6$ [M+H]$^+$: 566.9766; found: 566.9761

Diethyl 2-acetoxy-2-(2-(ethoxycarbonyl)imidazo[1,2-*a*]pyridin-3-yl)malonate (4r):

Yield: 56% (45 mg).

Nature: light yellow oil

R_f value = 0.3 [EtOAc: Petroleum ether = 6:4 (v/v)]

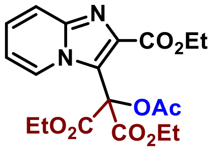

4r

^{1}H NMR (400 MHz, CDCl$_3$) δ (ppm): 8.18 – 8.16 (m, 1H), 7.75 – 7.72 (m, 1H), 7.32 (ddd, J = 9.0, 6.6, 1.1 Hz, 1H), 6.90 (td, J = 7.0, 1.2 Hz, 1H), 4.42 (t, J = 7.2 Hz, 2H), 4.38 – 4.25 (m, 4H), 2.20 (s, 3H), 1.42 (t, J = 7.1 Hz, 3H), 1.25 (t, J = 7.1 Hz, 6H).

^{13}C{^{1}H} NMR (101 MHz, CDCl$_3$) δ (ppm): 168.8, 164.3, 163.4, 145.2, 136.6, 126.8, 126.6, 119.9, 119.2, 113.9, 79.7, 63.6, 61.8, 20.8, 14.4, 13.9.

HRMS (ESI) *m/z* calcd for $C_{19}H_{22}N_2NaO_8$ [M+Na]$^+$: 429.1274; found: 429.1280

Dimethyl 2-acetoxy-2-(2-phenylimidazo[1,2-*a*]pyridin-3-yl)malonate (4s):

Yield: 92% (70 mg).

Nature: white solid

Mp: 125-127 °C

R_f value = 0.2 [EtOAc: Petroleum ether = 4:6 (v/v)].

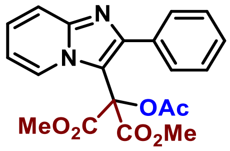

4s

^{1}H NMR (400 MHz, $CDCl_3$) δ (ppm): 8.23 (dd, *J* = 4.6, 3.5 Hz, 1H), 7.68 (dd, *J* = 5.5, 4.5 Hz, 1H), 7.57 – 7.54 (m, 2H), 7.41 (tdd, *J* = 6.8, 4.6, 2.5 Hz, 3H), 7.32 – 7.27 (m, 1H), 6.88 (td, *J* = 6.9, 1.3 Hz, 1H), 3.47 (s, 6H), 2.15 (s, 3H).

^{13}C{^{1}H} NMR (101 MHz, $CDCl_3$) δ (ppm): 168.9, 164.8, 146.8, 145.5, 134.1, 130.2, 128.5, 127.9, 126.1, 125.8, 118.1, 113.1, 112.9, 79.9, 53.7, 20.5.

HRMS (ESI) *m/z* calcd for $C_{20}H_{19}N_2O_6$ [M+H]$^+$: 383.1243; found: 383.1245

Diisopropyl 2-acetoxy-2-(2-phenylimidazo[1,2-*a*]pyridin-3-yl)malonate (4t):

Yield: 72% (63 mg).

Nature: light yellow oil

R_f value = 0.3 [EtOAc: Petroleum ether = 4:6 (v/v)].

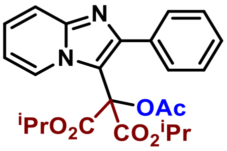

4t

^{1}H NMR (400 MHz, $CDCl_3$) δ (ppm): 8.30 (dt, *J* = 7.1, 1.1 Hz, 1H), 7.66 – 7.61 (m, 3H), 7.42 – 7.37 (m, 3H), 7.29 – 7.24 (m, 1H), 6.84 (td, *J* = 6.9, 1.3 Hz, 1H), 4.70 (dt, *J* = 12.5, 6.3 Hz, 2H), 2.08 (s, 3H), 1.12 (d, *J* = 6.3 Hz, 6H), 1.07 (d, *J* = 6.3 Hz, 6H).

^{13}C{^{1}H} NMR (101 MHz, $CDCl_3$) δ (ppm): 168.7, 163.9, 146.5, 145.5, 134.6, 130.3, 128.4, 127.8, 126.5, 125.6, 117.9, 113.4, 112.6, 80.5, 71.8, 21.4, 21.3, 20.4.

HRMS (ESI) *m/z* calcd for $C_{24}H_{27}N_2O_6$ [M+H]$^+$: 439.1869; found: 439.1859

Diethyl 2-acetoxy-2-(6-phenylimidazo[2,1-*b*]thiazol-5-yl)malonate (4u):

Yield: 84% (70 mg).

Nature: light yellow oil

R_f value = 0.2 [EtOAc: Petroleum ether = 3:7 (v/v)].

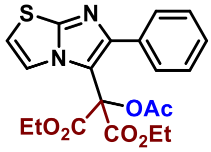

4u

^{1}H NMR (400 MHz, $CDCl_3$) δ (ppm): 7.62 (d, *J* = 4.6 Hz, 1H), 7.57 – 7.55 (m, 2H), 7.41 – 7.35 (m, 3H), 6.85 (d, *J* = 4.7 Hz, 1H), 4.02 – 3.92 (m, 4H), 2.03 (s, 3H), 1.14 (t, *J* = 7.1 Hz, 6H).

^{13}C{^{1}H} NMR (101 MHz, $CDCl_3$) δ (ppm): 169.2, 164.1, 150.7, 147.6, 133.8, 130.1, 128.5, 127.9, 121.5, 115.7, 112.0, 79.5, 63.3, 20.4, 13.7.

HRMS (ESI) *m/z* calcd for $C_{20}H_{21}N_2O_6S$ [M+H]$^+$: 417.1120; found: 417.1115

Diethyl 2-acetoxy-2-(2-phenylbenzo[*d*]imidazo[2,1-*b*]thiazol-3-yl)malonate (4v):

Yield: 65% (60 mg).

Nature: light yellow oil

R_f value = 0.2 [EtOAc: Petroleum ether = 2:8 (v/v)].

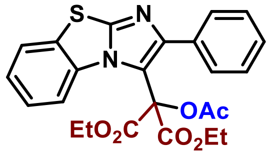

4v

^{1}H NMR (400 MHz, CDCl$_3$) δ (ppm): 8.02 (d, J = 8.1 Hz, 1H), 7.72 (dd, J = 7.9, 1.1 Hz, 1H), 7.64 – 7.61 (m, 2H), 7.47 – 7.35 (m, 5H), 3.84 (bs, 4H), 2.16 (s, 3H), 1.15 (t, J = 7.2 Hz, 6H).

^{13}C{^{1}H} NMR (101 MHz, CDCl$_3$) δ (ppm): 168.1, 164.7, 149.3, 147.4, 133.7, 133.5, 130.3, 128.7, 127.8, 126.3, 125.2, 124.3, 118.7, 116.2, 80.4, 63.4, 21.1, 13.7.

HRMS (ESI) *m/z* calcd for $C_{24}H_{23}N_2O_6S$ [M+H]$^+$: 467.1277; found: 467.1271

Diethyl 2-acetoxy-2-(2-phenylimidazo[1,2-*a*]pyrimidin-3-yl)malonate (4w):

Yield: 85% (70 mg).

Nature: white solid

Mp: 119-121 $^{\circ}$C

R_f value = 0.3 [EtOAc: Petroleum ether = 6:4 (v/v)].

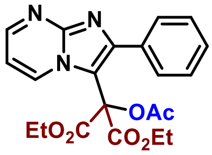

4w

^{1}H NMR (400 MHz, CDCl$_3$) δ (ppm): 8.66 (dt, J = 4.8, 1.9 Hz, 2H), 7.64 (dd, J = 7.5, 1.9 Hz, 2H), 7.42 (dd, J = 4.9, 2.5 Hz, 3H), 6.96 (dd, J = 7.0, 4.1 Hz, 1H), 3.94 (q, J = 7.2 Hz, 4H), 2.09 (s, 3H), 1.14 (t, J = 7.1 Hz, 6H).

^{13}C{^{1}H} NMR (101 MHz, CDCl$_3$) δ (ppm): 168.8, 163.9, 151.2, 148.1, 134.9, 133.4, 130.4, 128.9, 127.9, 112.1, 109.0, 79.7, 63.5, 20.4, 13.8.

HRMS (ESI) *m/z* calcd for $C_{21}H_{21}N_3NaO_6$ [M+Na]$^+$: 434.1328; found: 434.1319

Ethyl 2-acetoxy-2-cyano-2-(2-phenylimidazo[1,2-*a*]pyridin-3-yl)acetate (4x):

Yield: 48% (35 mg).

Nature: yellow gummy liquid

R_f value = 0.2 [EtOAc: Petroleum ether = 3:7 (v/v)].

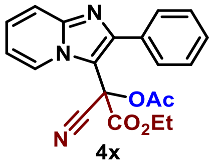

4x

^{1}H NMR (400 MHz, CDCl$_3$) δ (ppm): 8.53 (dd, J = 6.1, 0.9 Hz, 1H), 7.73 (d, J = 9.1 Hz, 1H), 7.55 – 7.53 (m, 2H), 7.44 – 7.39 (m, 4H), 7.03 (td, J = 7.0, 1.1 Hz, 1H), 3.98 (dq, J = 10.6, 7.1 Hz, 1H), 3.75 (dq, J = 10.7, 7.1 Hz, 1H), 2.15 (s, 3H), 1.21 (t, J = 7.1 Hz, 3H).

^{13}C{^{1}H} NMR (101 MHz, CDCl$_3$) δ (ppm): 168.5, 161.1, 147.8, 146.1, 133.4, 130.4, 128.9, 127.7, 126.7, 125.9, 118.2, 113.6, 112.5, 108.3, 83.3, 64.5, 20.1, 13.6.

HRMS (ESI) *m/z* calcd for $C_{20}H_{18}N_3O_4$ [M+H]$^+$: 364.1297; found: 364.1293

Ethyl 2-acetoxy-3-oxo-2-(2-phenylimidazo[1,2-*a*]pyridin-3-yl)butanoate (4y):

Yield: 83% (63 mg).

Nature: white solid

Mp: 114-116 °C

R_f value = 0.3 [EtOAc: Petroleum ether = 4:6 (v/v)]

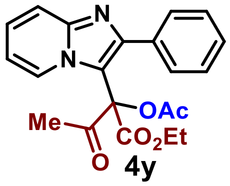

4y

^{1}H NMR (400 MHz, CDCl$_3$) δ (ppm): 8.13 (d, J = 7.0 Hz, 1H), 7.66 (dd, J = 5.6, 4.5 Hz, 1H), 7.52 – 7.49 (m, 2H), 7.43 (dd, J = 5.1, 2.2 Hz, 3H), 7.32 (ddd, J = 8.8, 6.8, 1.1 Hz, 1H), 6.88 (td, J = 6.9, 1.2 Hz, 1H), 3.65 (dd, J = 10.7, 7.2 Hz, 1H), 3.39 (dd, J = 10.7, 7.2 Hz, 1H), 2.34 (s, 3H), 2.17 (s, 3H), 1.02 (t, J = 7.1 Hz, 3H).

^{13}C{^{1}H} NMR (101 MHz, CDCl$_3$) δ (ppm): 196.3, 169.1, 164.2, 147.2, 145.8, 134.2, 130.3, 128.8, 127.9, 126.6, 126.3, 117.9, 113.3, 111.6, 85.2, 62.6, 27.3, 20.5, 13.5.

HRMS (ESI) *m/z* calcd for $C_{21}H_{21}N_2O_5$ $[M+H]^+$: 381.1450; found: 381.1452

Diethyl 2-(2-phenylimidazo[1,2-*a*]pyridin-3-yl)malonate (5):[6]

Yield: 66% (46 mg).

Nature: yellow oil

R_f value = 0.3 [EtOAc: Petroleum ether = 3:7 (v/v)].

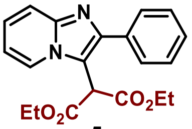

5

^{1}H NMR (400 MHz, CDCl$_3$) δ (ppm): 8.35 – 8.33 (m, 1H), 7.76 (dd, J = 8.3, 1.3 Hz, 2H), 7.68 – 7.65 (m, 1H), 7.51 – 7.47 (m, 2H), 7.41 (dt, J = 9.4, 4.4 Hz, 1H), 7.25 – 7.22 (m, 1H), 6.82 (td, J = 6.9, 1.2 Hz, 1H), 5.40 (s, 1H), 4.23 (qd, J = 7.2, 3.1 Hz, 4H), 1.24 (t, J = 7.2 Hz, 6H).

^{13}C{^{1}H} NMR (101 MHz, CDCl$_3$) δ (ppm): 166.8, 146.4, 145.9, 133.9, 129.2, 128.8, 128.3, 126.3, 125.3, 117.7, 112.1, 111.9, 62.5, 49.3, 14.1.

5. Mechanistic studies and control experiments:

5.1. Radical trapping with TEMPO: An oven-dried culture tube equipped with a magnetic stirring bar was charged with 2-phenylimidazo[1,2-*a*]pyridine (**1a**, 39 mg, 0.2 mmol, 1.0 equiv), diethyl bromomalonate (**2a**, 96 mg, 0.4 mmol, 2.0 equiv), $Zn(OAc)_2$ (132 mg, 0.6 mmol, 3.0 equiv), photocatalyst **PTH** (5 mol %) in dry 1,2-DCE (2 mL) followed by the addition of TEMPO (94 mg, 0.6 mmol, 3.0 equiv). The resulting reaction mixture was stirred at room temperature under an open atmosphere using 390 nm violet LED for 10 h. A fan cooling was also included to maintain the reaction at room temperature. After the reaction, it was found that the formation of acetoxymalonylated product **4a** was suppressed significantly, and the TEMPO-DEM adduct **7** and TEMPO-OAc adduct **8** were detected by HRMS analysis of the crude reaction mixture, indicating the involvement of a malonyl radical and acetyl radical during the reaction.

[6] Huang M.; Wang L.; Yang X.; Kim J. K.; Gong M.; Zhang J.; Li Y.; Wu Y. *Tetrahedron.* **2022**, *126*, 132988.

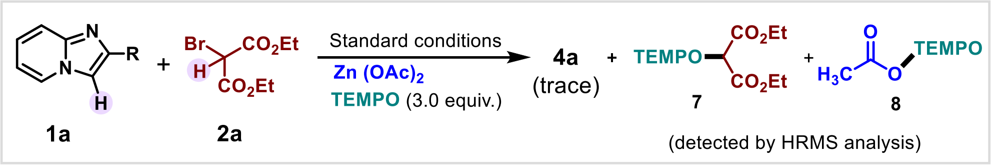

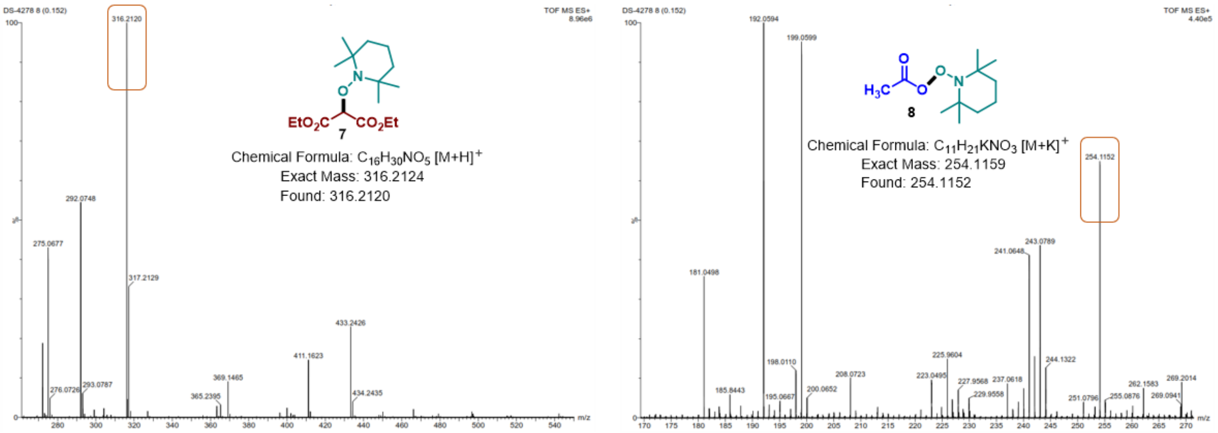

Figure S1: HRMS spectrum of the crude reaction mixture (compounds **7** and **8**).

5.2. Radical trapping with alkene: An oven-dried culture tube equipped with a magnetic stirring bar was charged with 2-phenylimidazo[1,2-*a*]pyridine (**1a**, 39 mg, 0.2 mmol, 1.0 equiv), diethyl bromomalonate (**2a**, 96 mg, 0.4 mmol, 2.0 equiv), 5-hexene-1-ol (47 µL, 0.4 mmol, 2.0 equiv), photocatalyst **PTH** (5 mol %) in dry 1,2-DCE (2 mL). The resulting reaction mixture was stirred at room temperature under an open atmosphere using 390 nm violet LED for 15 h. A fan cooling was also included to maintain the reaction at room temperature. After the reaction, ATRA product **9** was isolated via column chromatography. This further confirms the involvement of a malonyl radical during the reaction.

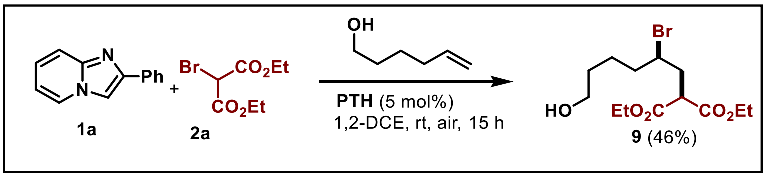

**Diethyl 2-(2-bromo-6-hydroxyhexyl)malonate (9):**[7]	
**Yield:** 46% (31 mg).<br>**Nature:** colourless oil.<br>**Rf value** = 0.3 [EtOAc: Petroleum ether = 3:7 (v/v)].	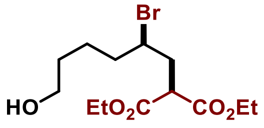<br>9

[7] Nguyen, J. D.; Tucker, J. W.; Konieczynska, M. D.; Stephenson, C. R. J. *J. Am. Chem. Soc.* **2011**, *133*, 4160−4163.

^{1}H NMR (400 MHz, CDCl$_3$) *δ* (ppm): 4.25 – 4.14 (m, 4H), 4.04 – 3.94 (m, 1H), 3.76 (dd, *J* = 10.2, 4.2 Hz, 1H), 3.63 (t, *J* = 6.1 Hz, 2H), 2.45 (ddd, *J* = 14.7, 10.2, 3.1 Hz, 1H), 2.23 (ddd, *J* = 14.8, 10.6, 4.2 Hz, 1H), 1.90 – 1.84 (m, 2H), 1.66 – 1.47 (m, 4H), 1.26 (td, *J* = 7.1, 3.8 Hz, 6H).

^{13}C{^{1}H} NMR (101 MHz, CDCl$_3$) *δ* (ppm): 169.1, 168.9, 62.6, 61.8, 61.7, 54.8, 50.7, 39.2, 37.9, 32.0, 23.8, 14.15, 14.11.

5.3. Reaction of 2-phenylimidazo[1,2-*a*]pyridine and bromomalonate without Zn(OAc)$_2$:

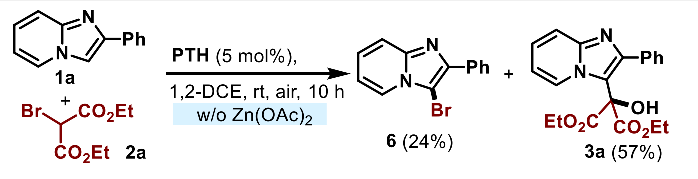

An oven-dried culture tube equipped with a magnetic stirring bar was charged with 2-phenylimidazo[1,2-*a*]pyridine (**1a**, 39 mg, 0.2 mmol, 1.0 equiv), diethyl bromomalonate (**2a**, 96 mg, 0.4 mmol, 2.0 equiv) and photocatalyst **PTH** (5 mol %) in dry 1,2-DCE (2 mL). The resulting reaction mixture was stirred at room temperature under open atmosphere in using 390 nm violet LED for 10 h. A fan cooling was also included to maintain the reaction at room temperature. The completion of the reaction was confirmed by TLC. Then, the crude reaction mixture was poured into H_2O (10 mL) and extracted with CH_2Cl_2 (3 × 6 mL). The combined organic layer was washed with brine solution and dried using oven dried anhydrous Na_2SO_4. Then, the mixture was concentrated in a rotary evaporator under reduced pressure and the crude residue was purified by column chromatography to obtain the products **3a** and **6**.

**Diethyl 2-hydroxy-2-(2-phenylimidazo[1,2-*a*]pyridin-3-yl)malonate (3a):**[6]	
**Yield:** 57% (42 mg).<br>**Nature:** white solid<br>**Mpt:** 147-149 °C<br>**R$_f$ value** = 0.2 [EtOAc: Petroleum ether = 4:6 (v/v)].	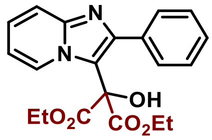<br>**3a**
**^{1}H NMR (400 MHz, CDCl$_3$) *δ* (ppm):** 8.08 – 8.06 (m, 1H), 7.67 – 7.65 (m, 1H), 7.53 (dd, *J* = 8.2, 1.5 Hz, 2H), 7.41 – 7.34 (m, 3H), 7.25 (q, *J* = 5.7 Hz, 1H), 6.81 (td, *J* = 6.9, 1.2 Hz, 1H), 4.68 (s, 1H), 3.94 (dq, *J* = 10.6, 7.0 Hz, 2H), 3.67 (dq, *J* = 10.6, 7.1 Hz, 2H), 1.02 (t, *J* = 7.1 Hz, 6H).<br>**^{13}C{^{1}H} NMR (101 MHz, CDCl$_3$) *δ* (ppm):** 168.7, 146.2, 145.2, 135.0, 129.8, 128.3, 128.1, 125.7, 125.5, 117.9, 115.6, 112.6, 76.9, 63.5, 13.7.	

3-Bromo-2-phenylimidazo[1,2-*a*]pyridine (6)[8]**:**

Yield: 24% (13 mg).

Nature: yellow liquid

$\mathbf{R_f}$ **value** = 0.6 [EtOAc: Petroleum ether = 4:6 (v/v)].

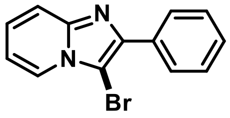

6

^{1}H NMR (400 MHz, $CDCl_3$) δ (ppm): 8.16 – 8.12 (m, 3H), 7.63 (dd, *J* = 10.1, 0.9 Hz, 1H), 7.49 (dd, *J* = 10.4, 4.6 Hz, 2H), 7.41 – 7.37 (m, 1H), 7.24 – 7.22 (m, 1H), 6.91 (td, *J* = 6.8, 0.9 Hz, 1H).

^{13}C{^{1}H} NMR (101 MHz, $CDCl_3$) δ (ppm): 145.5, 142.7, 132.9, 128.6, 128.4, 127.9, 125.2, 124.0, 117.7, 113.1, 91.8.

5.4. Competitive acylation reaction with benzoic acid

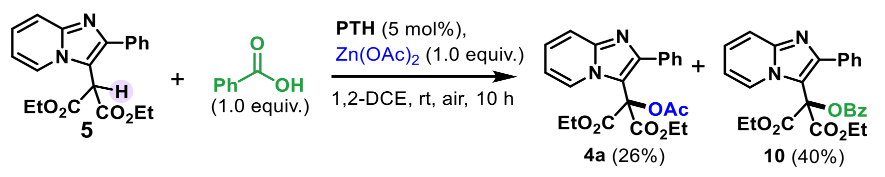

An oven-dried culture tube equipped with a magnetic stirring bar was charged with diethyl 2-(2-phenylimidazo[1,2-*a*]pyridin-3-yl)malonate (**5**, 70 mg, 0.2 mmol, 1.0 equiv), benzoic acid (24 mg, 0.2 mmol, 1.0 equiv), $Zn(OAc)_2$ (44 mg, 0.2 mmol, 1.0 equiv) and photocatalyst **PTH** (5 mol %). The resulting reaction mixture was stirred at rt under open atmosphere condition using 390 nm violet LED for 10 h. The completion of the reaction was confirmed by TLC. Then, the crude reaction mixture was poured into H_2O (10 mL) and extracted with CH_2Cl_2 (3 × 6 mL). The combined organic layer was washed with brine solution and dried using oven dried anhydrous Na_2SO_4. Then, the mixture was concentrated in a rotary evaporator under reduced pressure and the crude residue was purified by column chromatography to obtain the desired products **4a** and **10**.

Diethyl 2-(benzoyloxy)-2-(2-phenylimidazo[1,2-*a*]pyridin-3-yl)malonate (10):

Yield: 40% (38 mg).

Nature: yellow gummy liquid

$\mathbf{R_f}$ **value** = 0.3 [EtOAc: Petroleum ether = 4:6 (v/v)].

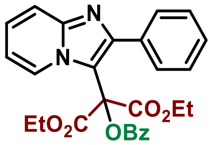

10

^{1}H NMR (400 MHz, $CDCl_3$) δ (ppm): 8.43 (dd, *J* = 8.1, 0.9 Hz, 1H), 7.92 (dd, *J* = 8.4, 1.1 Hz, 2H), 7.73 (d, *J* = 8.9 Hz, 1H), 7.64 (dd, *J* = 7.9, 1.6 Hz, 2H), 7.60 – 7.56 (m, 1H), 7.43 – 7.39 (m, 5H), 7.33 (ddd, *J* = 9.1, 6.8, 1.1 Hz, 1H), 6.90 (tt, *J* = 12.9, 6.5 Hz, 1H), 3.98 – 3.86 (m, 4H), 1.13 (t, *J* = 7.1 Hz, 6H).

^{13}C{^{1}H} NMR (101 MHz, $CDCl_3$) δ (ppm): 164.4**,** 164.3, 146.8, 145.5, 134.3, 133.9, 130.3, 130.2, 128.9, 128.7, 128.6, 127.9, 126.4, 125.8, 118.2, 113.7, 113.1, 80.4, 63.3, 13.8.

HRMS (ESI) *m/z* calcd for $C_{27}H_{25}N_2O_6$ $[M+H]^+$: 473.1713; found: 473.1711.

[8] Semwal R.; Ravi C.; Kumar R.; Meena R.; Adimurthy S. *J. Org. Chem.* **2018**, *84*, 792.

5.5. Stern–Volmer fluorescence quenching experiments

All emission spectra were recorded using a Shimadzu RF-6000 Spectrophotometer (model no.-A40246002251SA). Photocatalyst **PTH** and different concentrations of added quenchers were prepared in dry and degassed acetonitrile in quartz cuvettes. For the quenching experiments, the concentration of **PTH** was 4.0×10^{-5} M. The solutions were excited at 320 nm, and the emission intensity was measured at 445 nm for **PTH**. Plots were derived according to the Stern–Volmer equation, and K_{sv} was calculated.

$$\text{Stern–Volmer equation:}\quad I_0/I = 1 + K_{sv}[Q]$$

Where I_0 is the luminescence intensity of the photocatalyst in the absence of a quencher, I is the intensity of the photocatalyst in the presence of quenchers, [Q] is the concentration of added quencher, and K_{sv} is the Stern–Volmer quenching constant. All emission spectra were recorded after each addition of the quencher. The obtained spectra (Figure S2) show that diethyl bromomalonate (**2a**) is the prominent quencher here and suggested a mechanism started with radical engagement of **2a**.

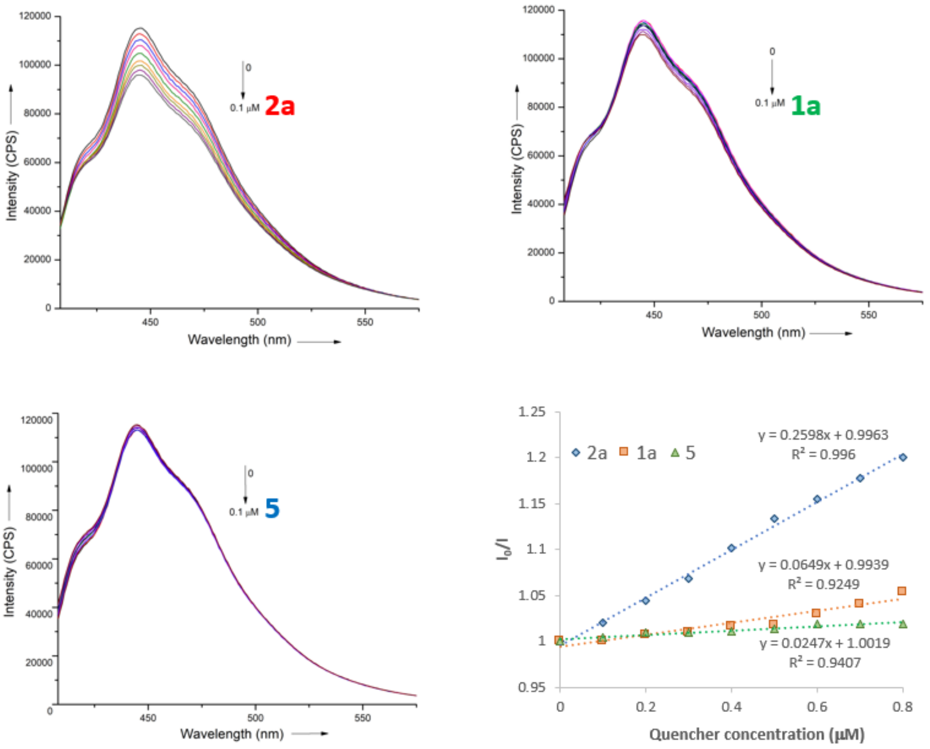

Figure S2: The fluorescence emission spectra of PTH with different concentrations of added quenchers diethyl bromo malonates (**2a**), 2-phenylimidazo[1,2-*a*]pyridine (**1a**), and diethyl 2-(2-phenylimidazo[1,2-*a*]pyridin-3-yl)malonate (**5**).

5.6. Analysis of the aqueous extract of the crude reaction mixture

After completion of the reaction, the crude reaction mixture was extracted with CH_2Cl_2 (3 × 6 mL) and water (10 mL). The water extract was evaporated on a rotary evaporator under reduced pressure and then dried by a high vacuum pump with temperature to get an off-white solid. The solid material is highly hygroscopic and liquified upon exposure to air. HRMS spectra of the material clearly indicated that it is nothing but the zinc bromide ($ZnBr_2$). In addition, the aqueous part of crude reaction mixture has vinegar-like smell, which indicates in situ generation of acetic acid, which was further confirmed by GC–MS analysis.

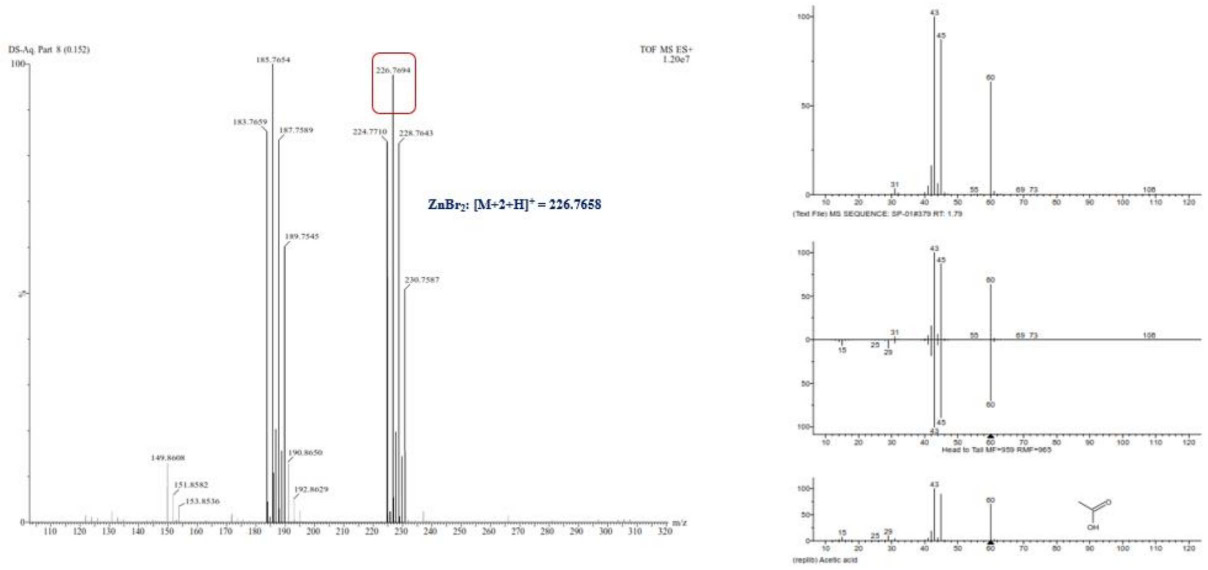

Figure S3: HRMS and GC–MS spectra of the aqueous extract of the crude reaction mixture.

5.7. NMR experiment

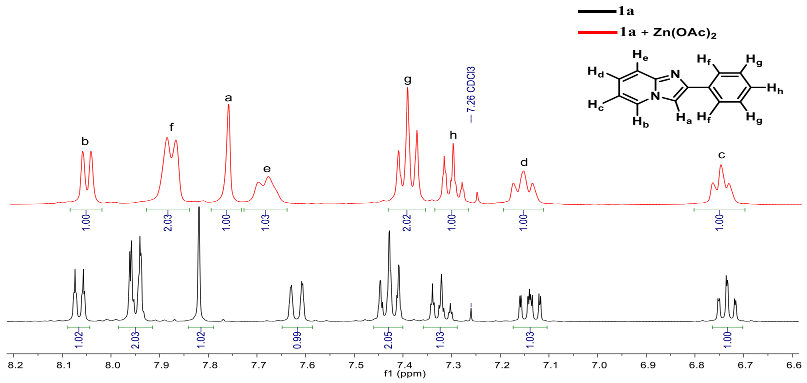

Figure S4: ^{1}H NMR spectra were taken in $CDCl_3$: 2-phenylimidazo[1,2-*a*]pyridine (**1a**; black); after interaction of **1a** with $Zn(OAc)_2$ (red), in the absence of **PTH.** δ_H = 7.26 ppm represents the residual solvent signal in $CDCl_3$.

General procedure for the NMR experiment: To check if $Zn(OAc)_2$ interacts with the 2-phenylimidazo[1,2-*a*]pyridine moiety individual ^{1}H NMR spectra of 2-phenylimidazo[1,2-*a*]pyridine and 2-phenylimidazo[1,2-*a*]pyridine with $Zn(OAc)_2$ [**1**:**1**] in $CDCl_3$ were recorded. Observations from this study revealed the shifting of peaks in the aromatic region, more specifically the signal for the C-$\mathbf{H_e}$ proton was shifted towards the downfield region as clearly indicated above in Figure S4. This is possibly due to coordination type interaction of Zn with the N-atom at C-1 position, which facilitates the reaction process to uplift the overall reaction yield via preactivation of the IP unit.

6. X-ray crystal data with ORTEP plot for compound 4d.

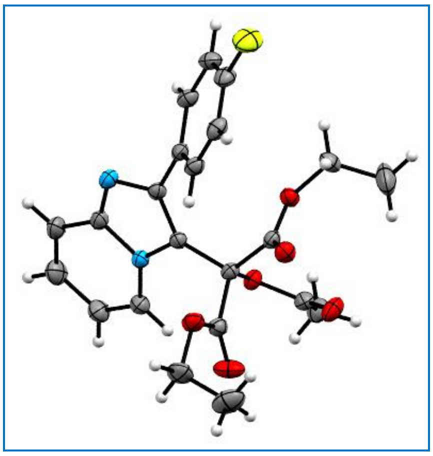

Figure S5: ORTEP plot of compound **4d** with 30% ellipsoid probability.

Crystal data for 4d: X-ray single crystal data were collected using MoKα (λ = 0.71073 Å) radiation on a Rigaku SuperNova diffractometer equipped with an Eos S2 detector. Structure solution/refinement were carried out using Shelx-2013. The structure was solved by direct method and refined in a routine manner. Nonhydrogen atoms were treated anisotropically. All hydrogen atoms were geometrically fixed. CCDC (CCDC No: 2221790) contains the supplementary crystallographic data of **4d**. These data can be obtained free of charge via www.ccdc.cam.ac.uk/conts/retrieving.html (or from the Cambridge Crystallographic Data Centre, 12 Union Road, Cambridge CB21EZ, UK; fax: (+44) 1223-336-033; or deposit@ccdc.cam.ac.uk).

Table S2 Crystal data and structure refinement for compound 4d.

Identification code	CCDC 2221790
Empirical formula	$C_{22}H_{21}FN_2O_6$
Formula weight	428.41
Temperature/K	293(2)
Crystal system	monoclinic
Space group	P 21/n

a/Å	12.7400(7)
b/Å	12.9269(8)
c/Å	12.9616(7)
α/°	90
β/°	102.603(5)
γ/°	90
Volume/$Å^3$	2083.2(2)
Z	4
ρ_{calc}g/cm^3	1.366
μ/mm^{-1}	0.106
F(000)	896
Radiation	MoKα (λ = 0.71073)
Theta (min)	2.031
Theta (max)	26.999
h, k, l max	16, 16, 16
R (reflections)	0.0462 (2968)
wR2 (reflections)	0.1070 (4467)

7. NMR spectra

^{1}H NMR of **3a** (400 MHz, $CDCl_3$):

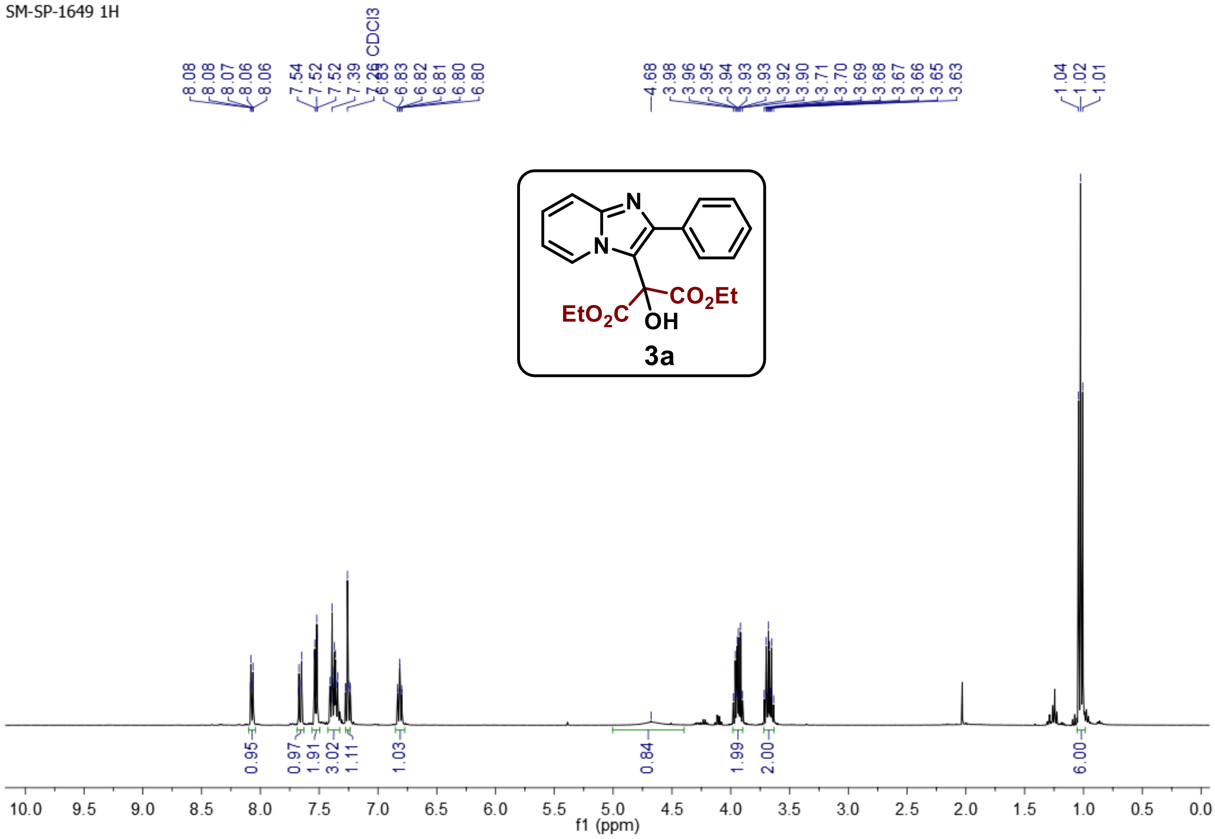

^{13}C{^{1}H} NMR of **3a** (101 MHz, $CDCl_3$):

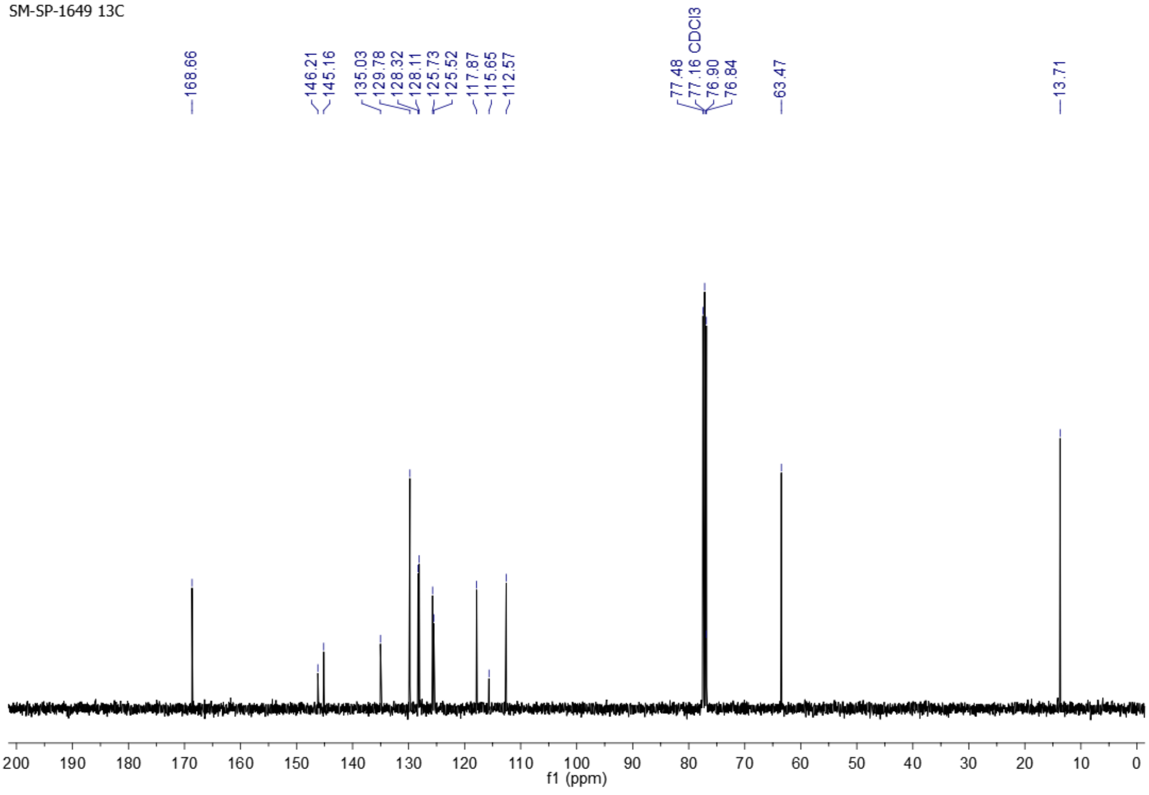

^{1}H NMR of **4a** (400 MHz, $CDCl_3$):

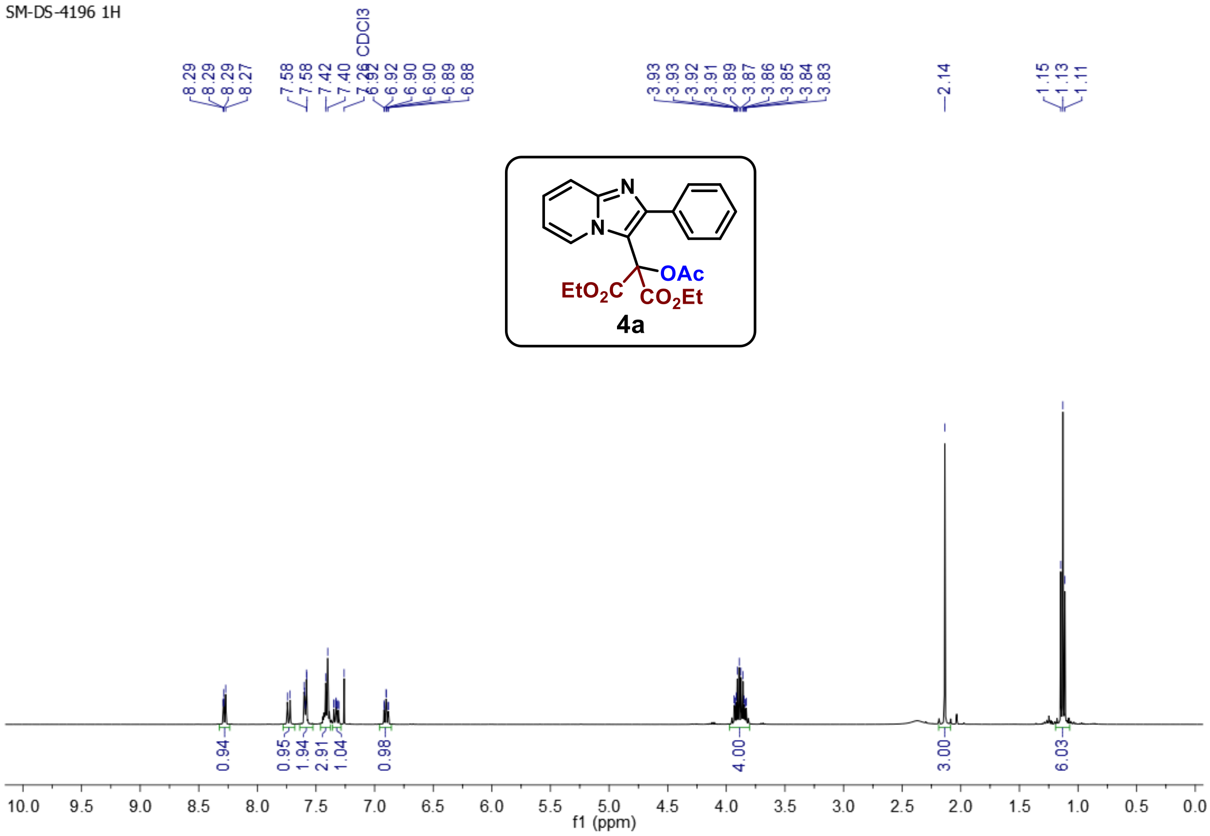

^{13}C{^{1}H} NMR of **4a** (101 MHz, $CDCl_3$):

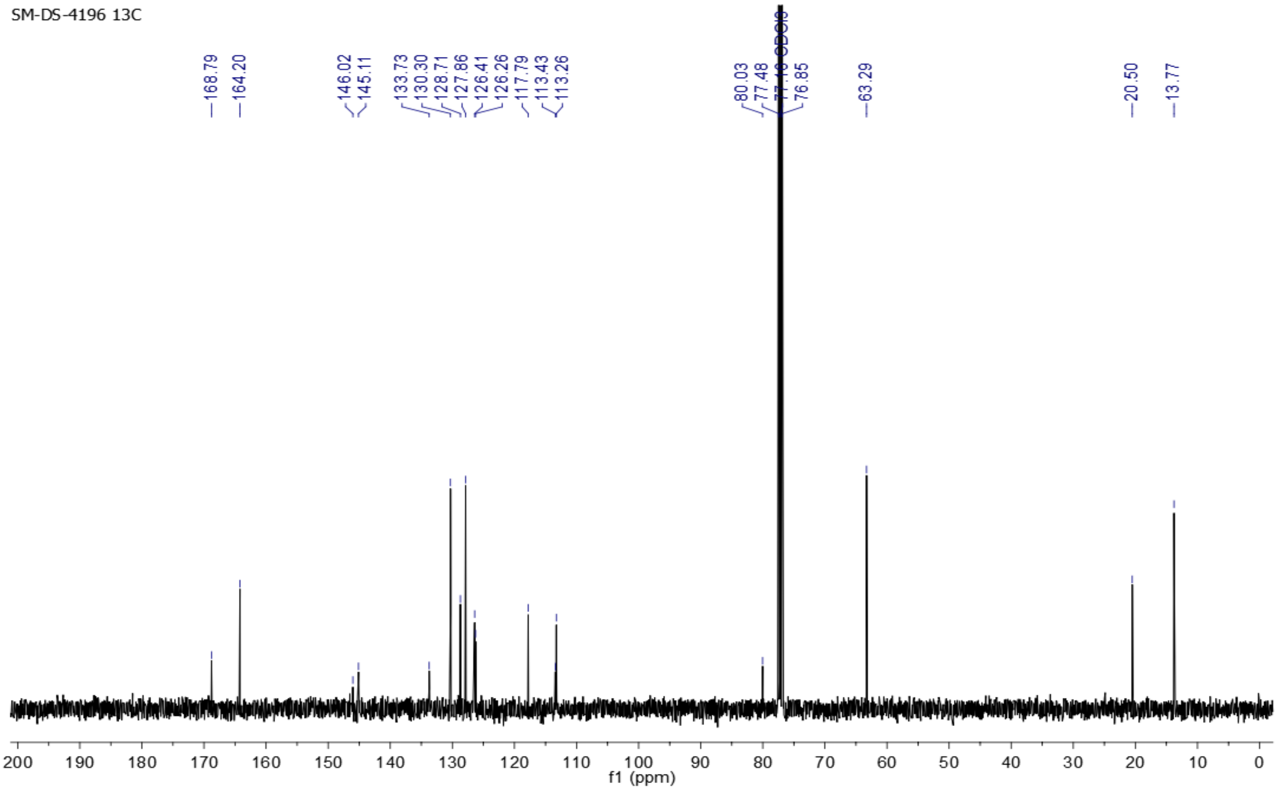

^{1}H NMR of **4b** (400 MHz, $CDCl_3$):

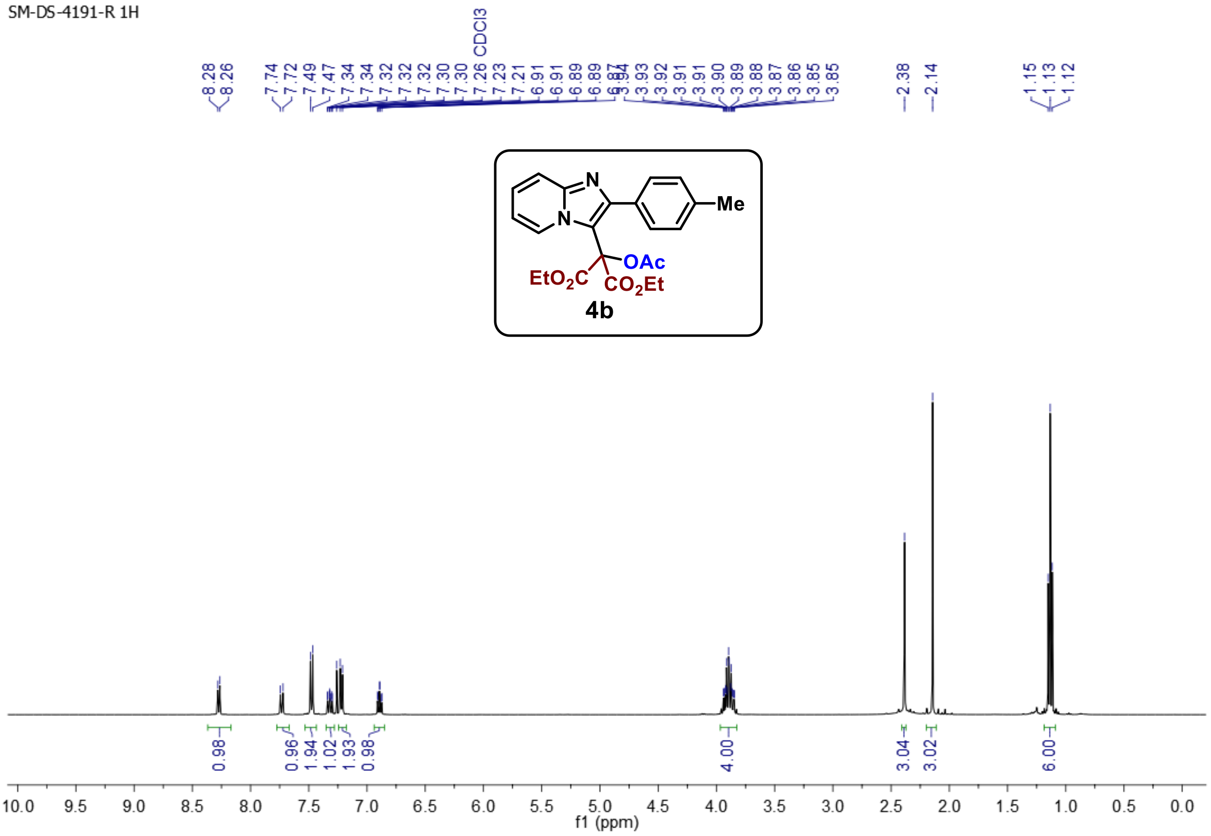

^{13}C{^{1}H} NMR of **4b** (101 MHz, $CDCl_3$):

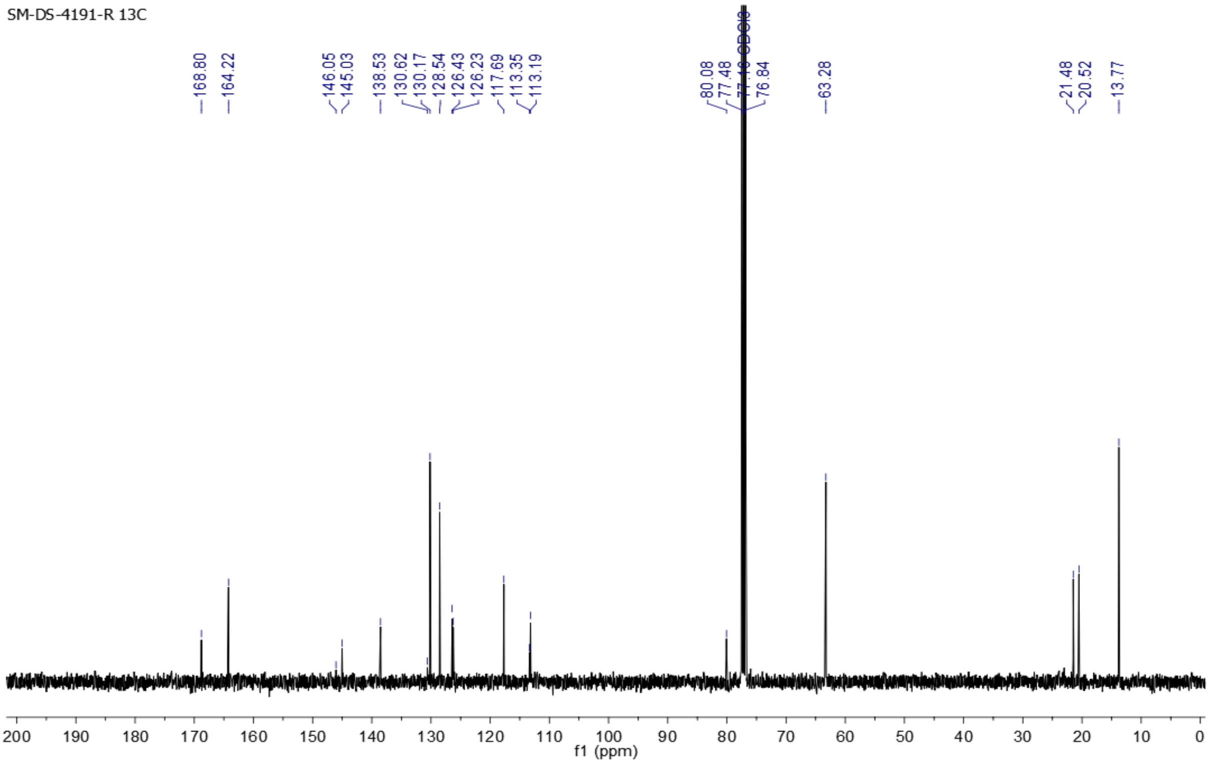

^{1}H NMR of **4c** (400 MHz, $CDCl_3$):

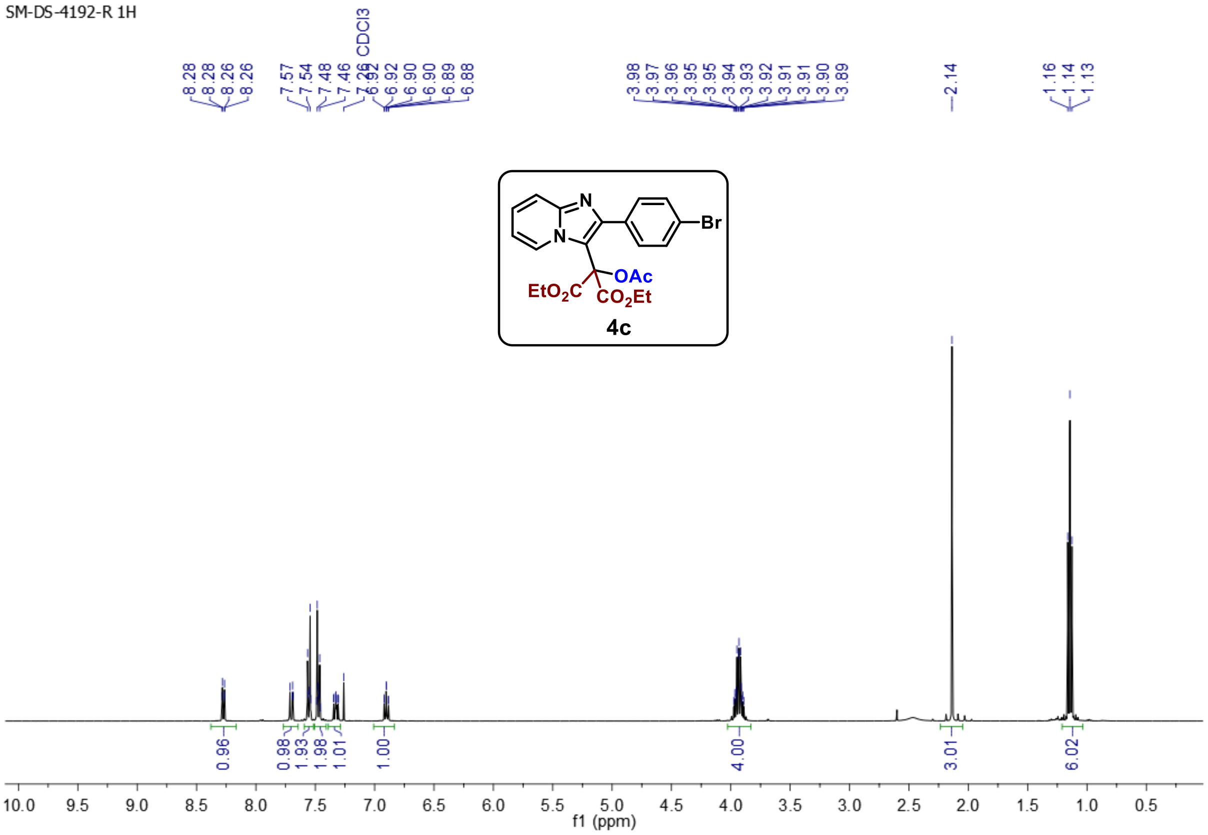

^{13}C{^{1}H} NMR of **4c** (101 MHz, $CDCl_3$):

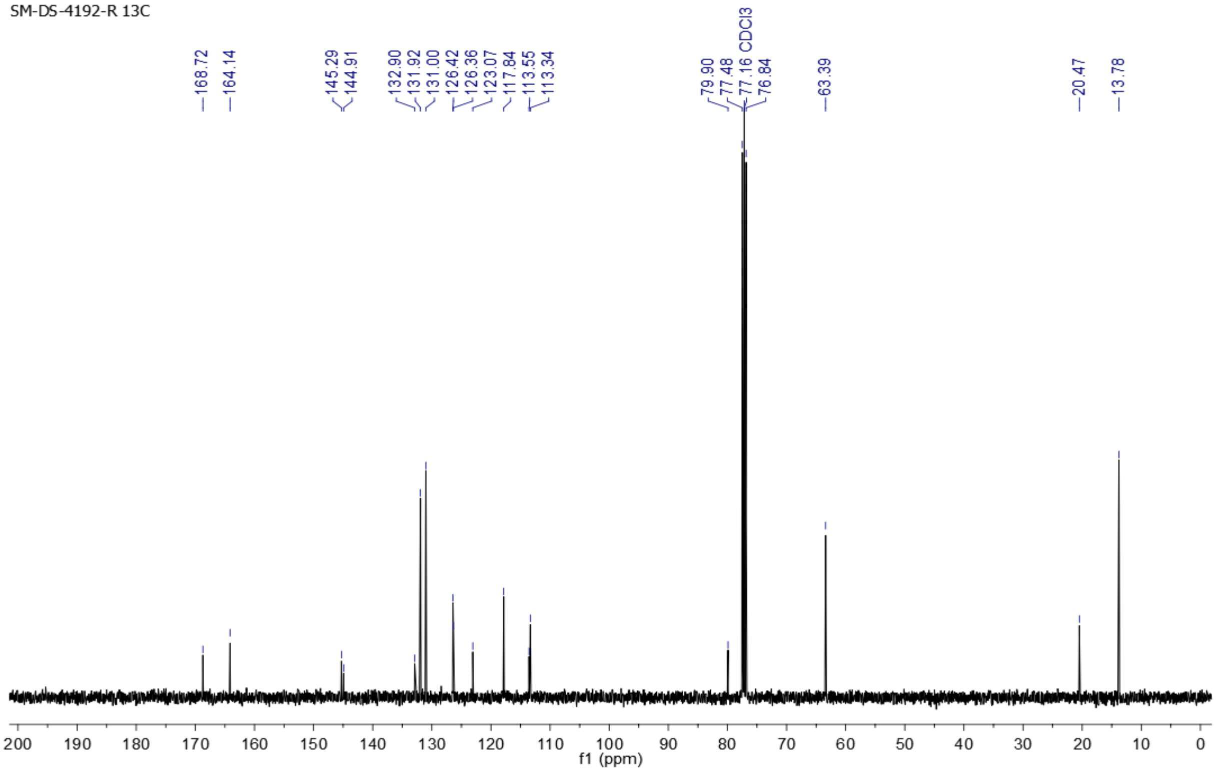

^{1}H NMR of **4d** (400 MHz, $CDCl_3$):

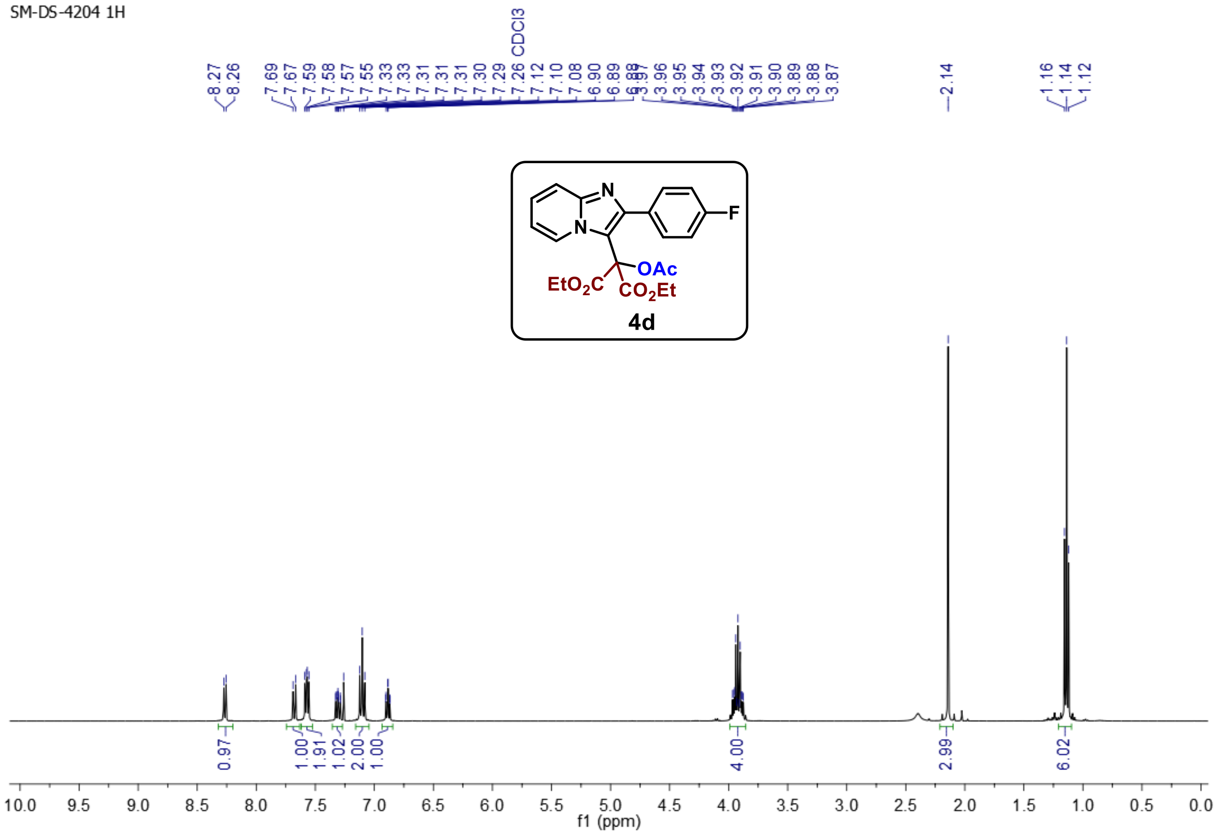

^{13}C{^{1}H} NMR of **4d** (101 MHz, $CDCl_3$):

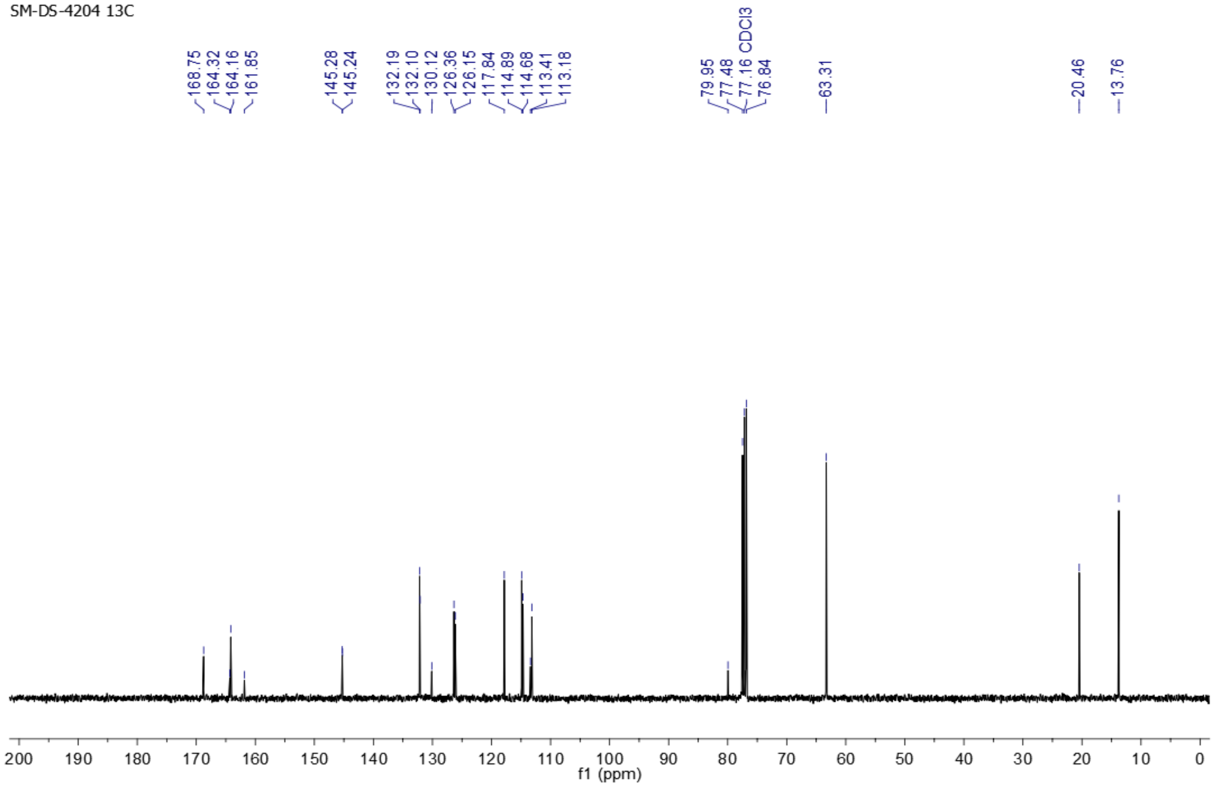

$^{19}F\{^{1}H\}$ NMR of **4d** (377 MHz, $CDCl_3$):

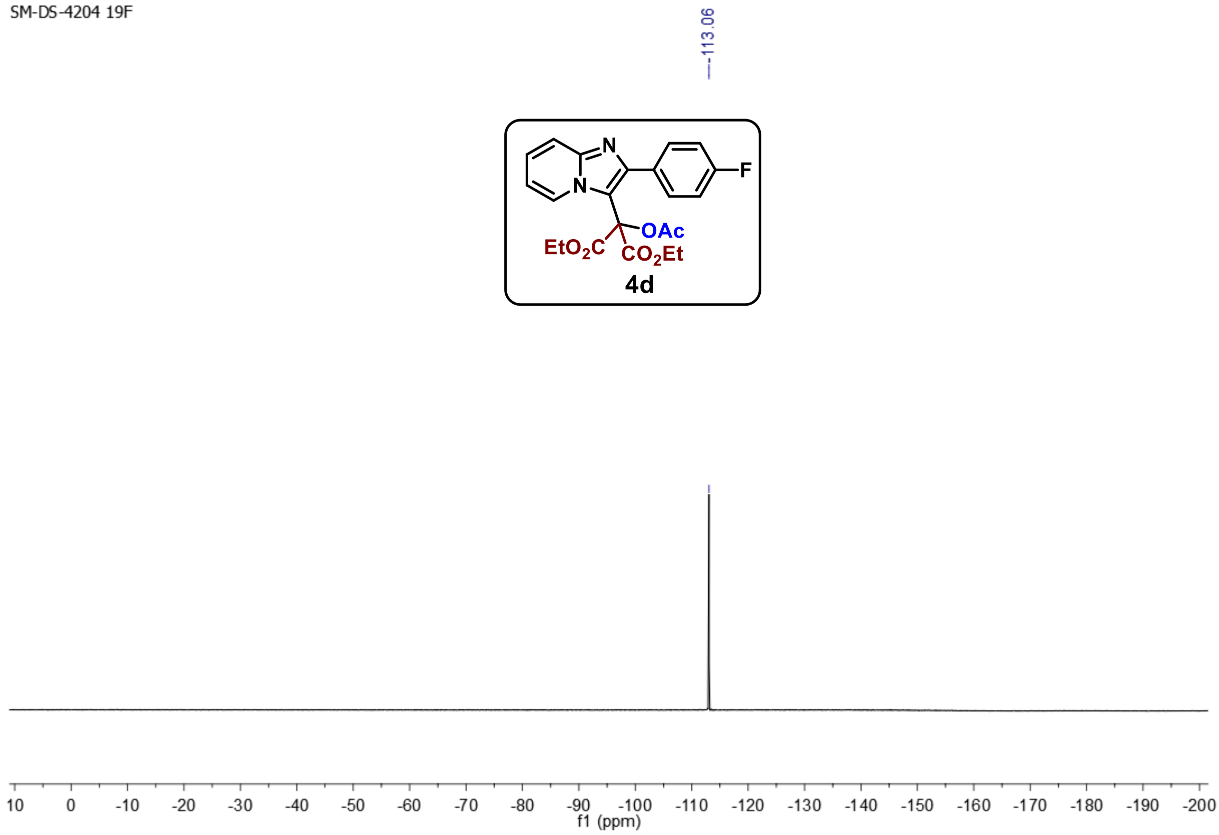

^{1}H NMR of **4e** (400 MHz, $CDCl_3$):

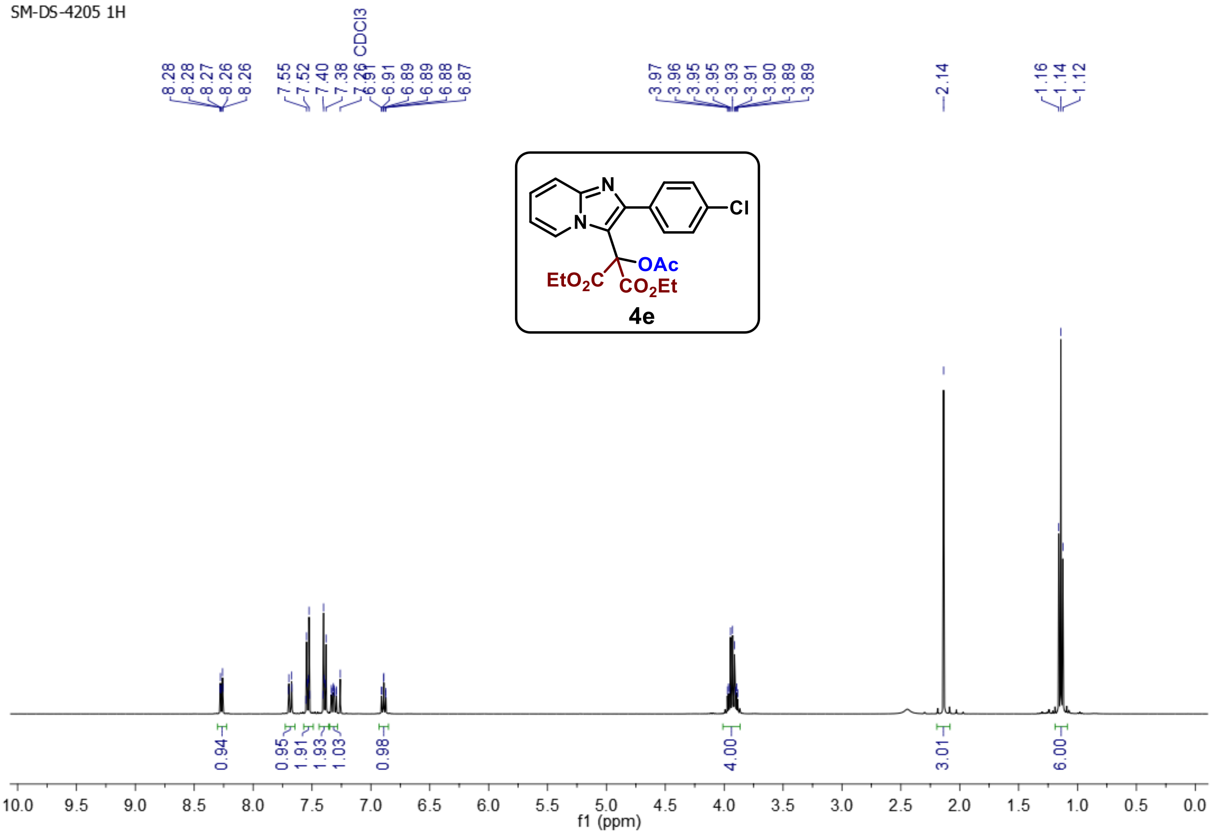

^{13}C{^{1}H} NMR of **4e** (101 MHz, $CDCl_3$):

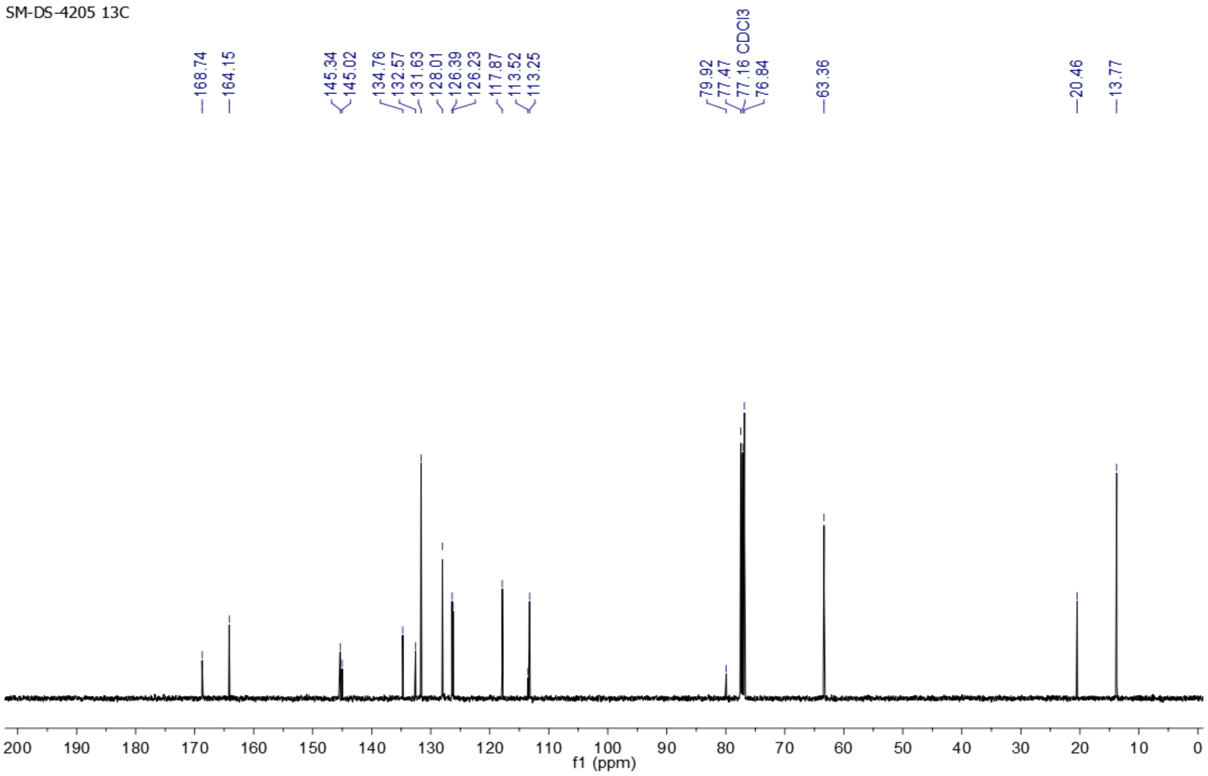

^{1}H NMR of **4f** (400 MHz, $CDCl_3$):

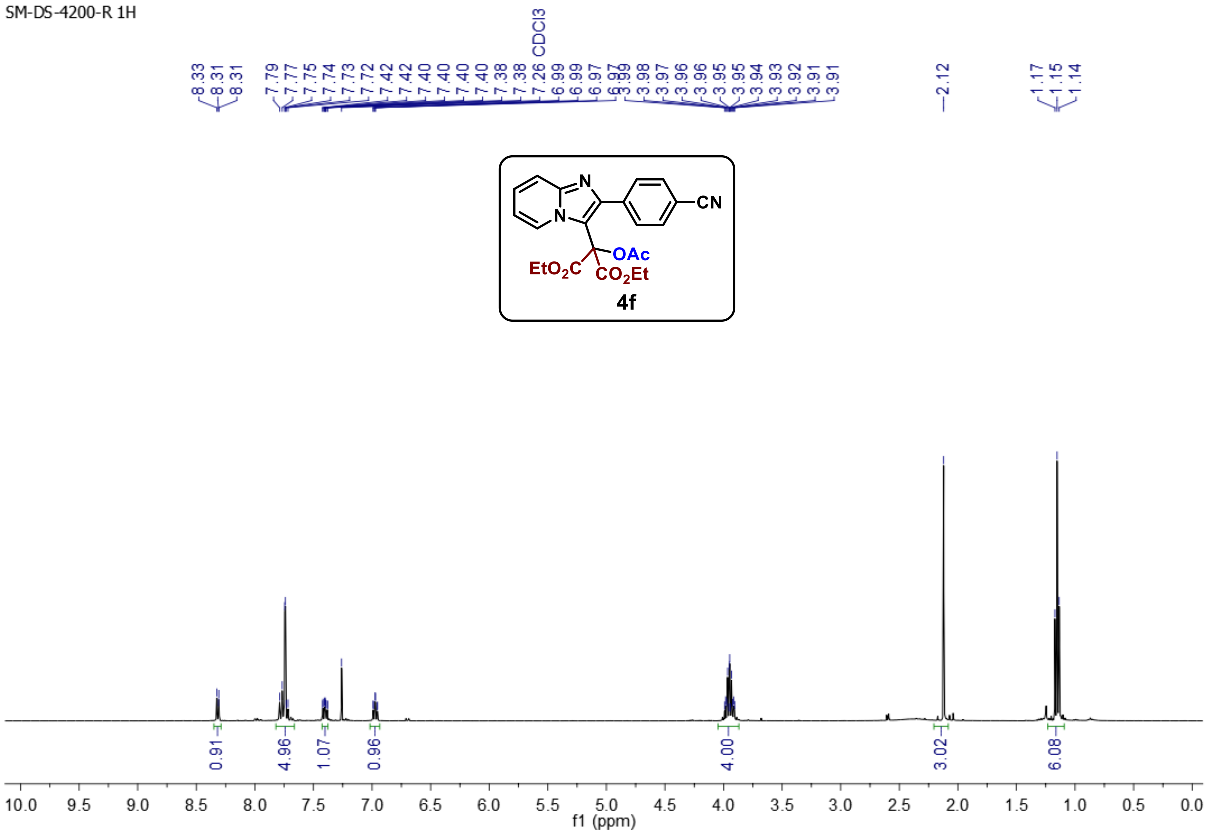

^{13}C{^{1}H} NMR of **4f** (101 MHz, $CDCl_3$):

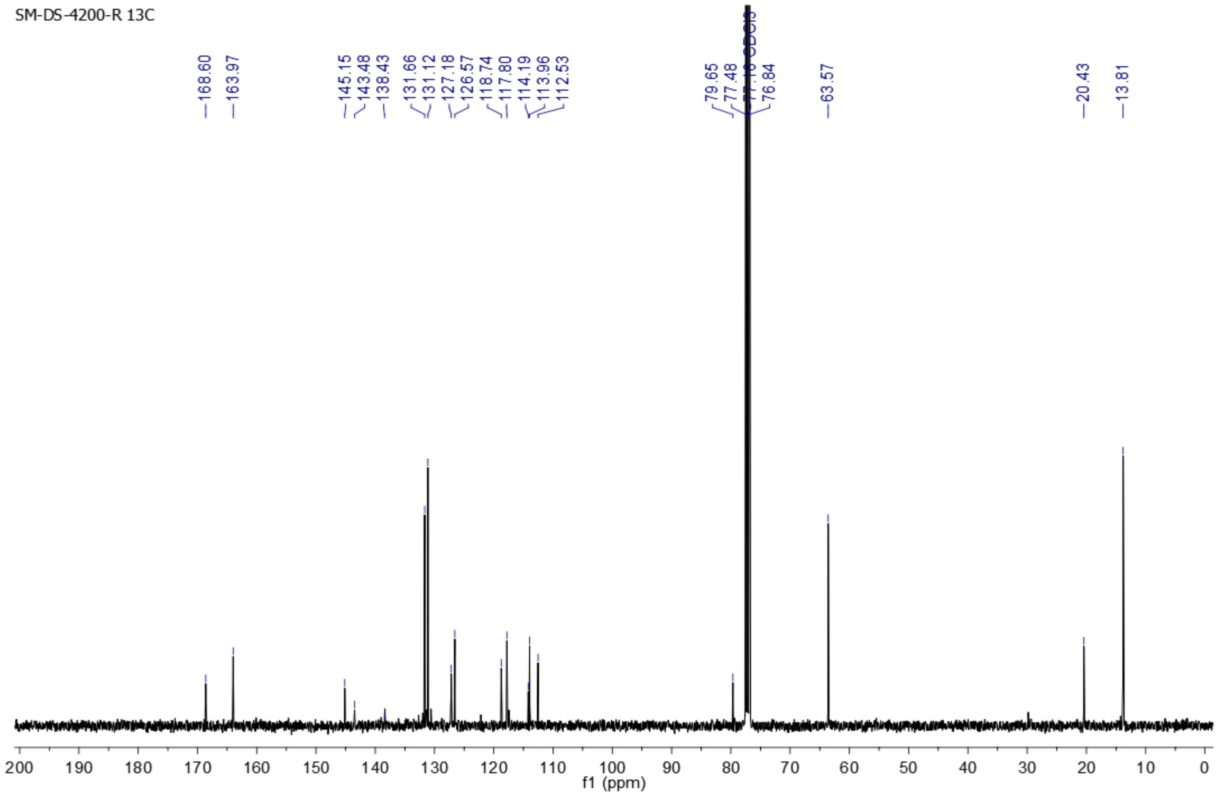

^{1}H NMR of **4g** (400 MHz, $CDCl_3$):

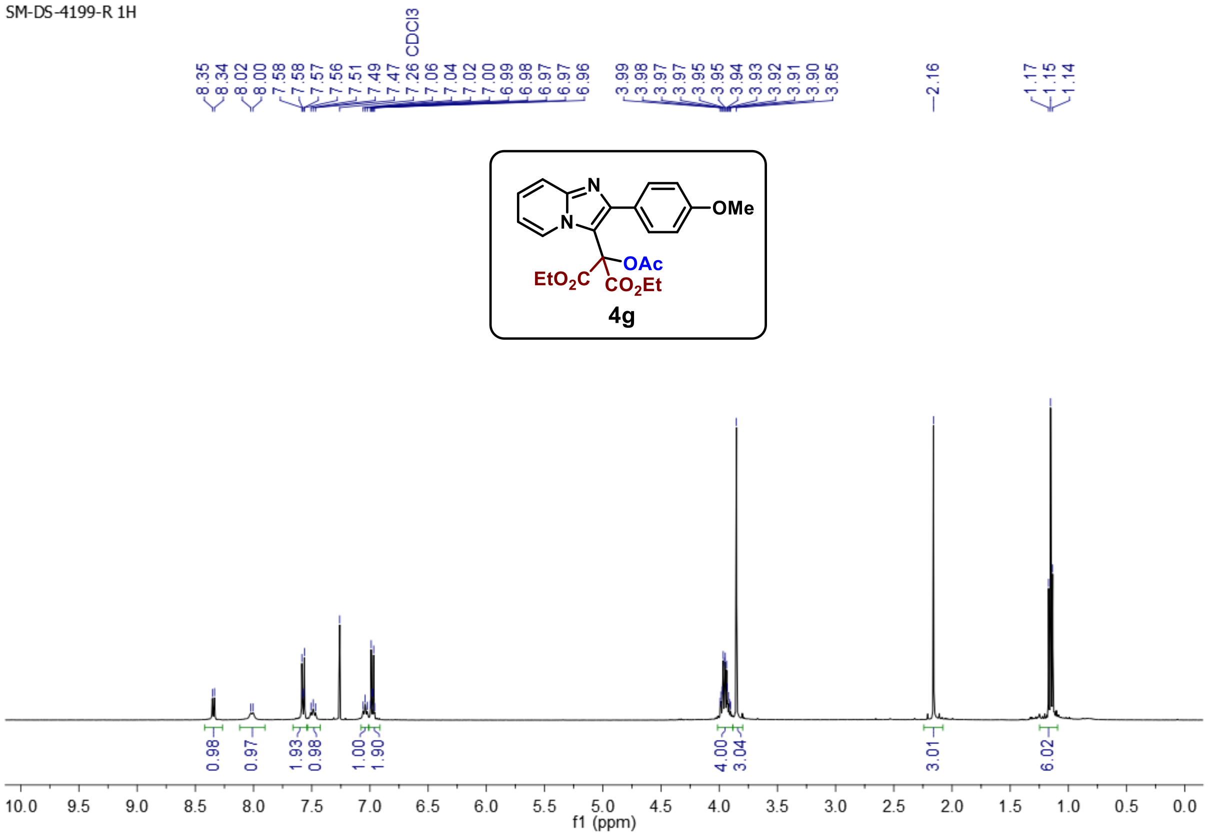

^{13}C{^{1}H} NMR of **4g** (101 MHz, $CDCl_3$):

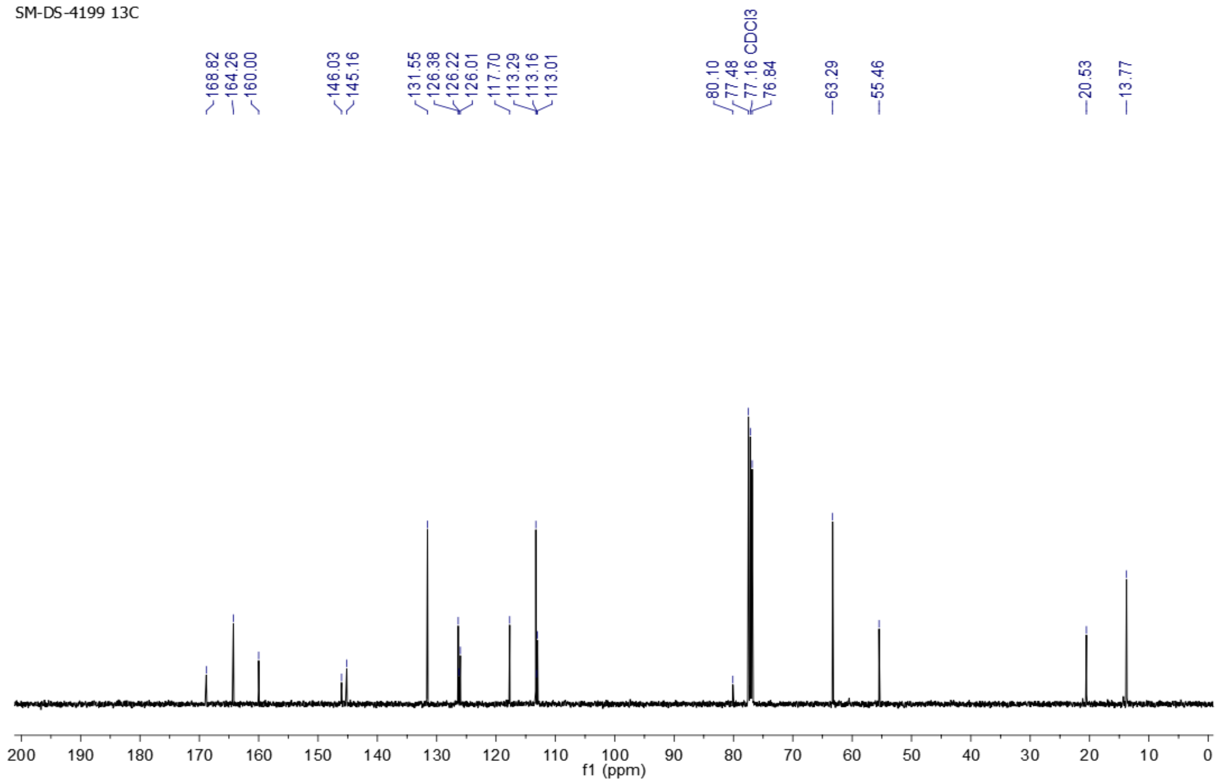

^{1}H NMR of **4h** (400 MHz, $CDCl_3$):

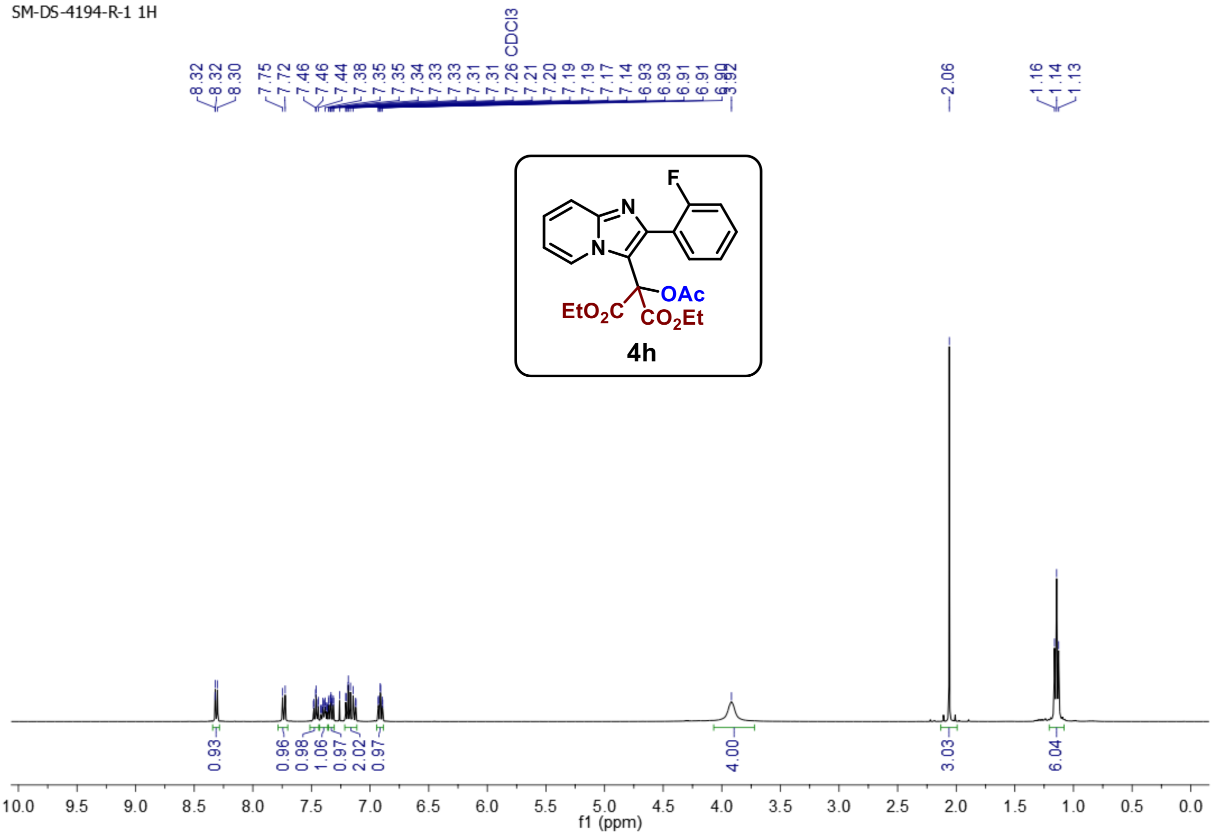

^{13}C{^{1}H} NMR of **4h** (101 MHz, $CDCl_3$):

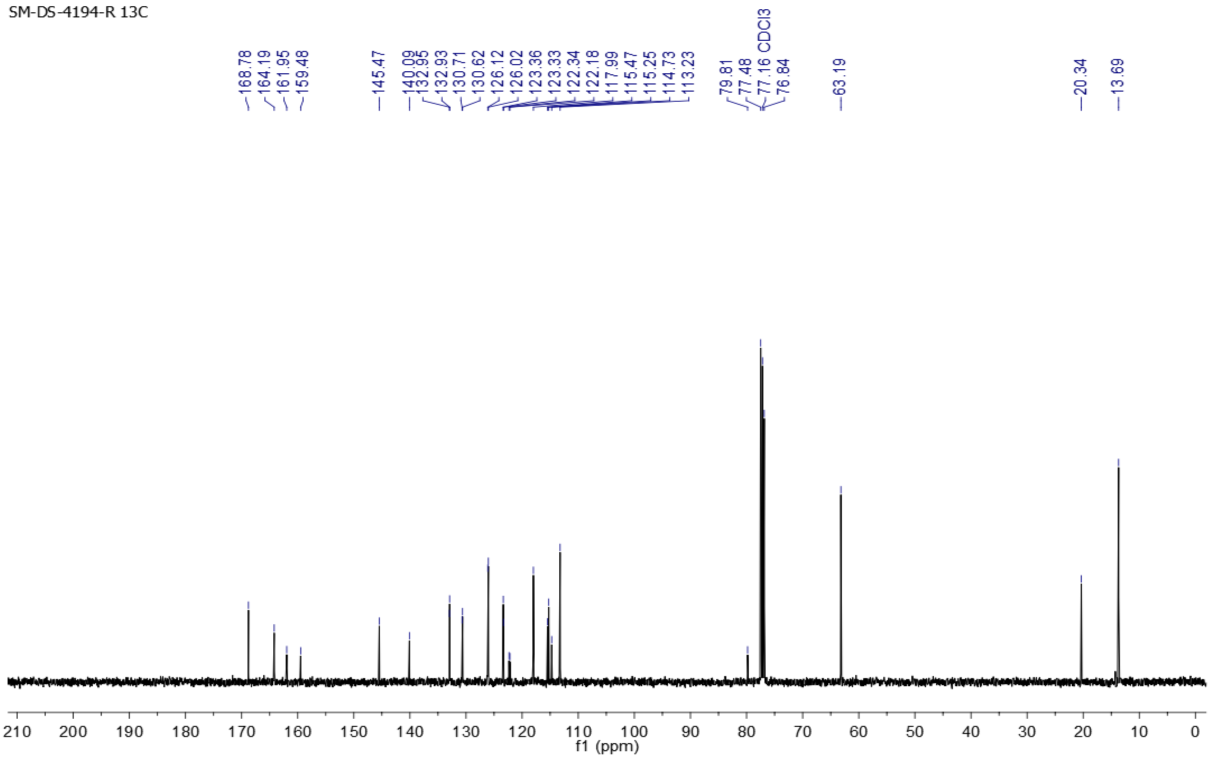

$^{19}F\{^{1}H\}$ NMR of **4h** (377 MHz, $CDCl_3$):

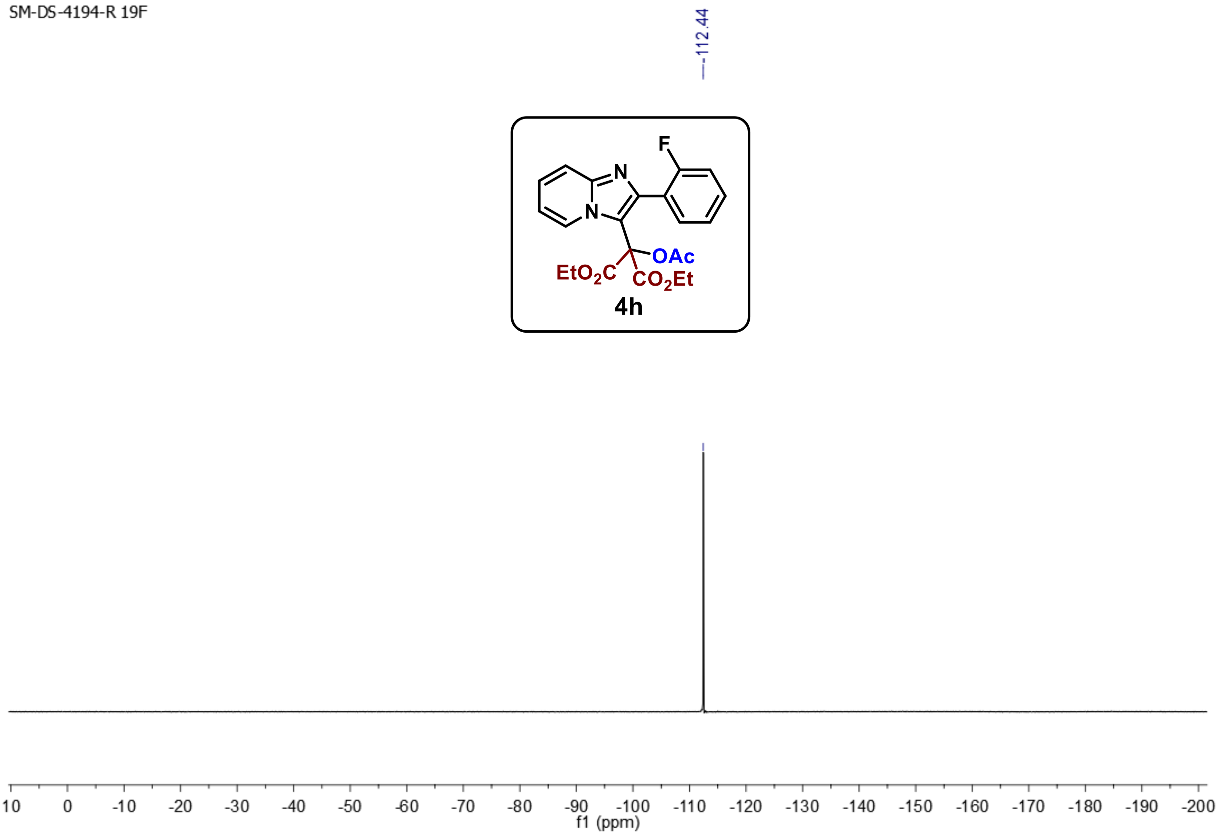

^{1}H NMR of **4i** (400 MHz, $CDCl_3$):

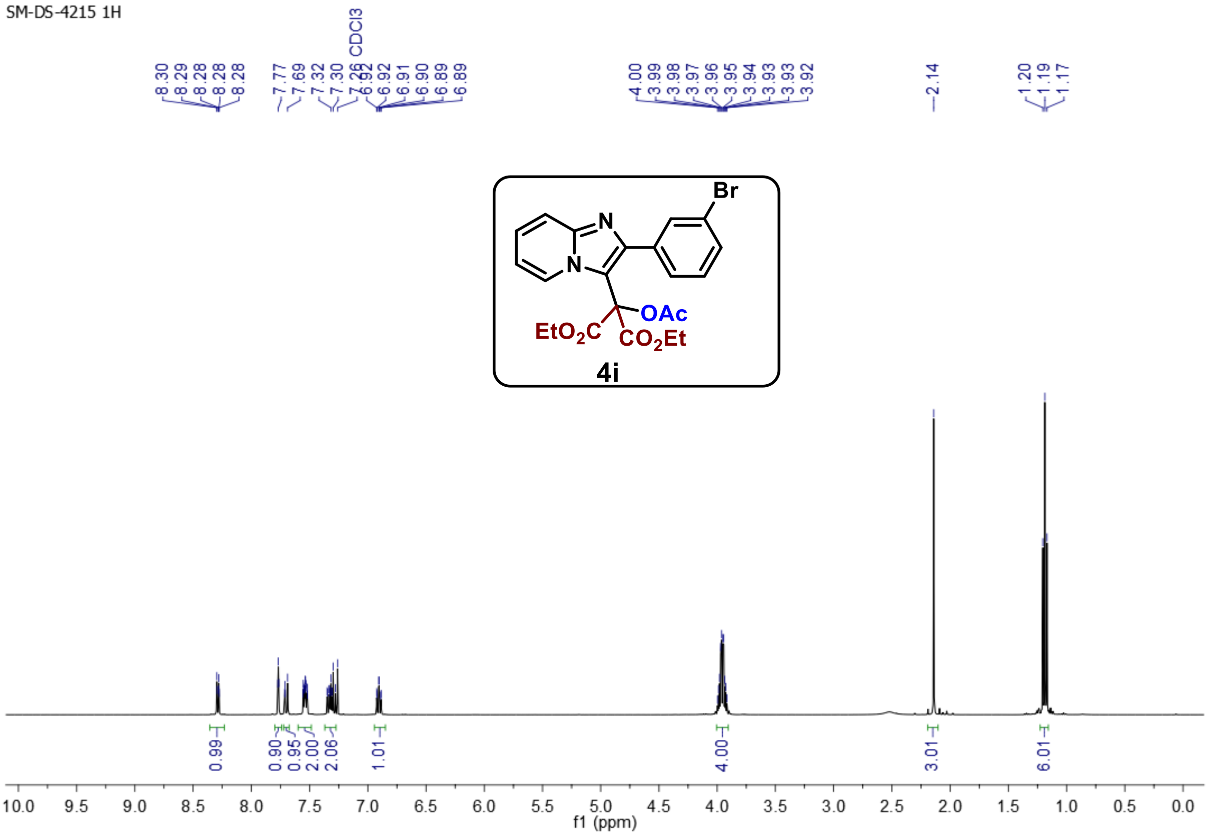

^{13}C{^{1}H} NMR of **4i** (101 MHz, $CDCl_3$):

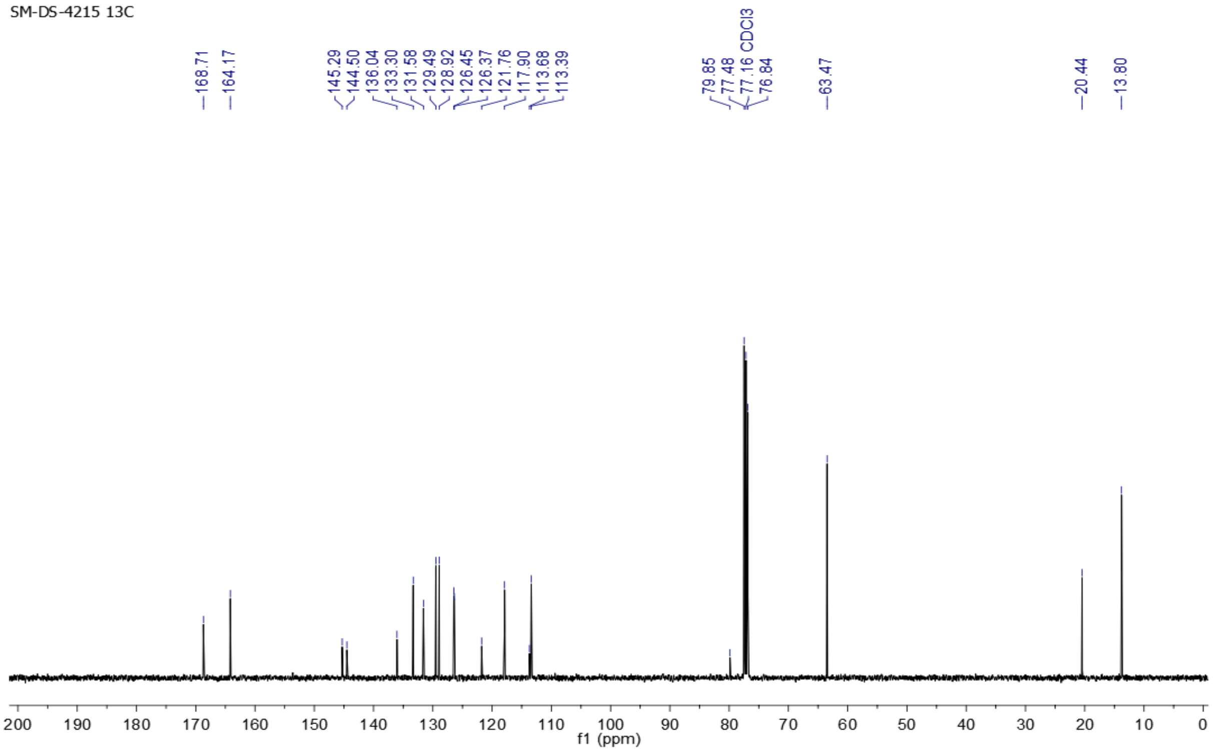

^{1}H NMR of **4j** (400 MHz, $CDCl_3$):

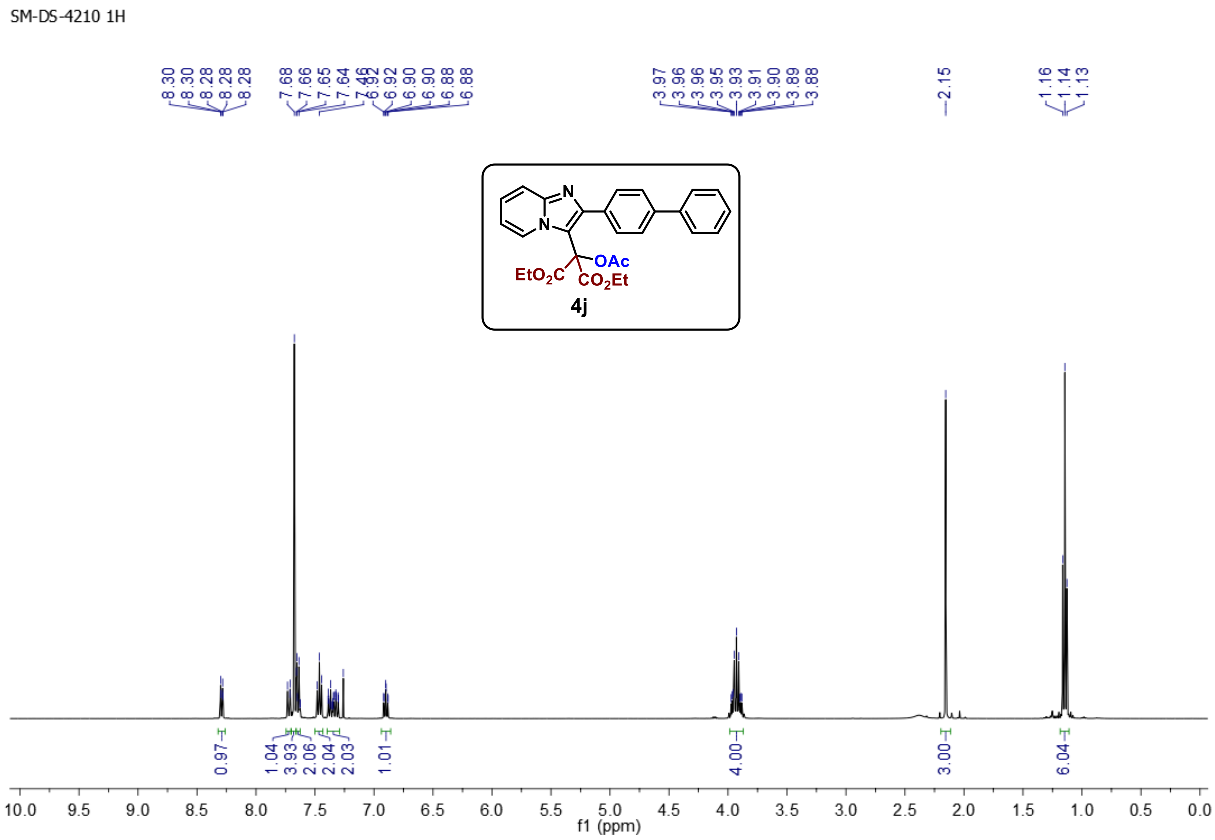

$^{13}C\{^{1}H\}$ NMR of **4j** (101 MHz, $CDCl_3$):

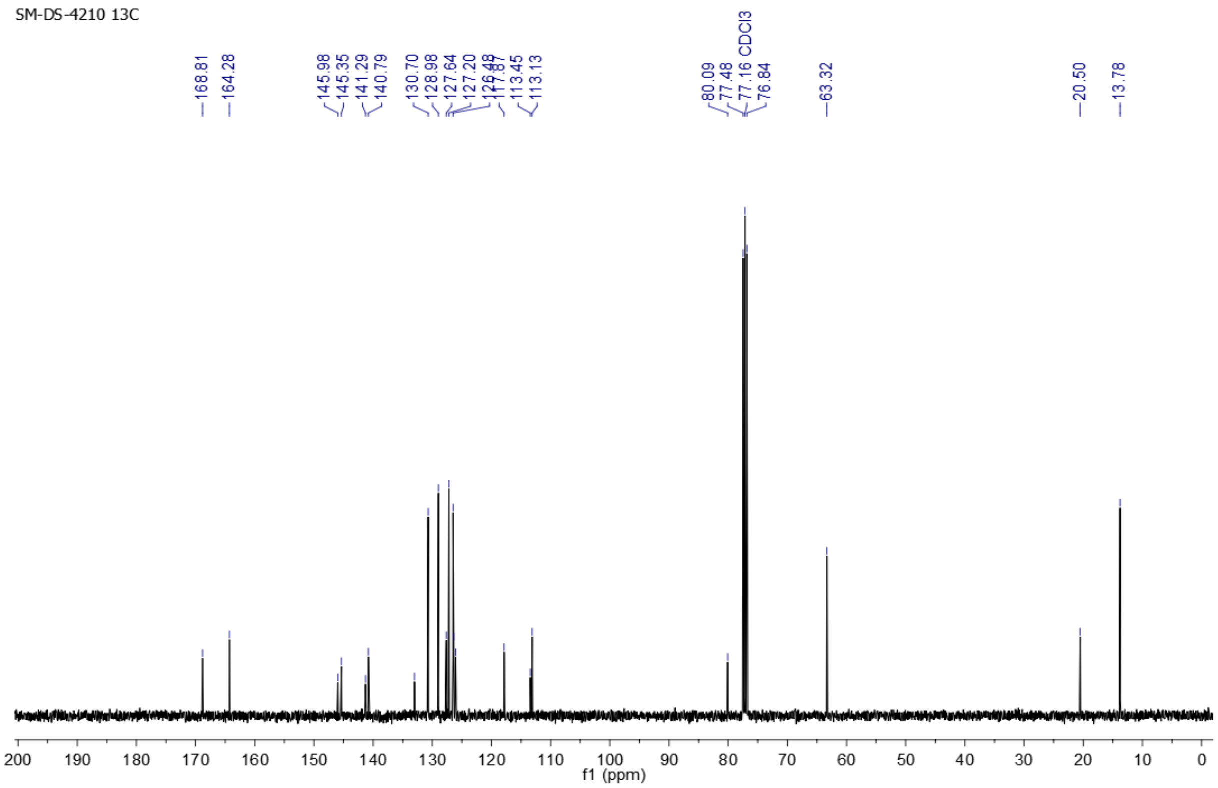

^{1}H NMR of **4k** (400 MHz, $CDCl_3$):

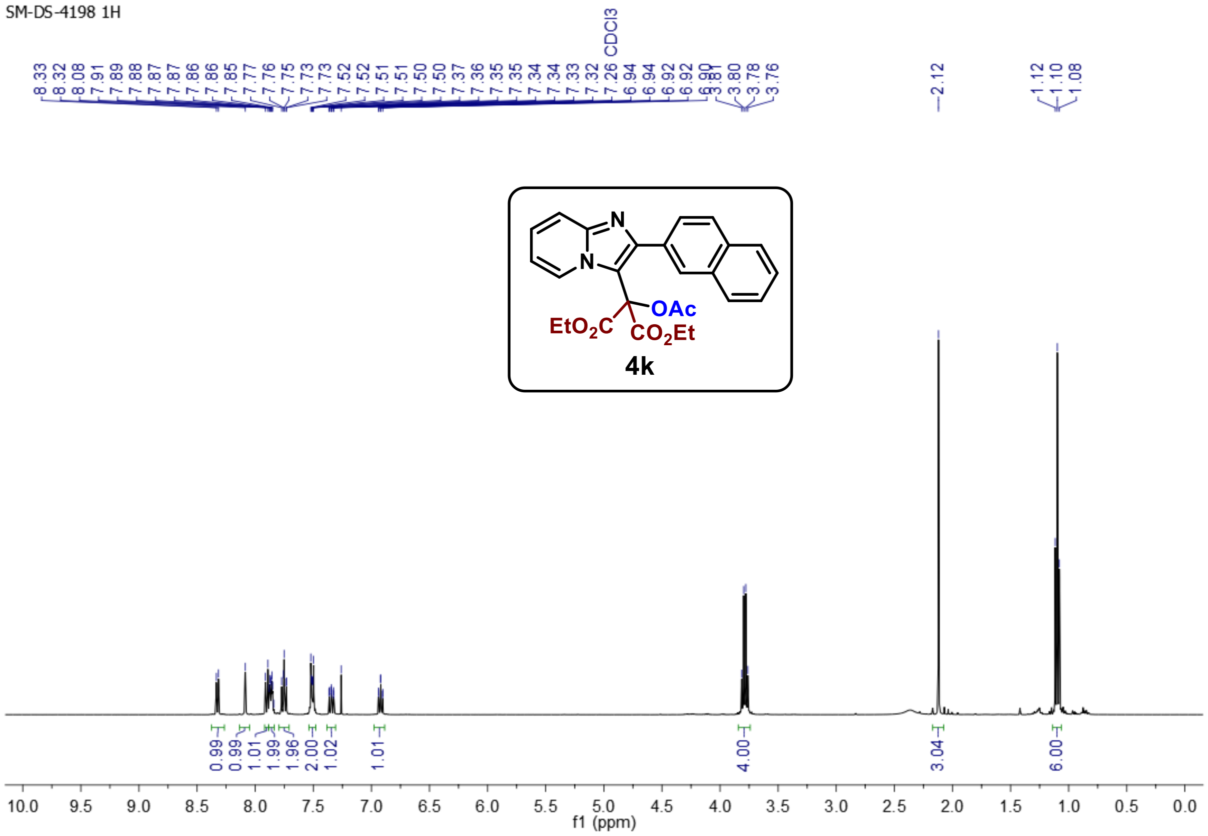

^{13}C{^{1}H} NMR of **4k** (101 MHz, $CDCl_3$):

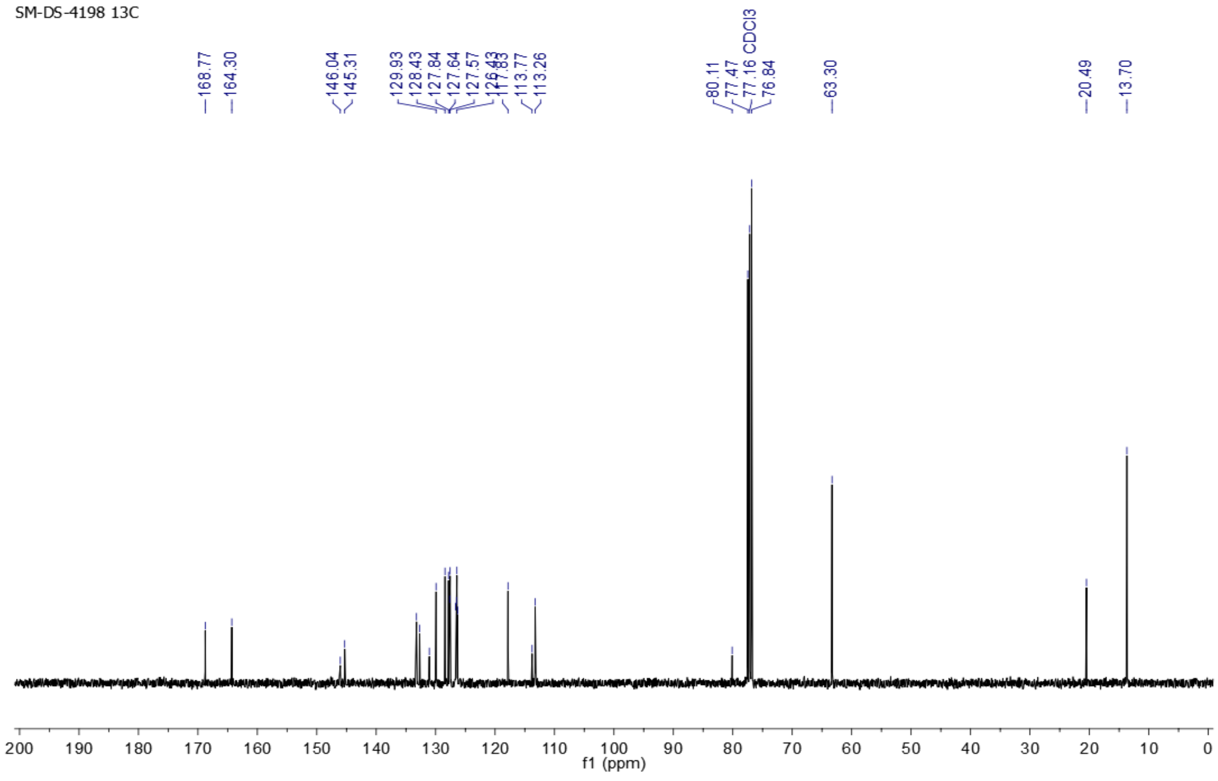

^{1}H NMR of **4l** (400 MHz, $CDCl_3$):

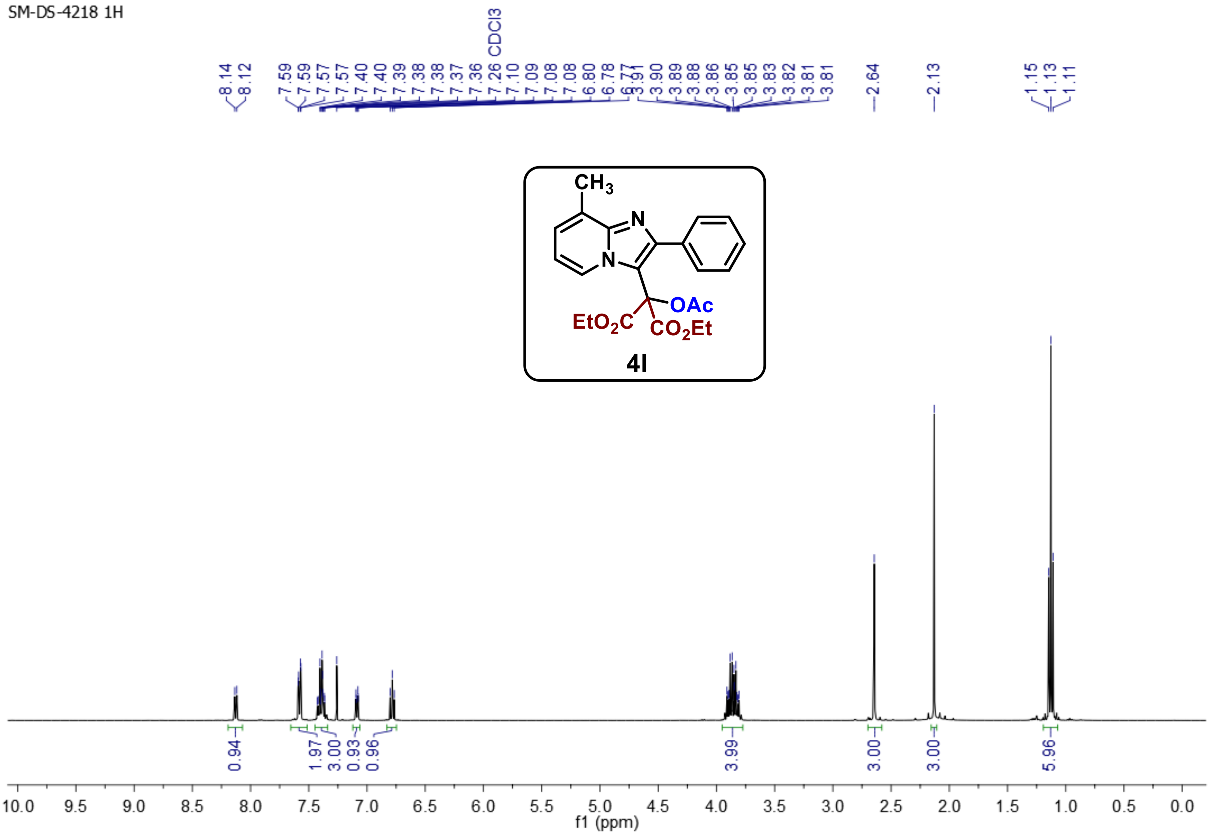

^{13}C{^{1}H} NMR of **4l** (101 MHz, $CDCl_3$):

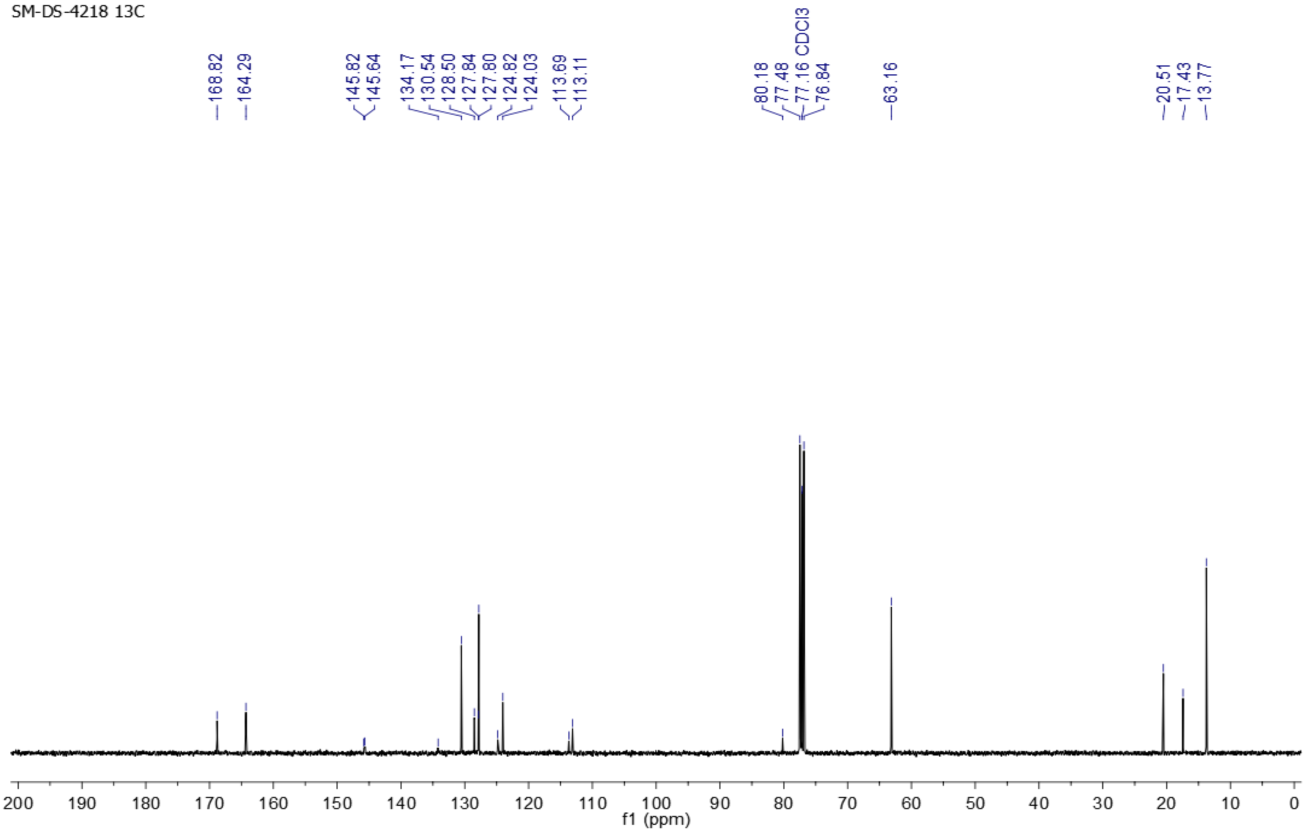

^{1}H NMR of **4m** (400 MHz, $CDCl_3$):

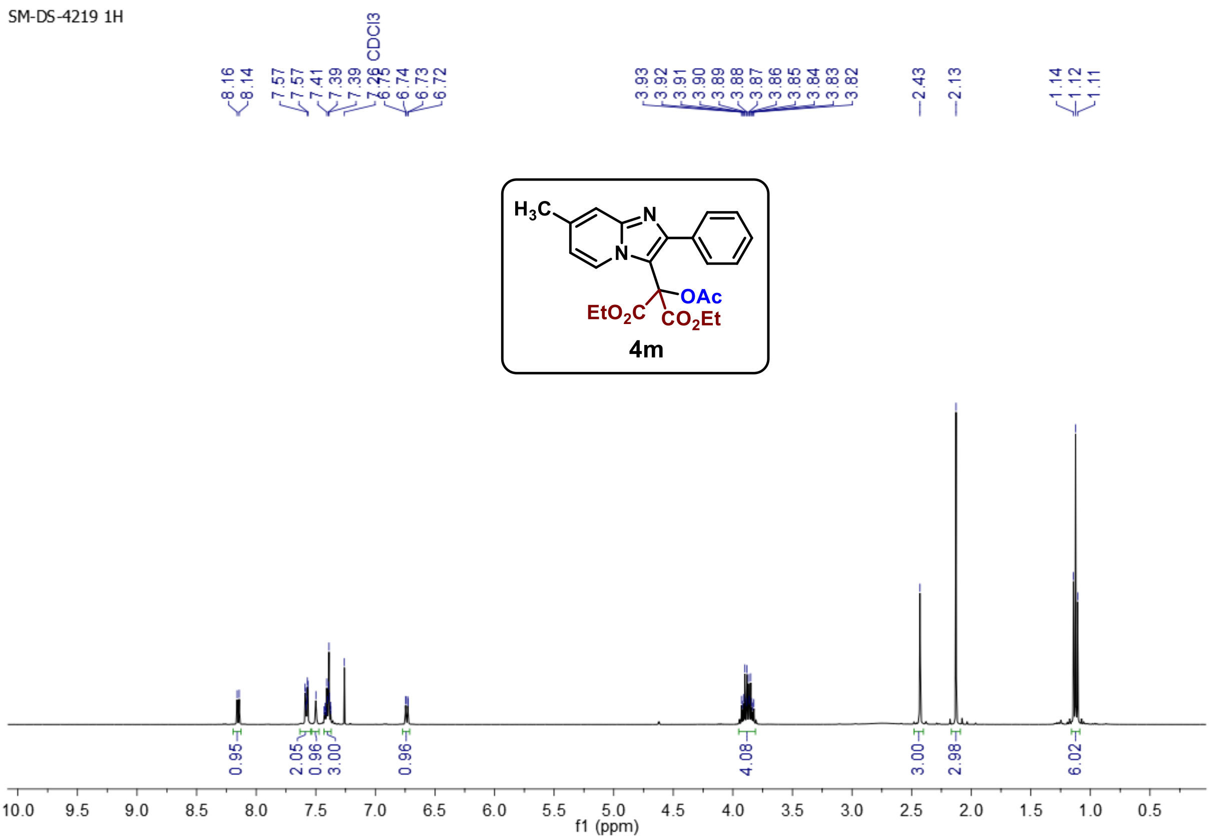

^{13}C{^{1}H} NMR of **4m** (101 MHz, $CDCl_3$):

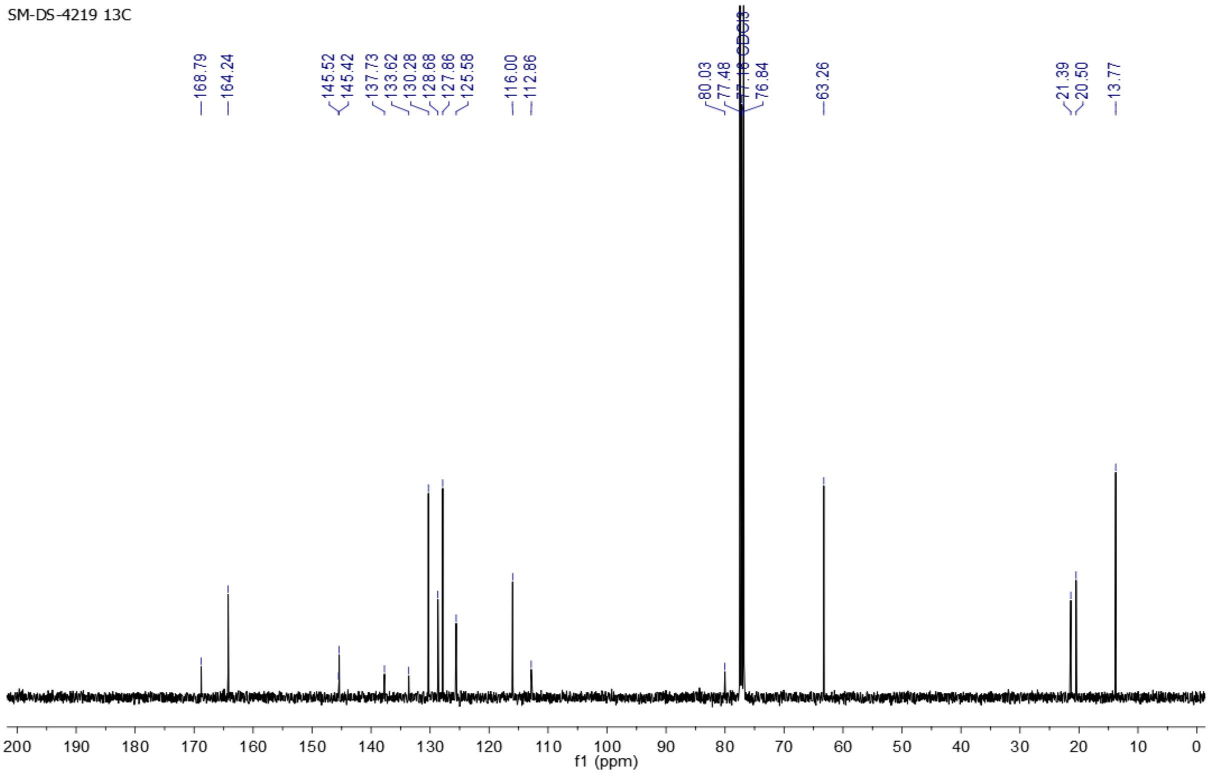

^{1}H NMR of **4n** (400 MHz, $CDCl_3$):

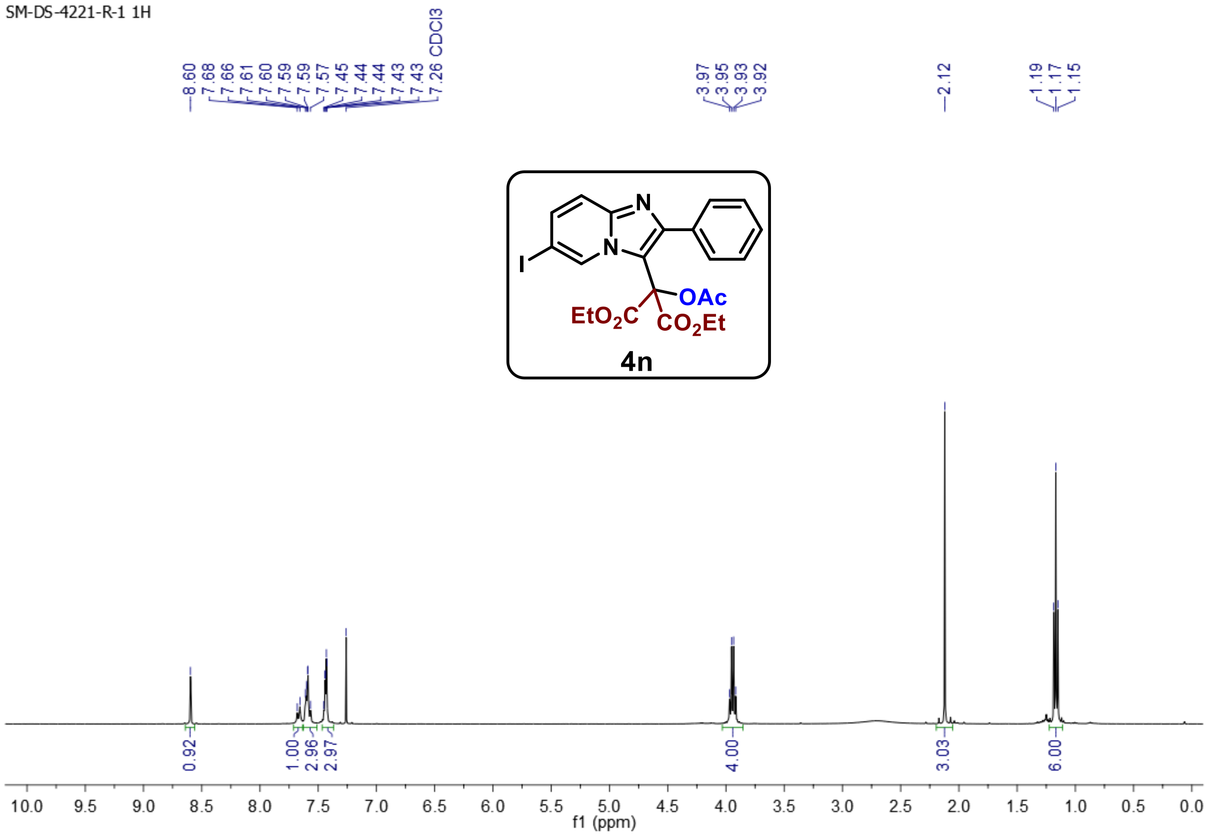

$^{13}C\{^{1}H\}$ NMR of **4n** (101 MHz, $CDCl_3$):

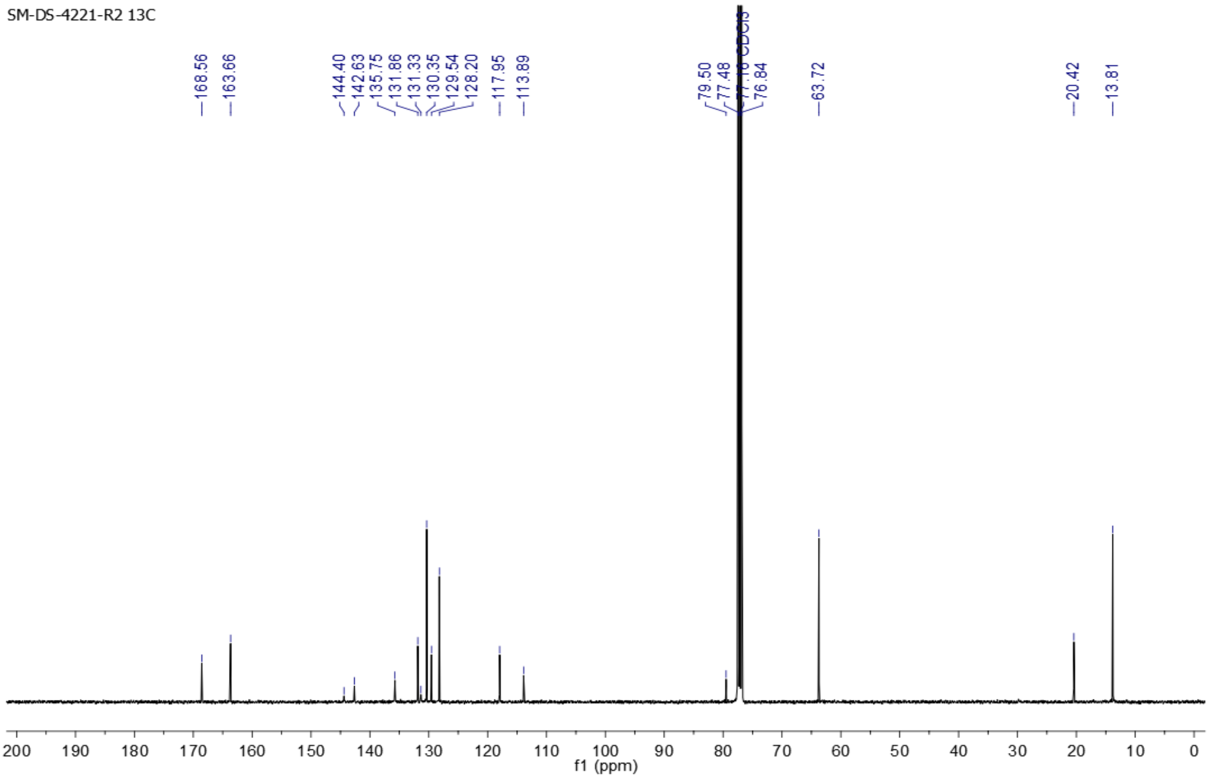

^{1}H NMR of **4o** (400 MHz, $CDCl_3$):

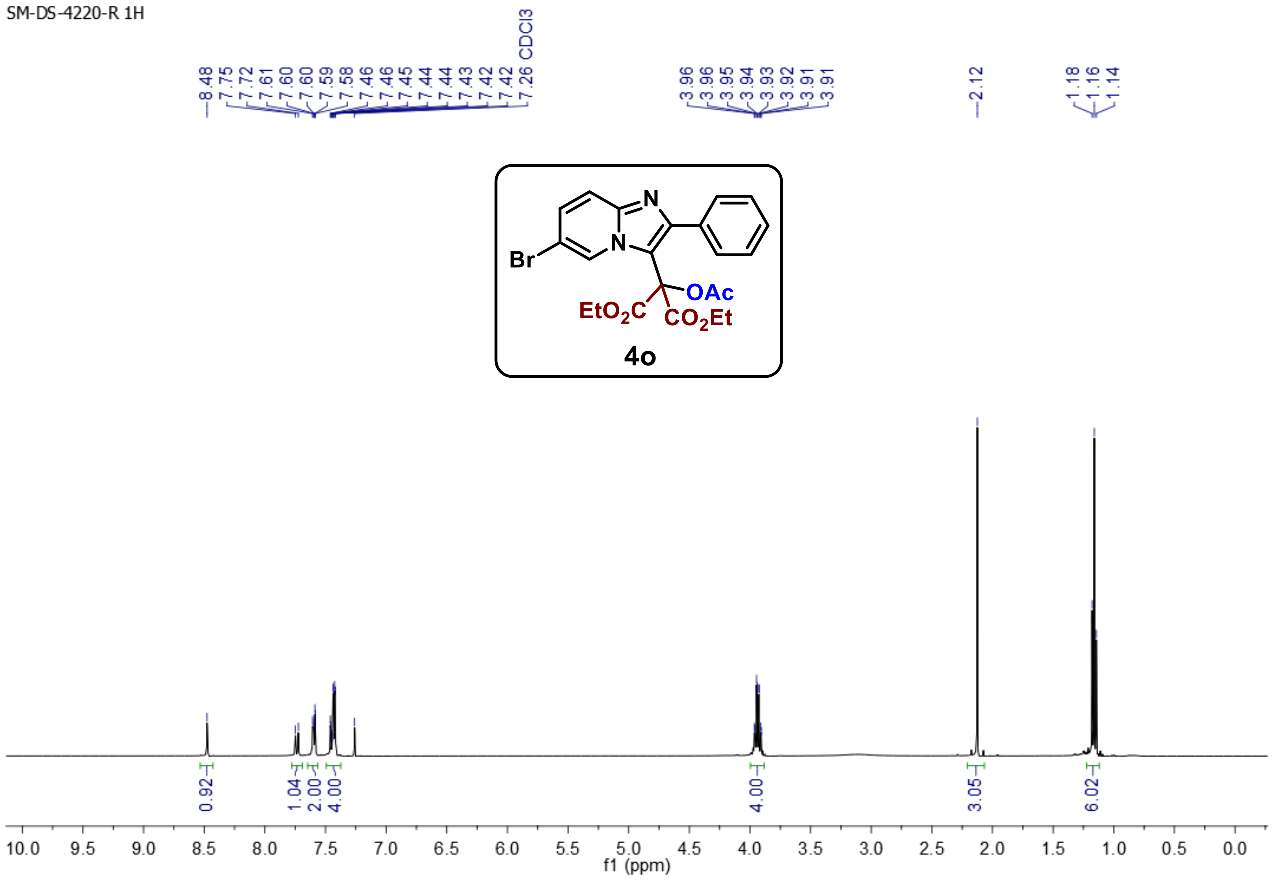

^{13}C{^{1}H} NMR of **4o** (101 MHz, $CDCl_3$):

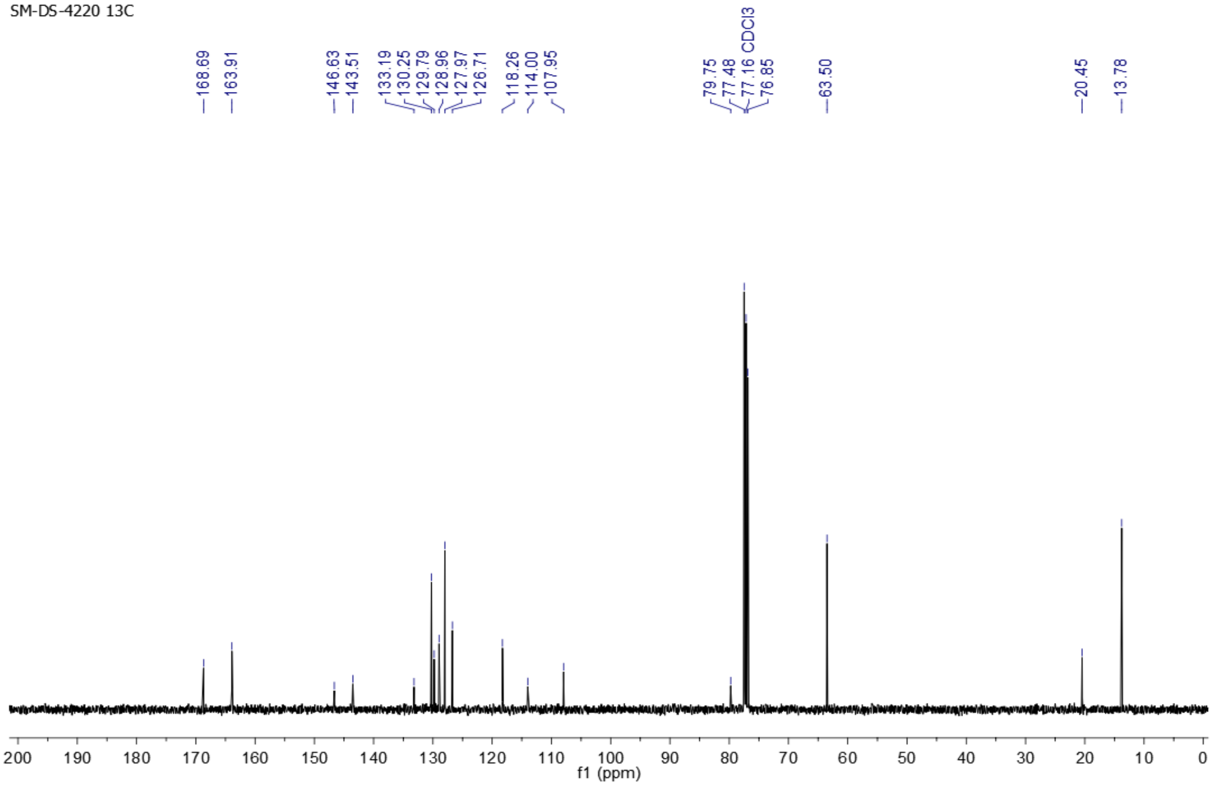

^{1}H NMR of **4p** (400 MHz, $CDCl_3$):

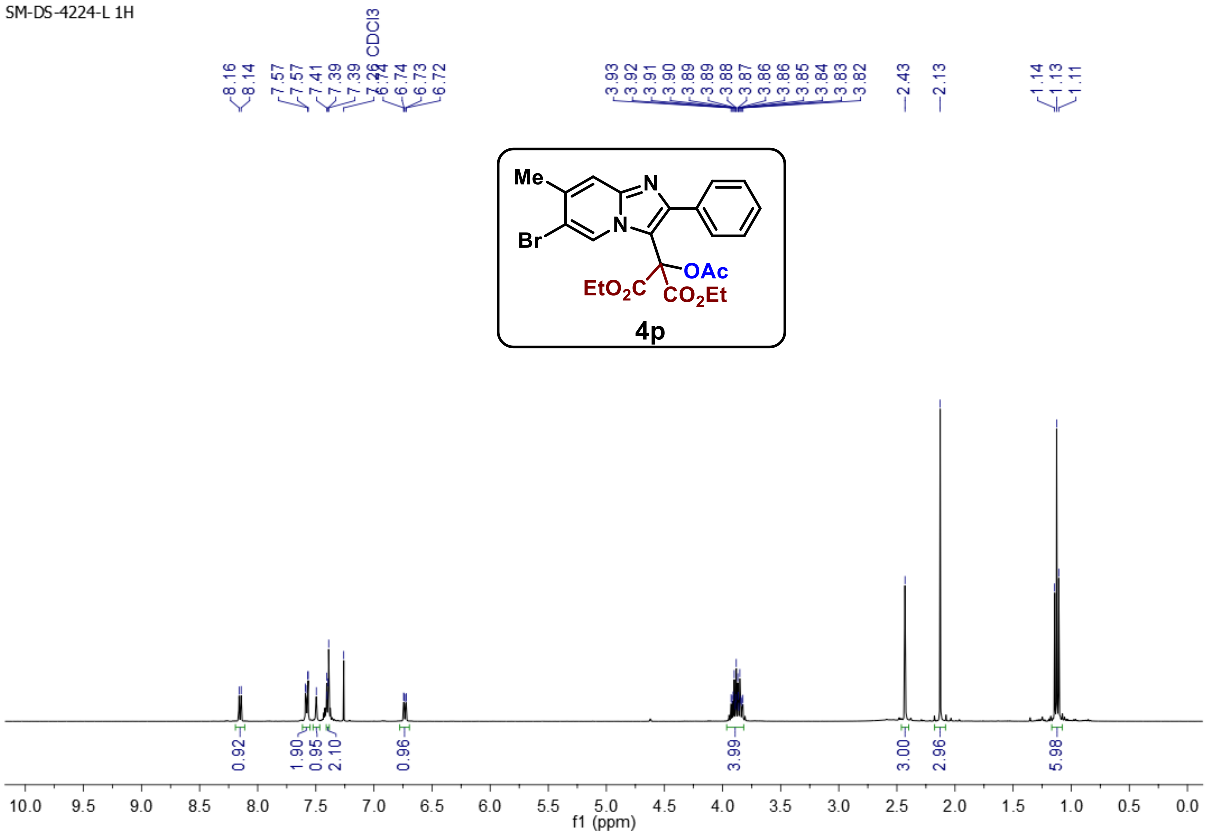

$^{13}C\{^{1}H\}$ NMR of **4p** (101 MHz, $CDCl_3$):

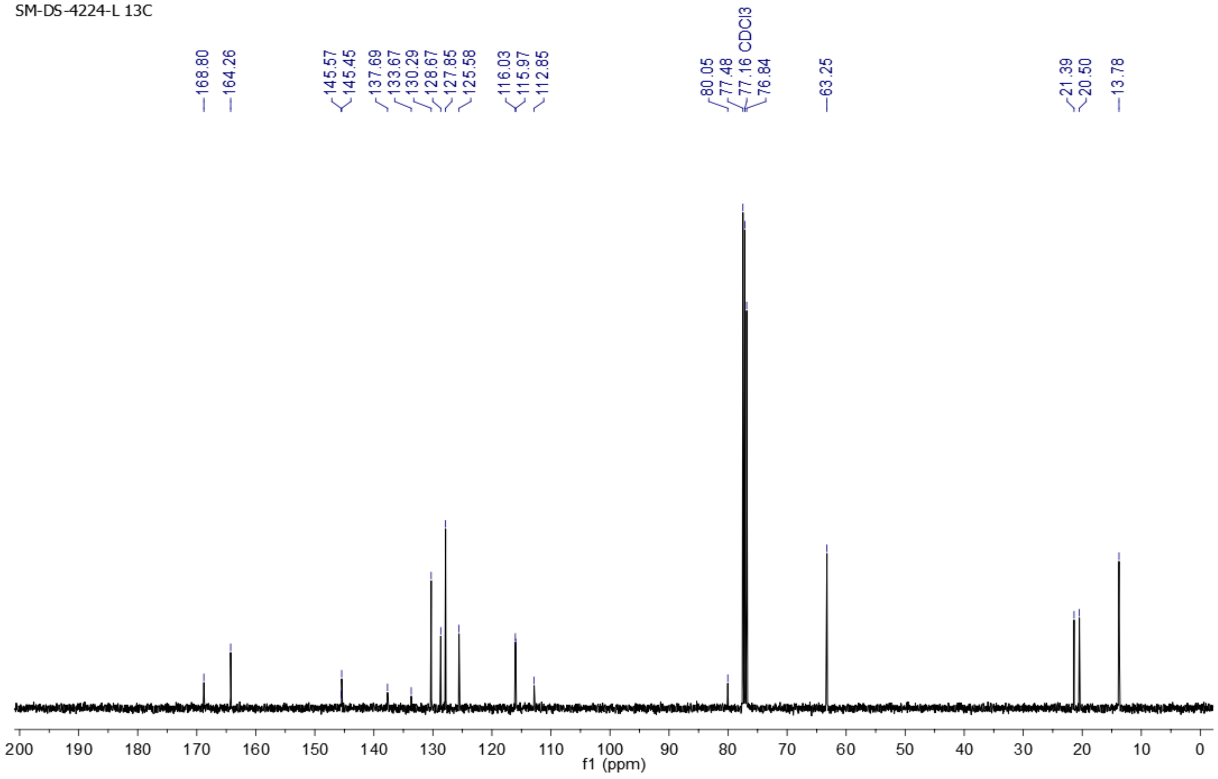

^1H NMR of **4q** (400 MHz, $CDCl_3$):

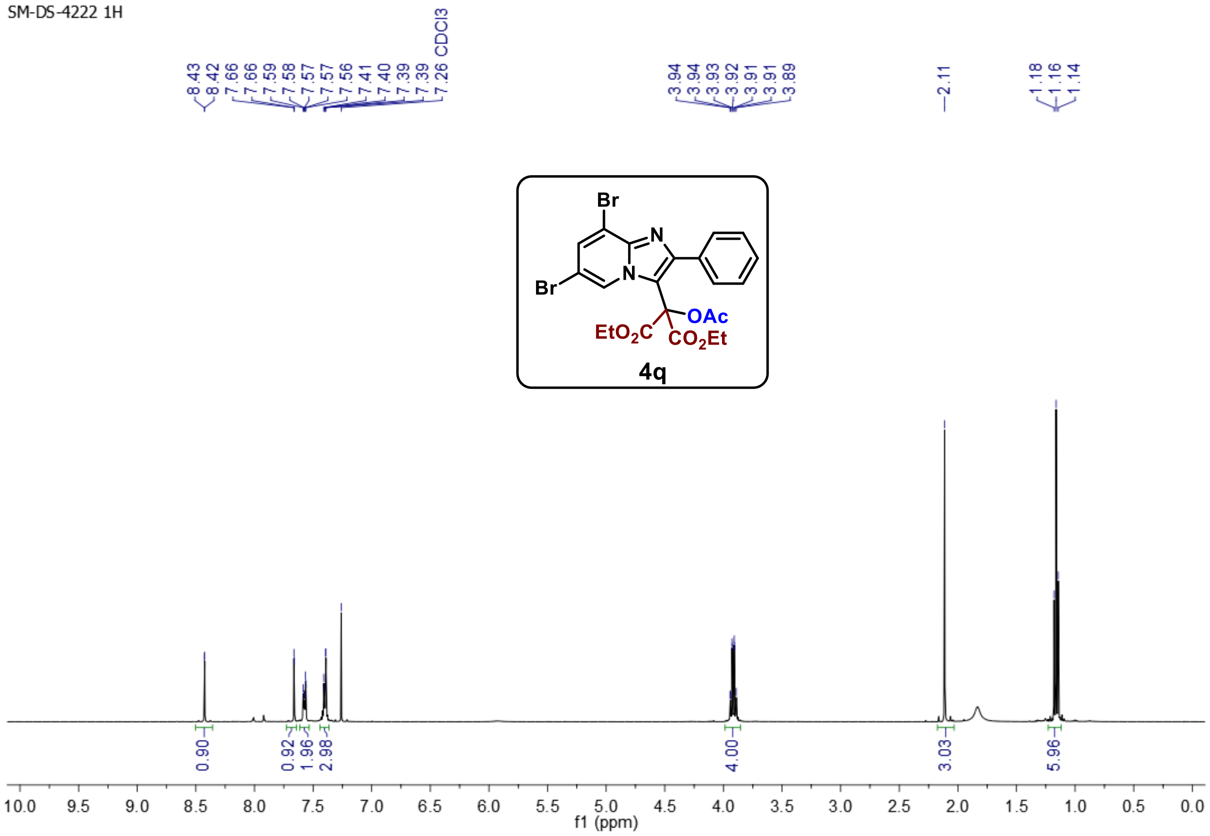

$^{13}C\{^1H\}$ NMR of **4q** (101 MHz, $CDCl_3$):

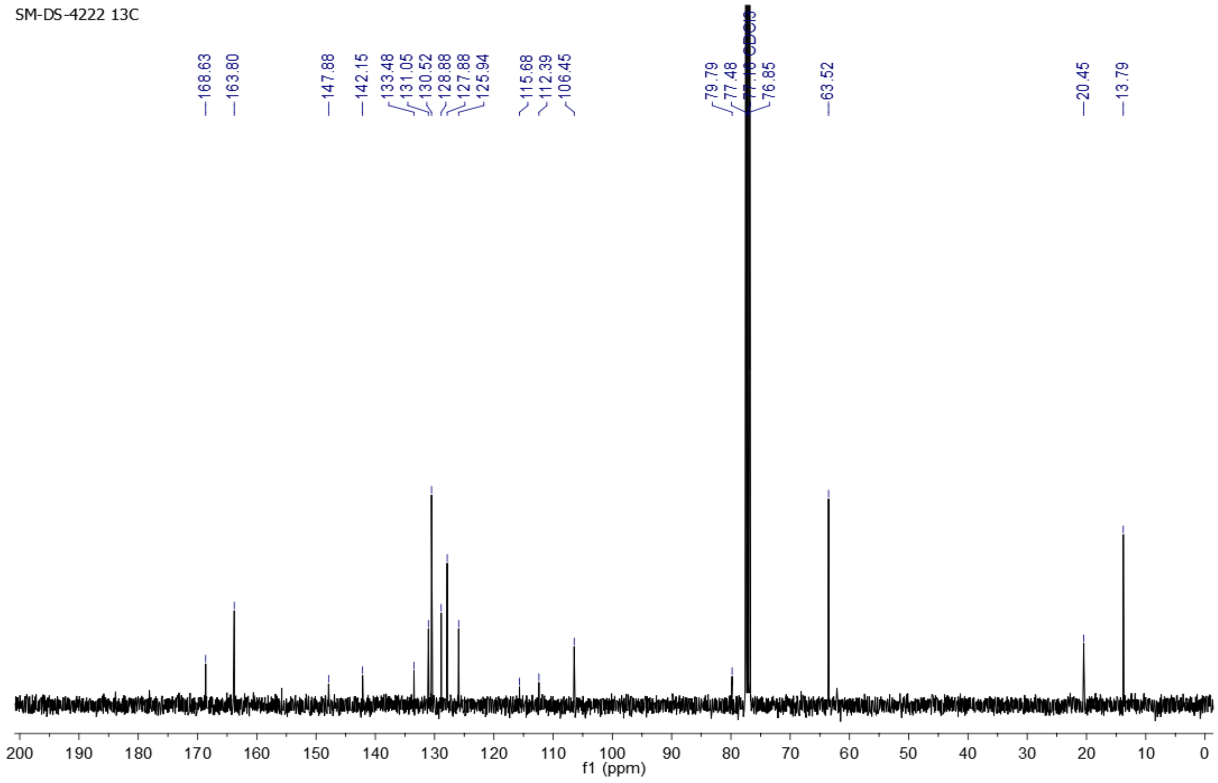

^{1}H NMR of **4r** (400 MHz, $CDCl_3$):

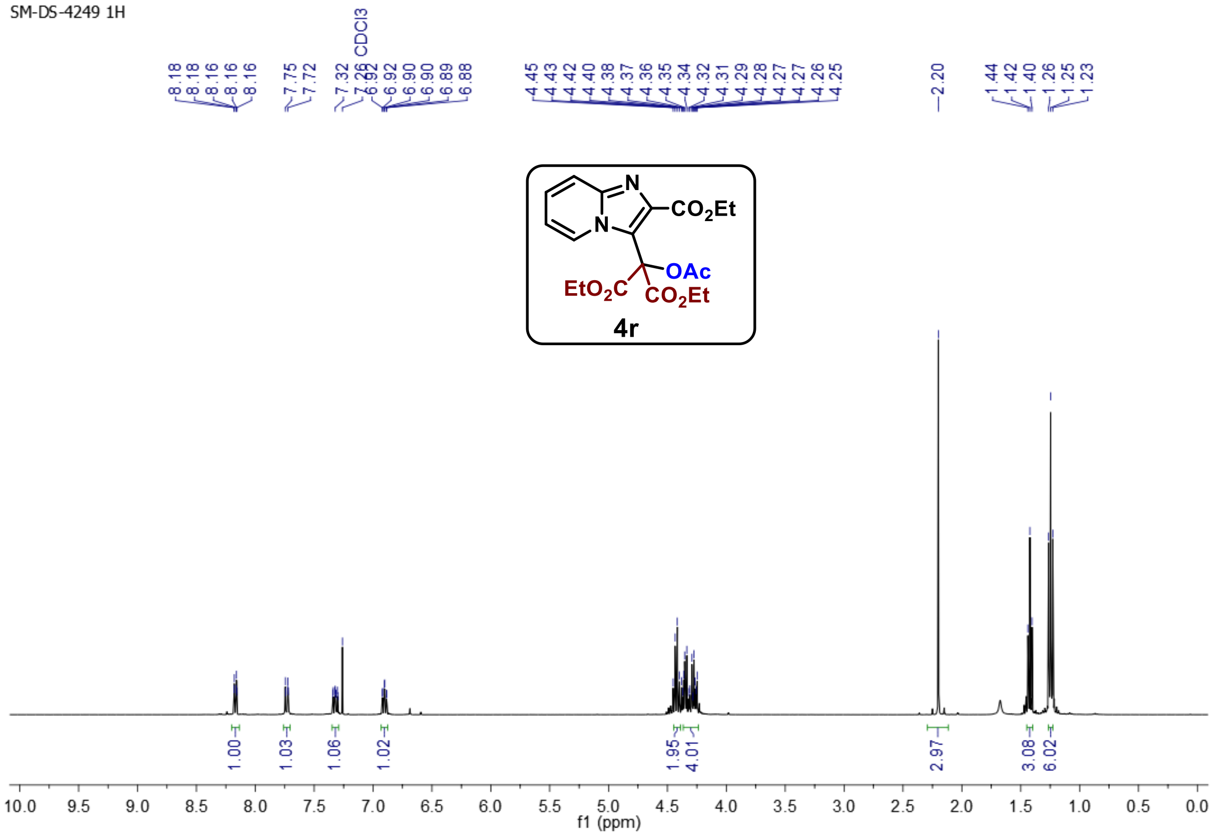

$^{13}C\{^{1}H\}$ NMR of **4r** (101 MHz, $CDCl_3$):

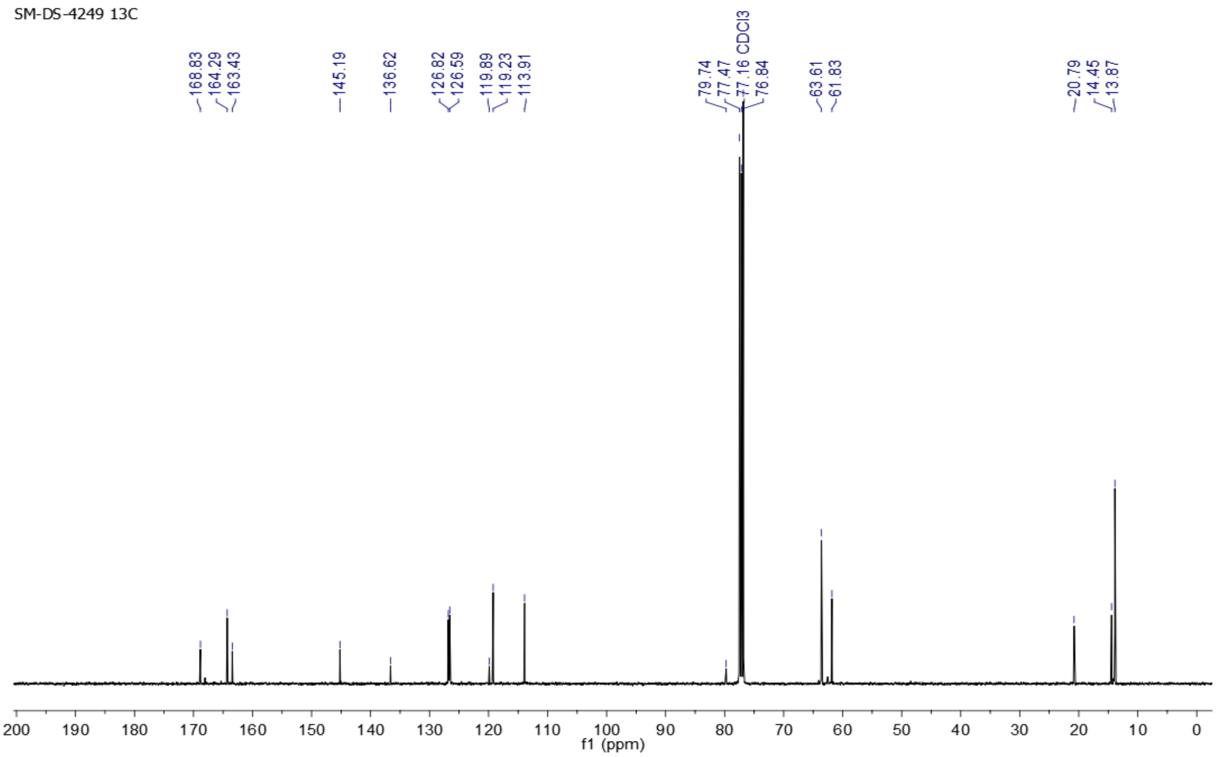

^{1}H NMR of **4s** (400 MHz, $CDCl_3$):

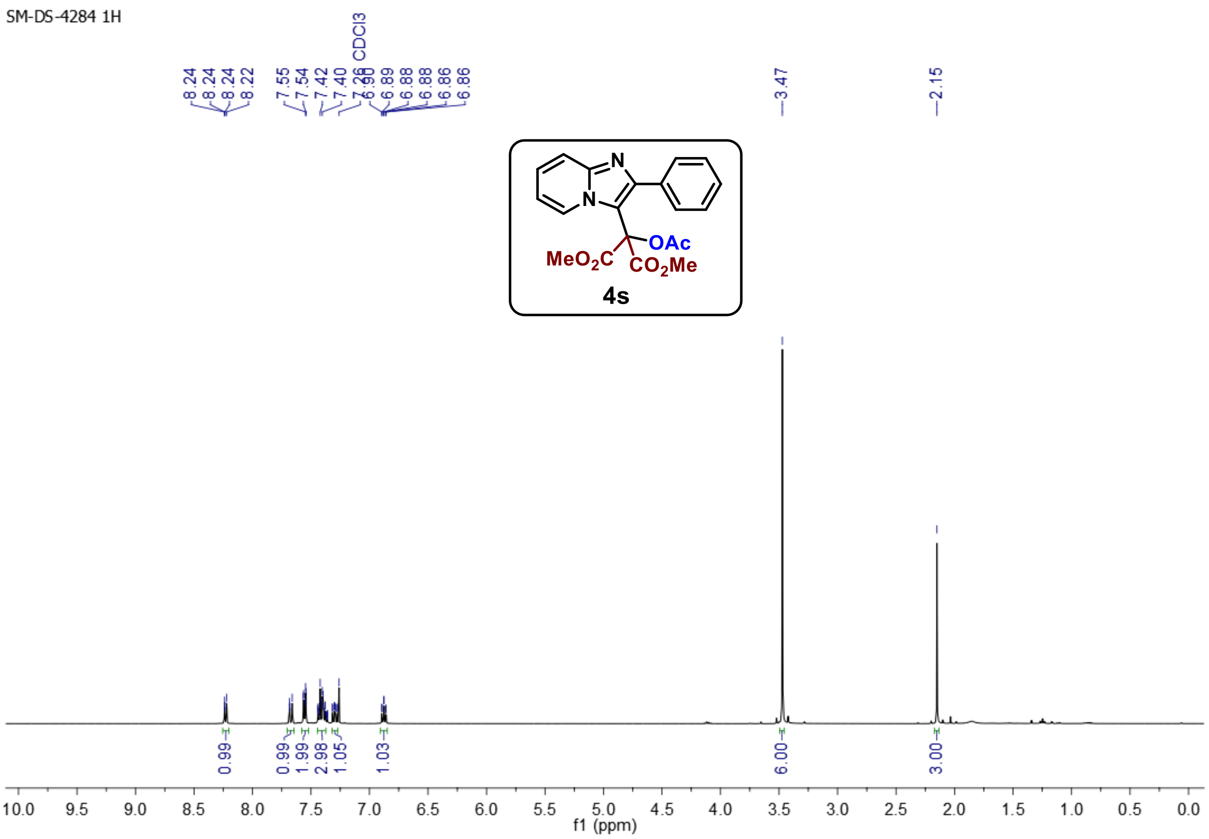

^{13}C{^{1}H} NMR of **4s** (101 MHz, $CDCl_3$):

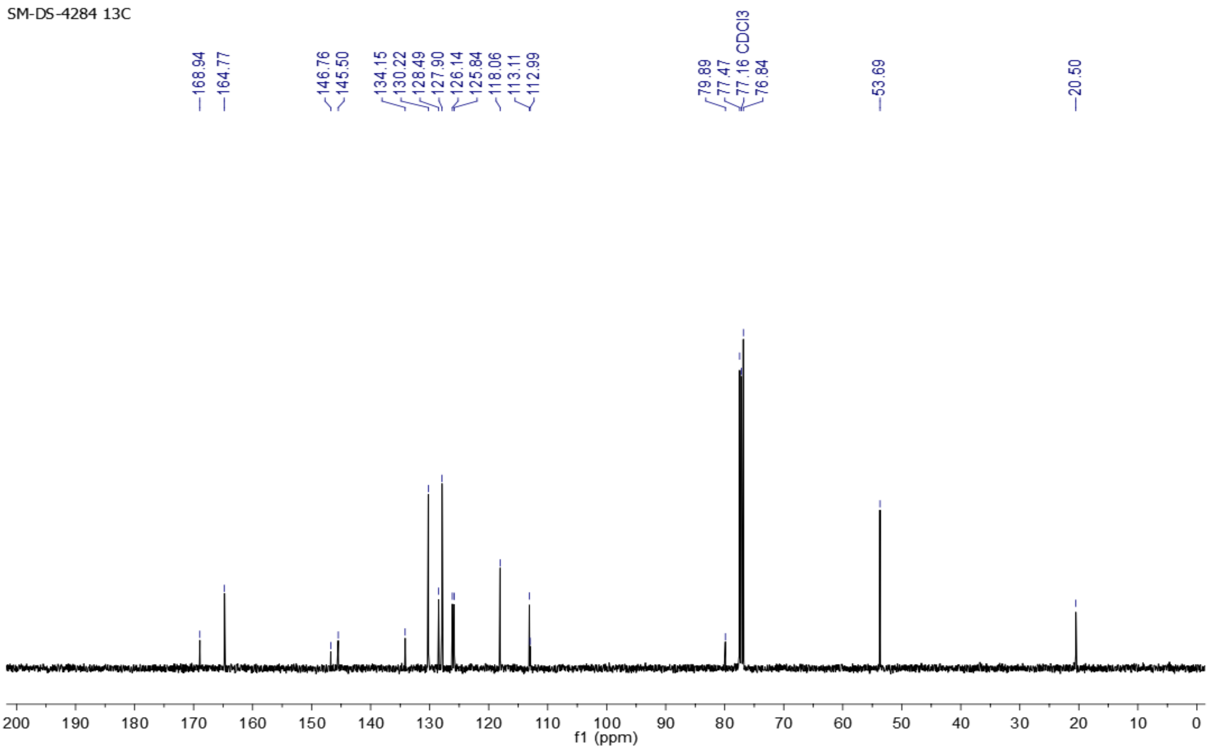

^{1}H NMR of **4t** (400 MHz, $CDCl_3$):

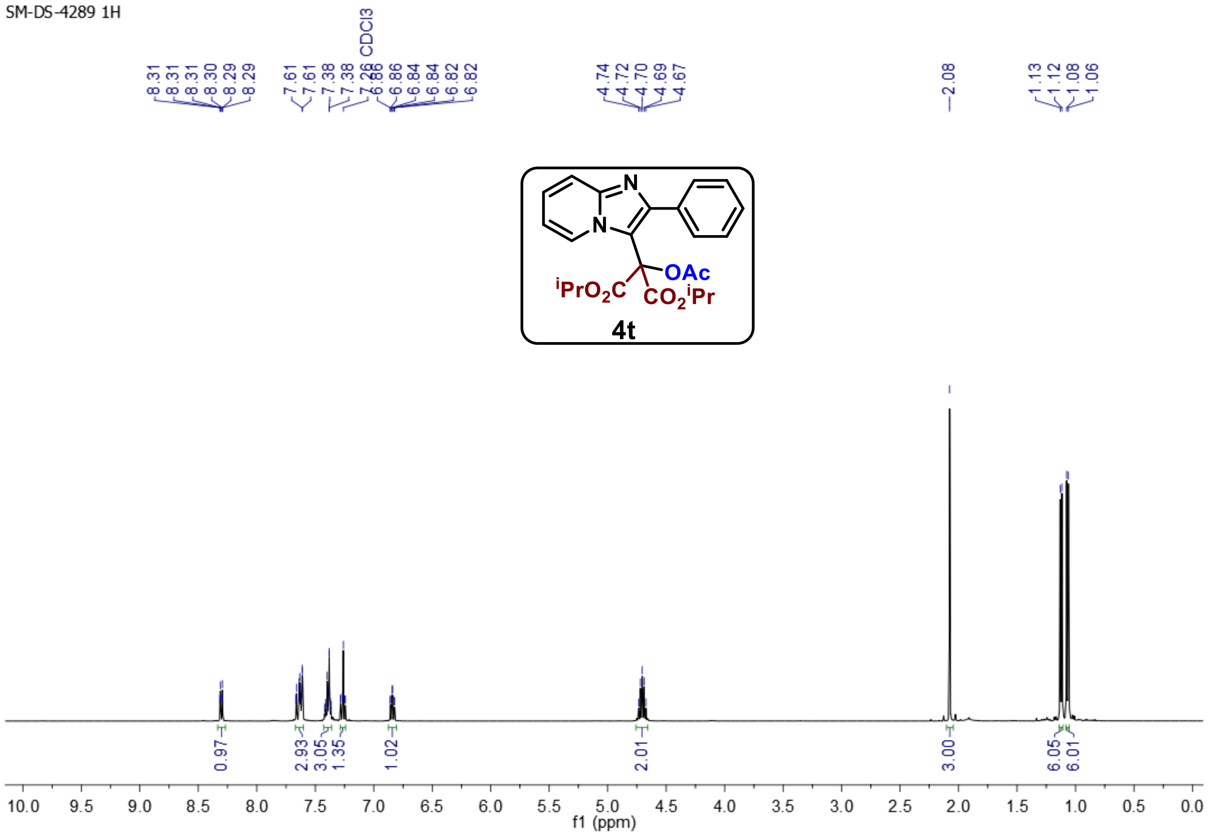

$^{13}C\{^{1}H\}$ NMR of **4t** (101 MHz, $CDCl_3$):

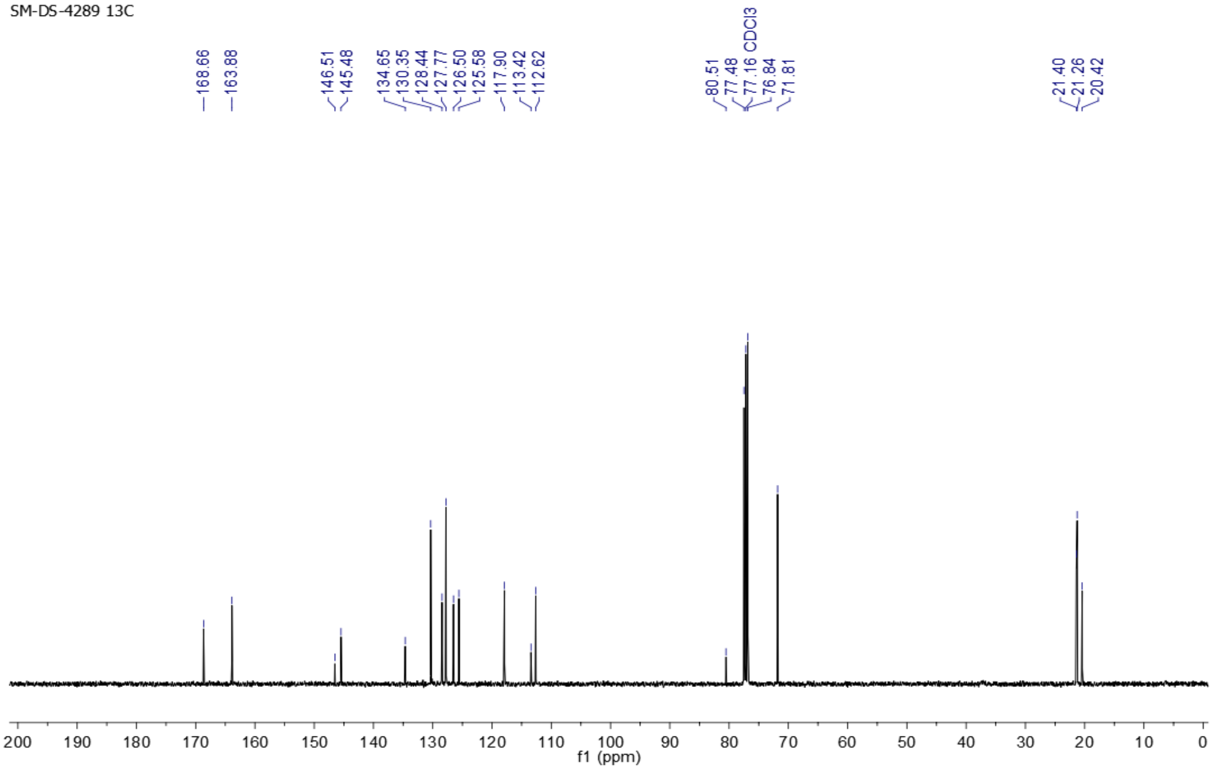

^{1}H NMR of **4u** (400 MHz, $CDCl_3$):

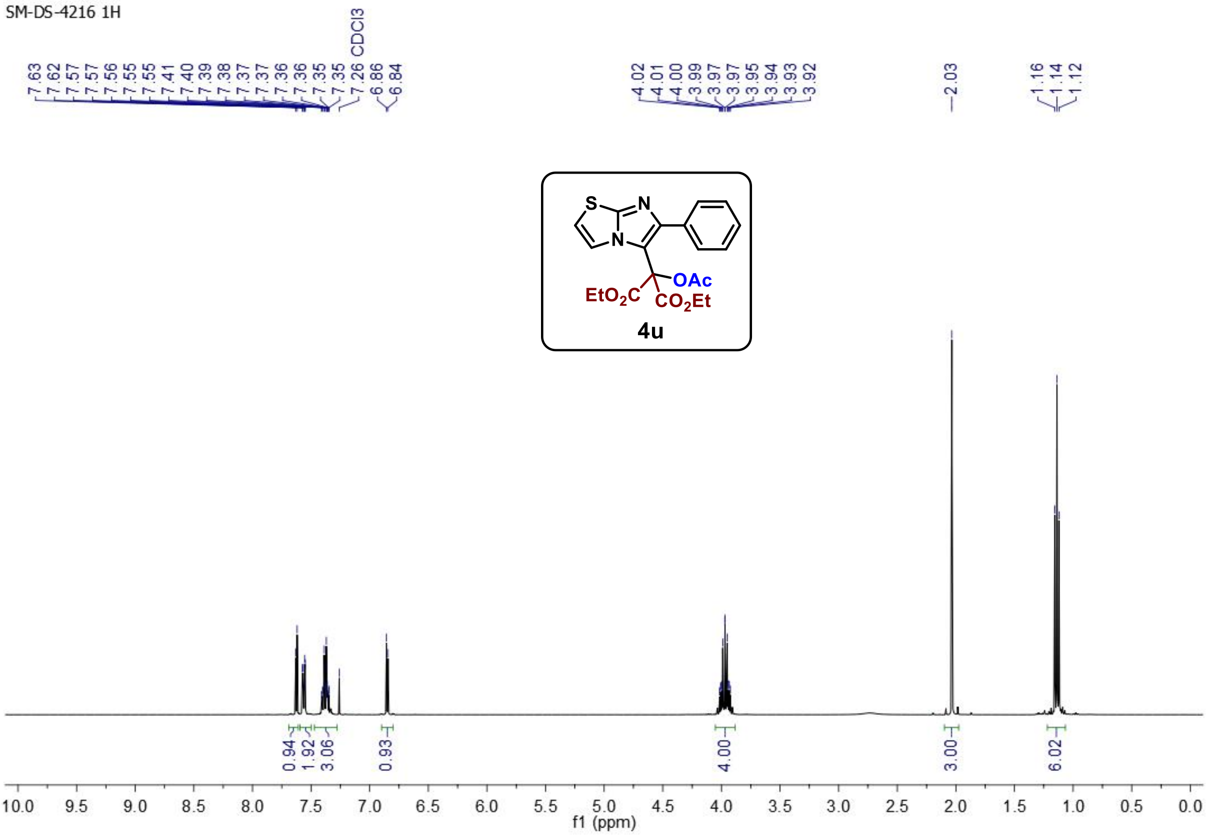

^{13}C{^{1}H} NMR of **4u** (101 MHz, $CDCl_3$):

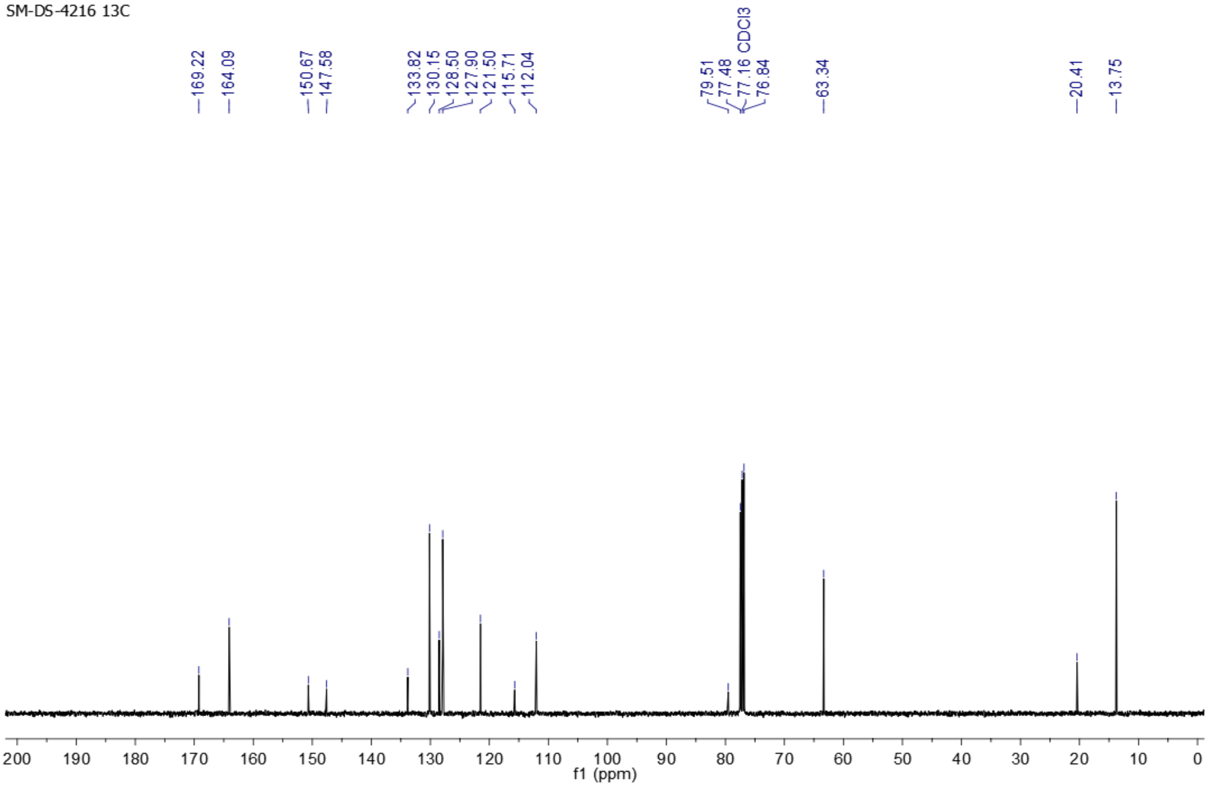

^{1}H NMR of **4v** (400 MHz, $CDCl_3$):

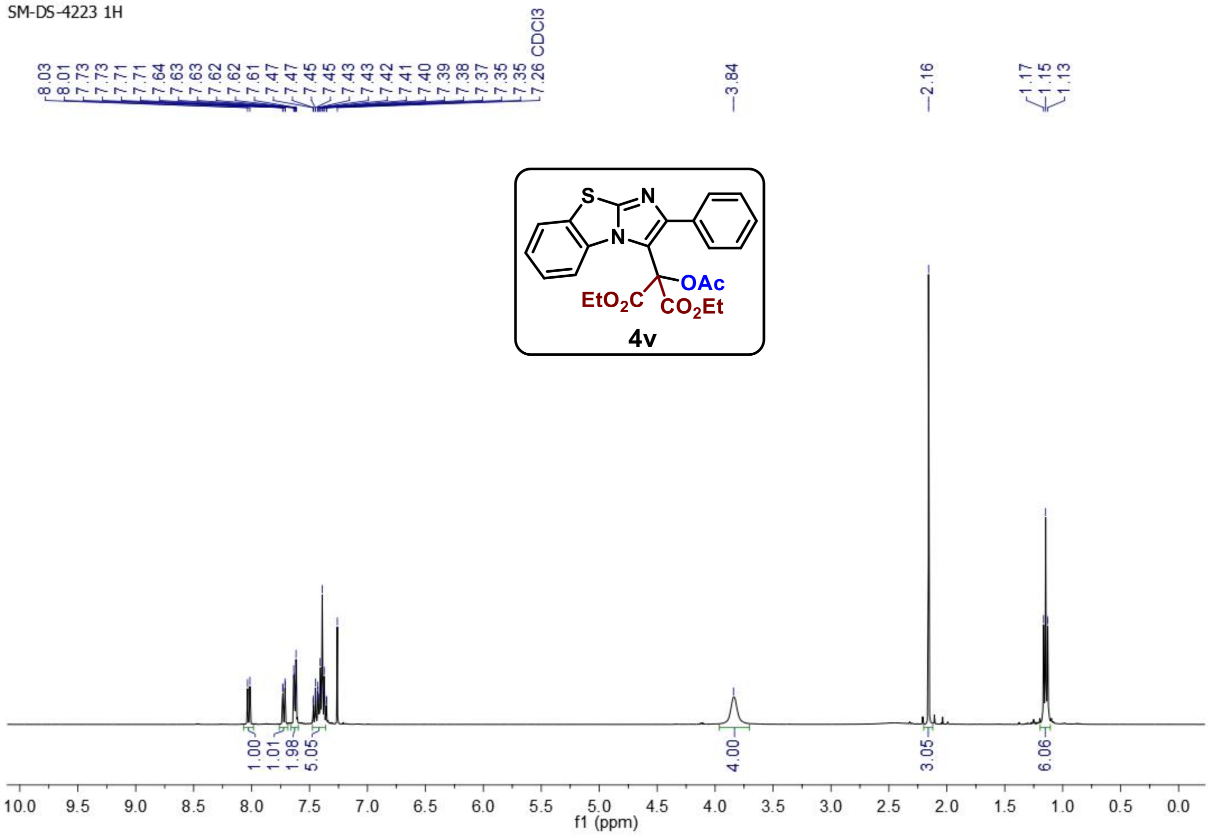

$^{13}C\{^{1}H\}$ NMR of **4v** (101 MHz, $CDCl_3$):

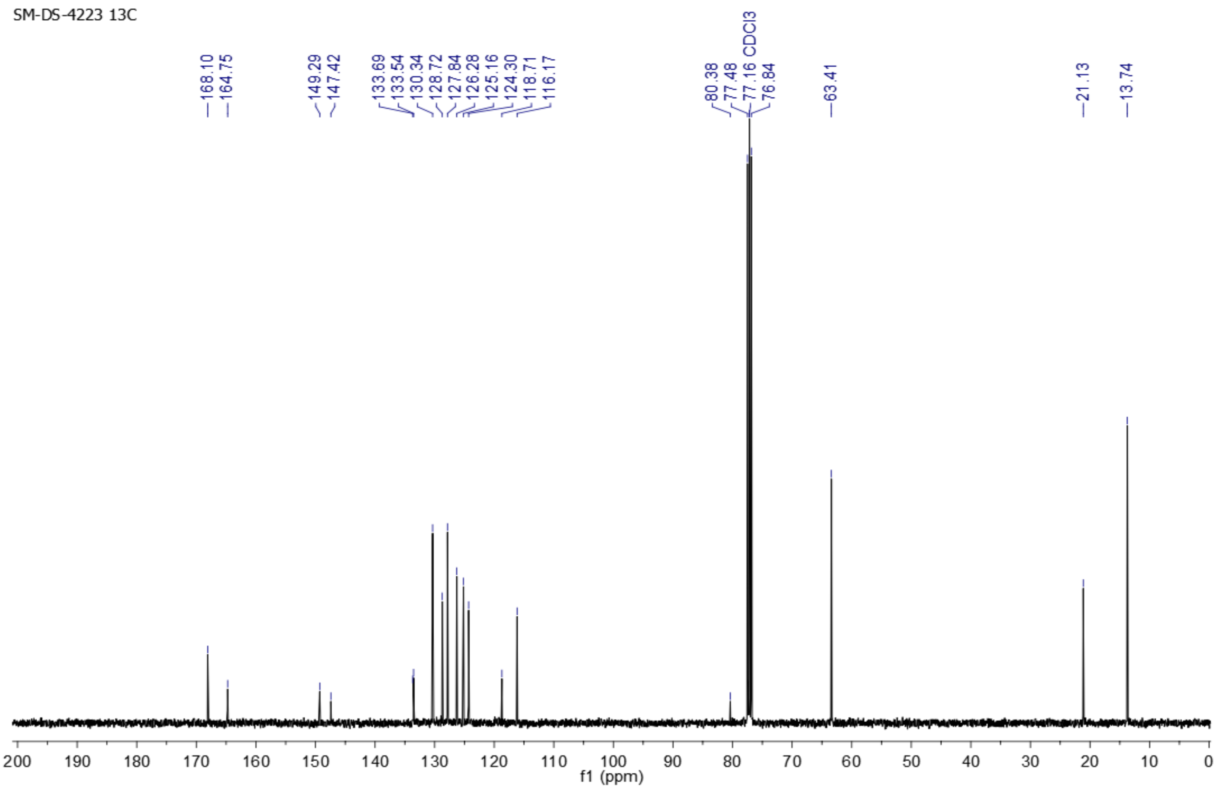

^{1}H NMR of **4w** (400 MHz, $CDCl_3$):

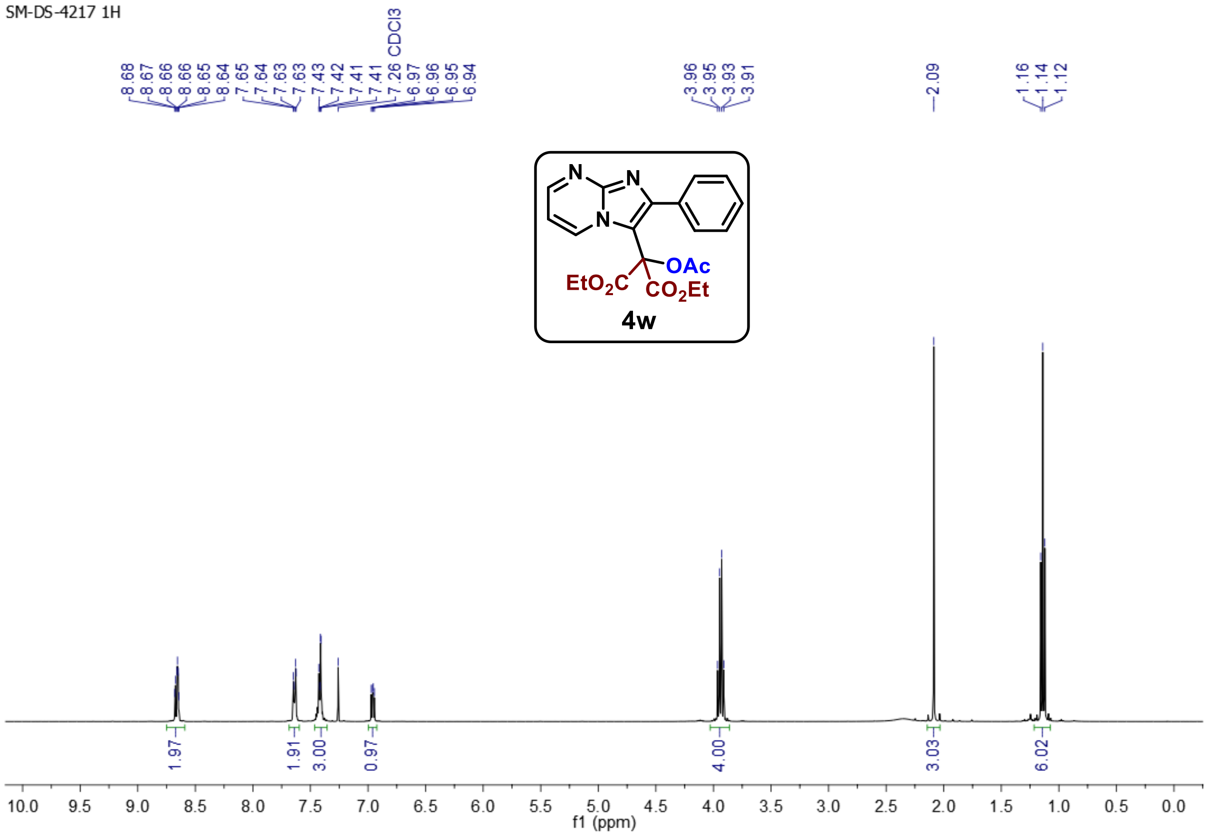

^{13}C{^{1}H} NMR of **4w** (101 MHz, $CDCl_3$):

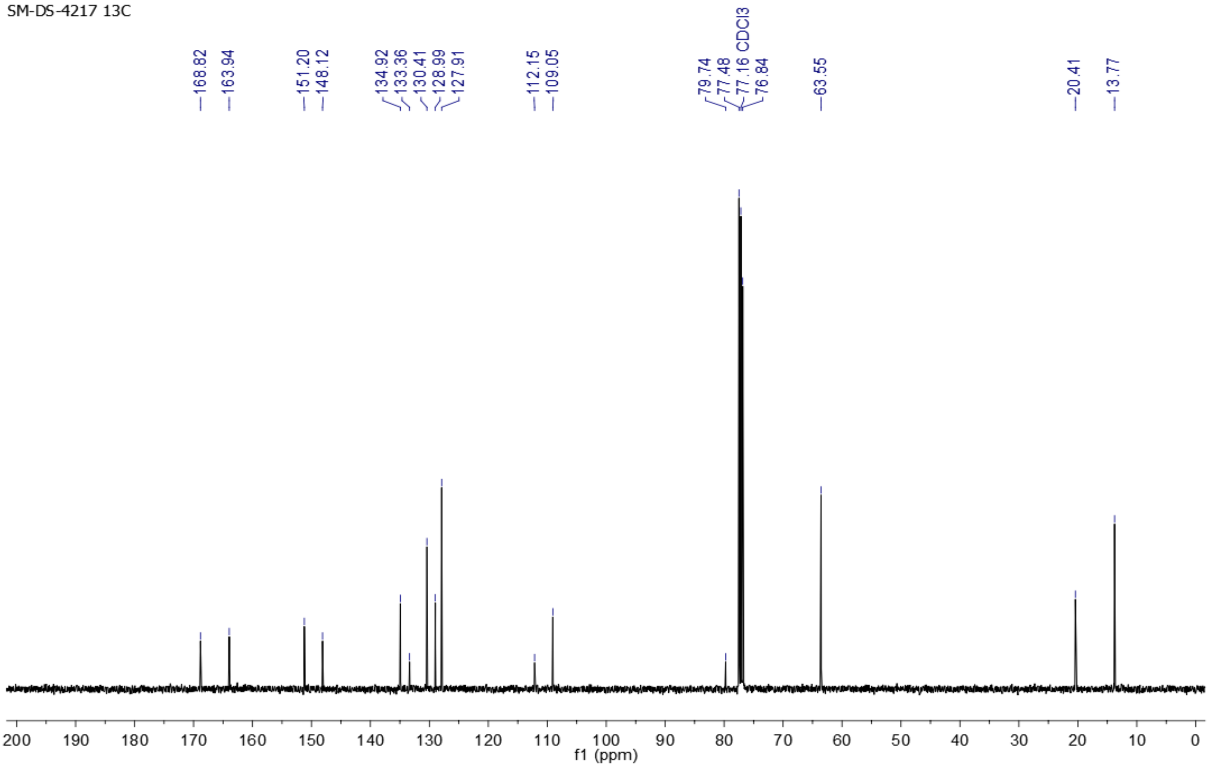

^{1}H NMR of **4x** (400 MHz, CDCl3):

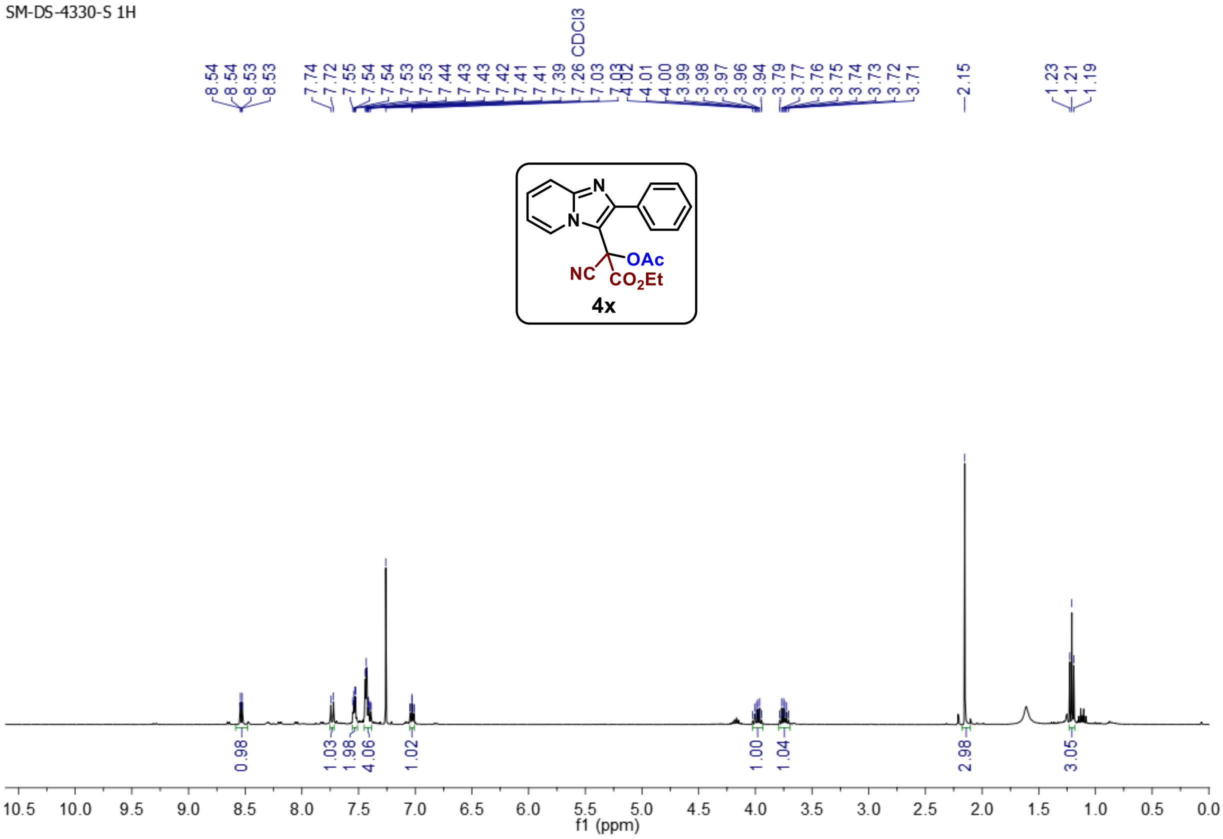

^{13}C{^{1}H} NMR of **4x** (101 MHz, CDCl3):

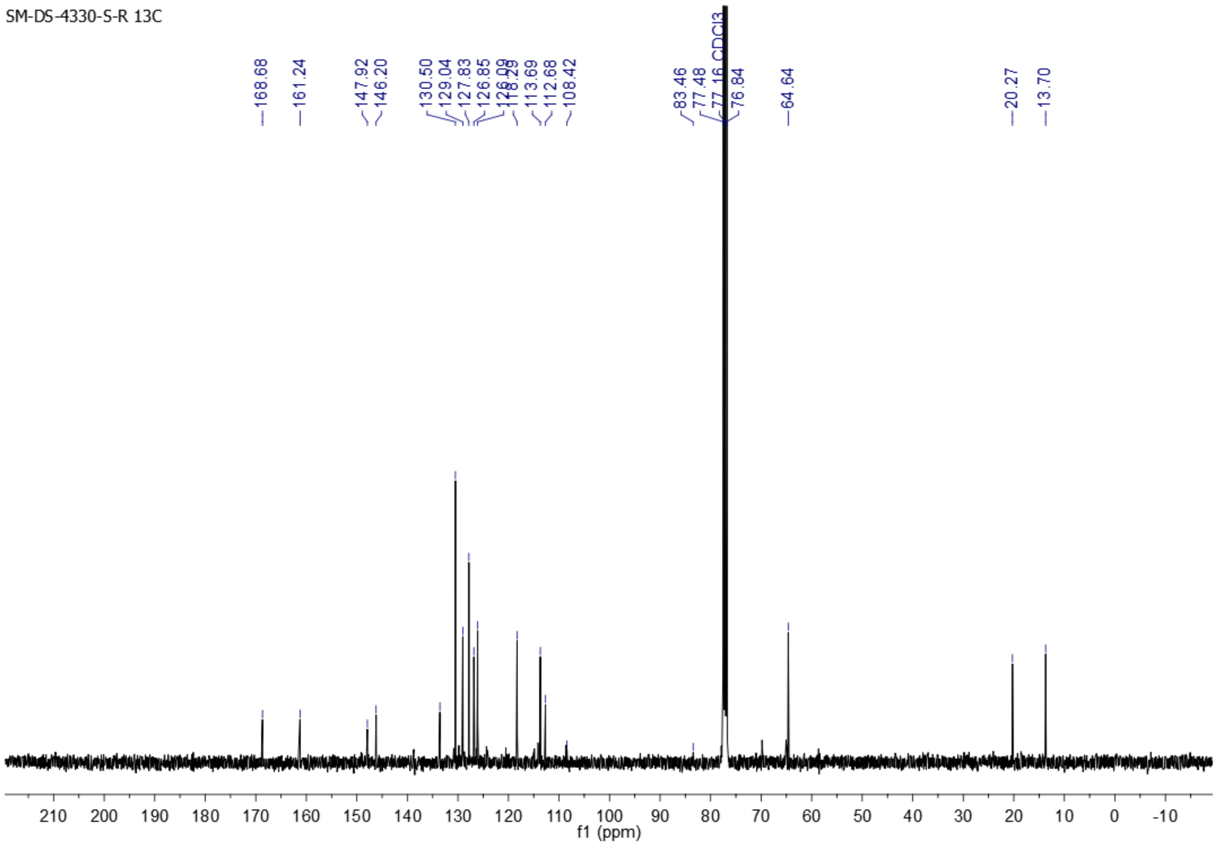

¹H NMR of **4y** (400 MHz, $CDCl_3$):

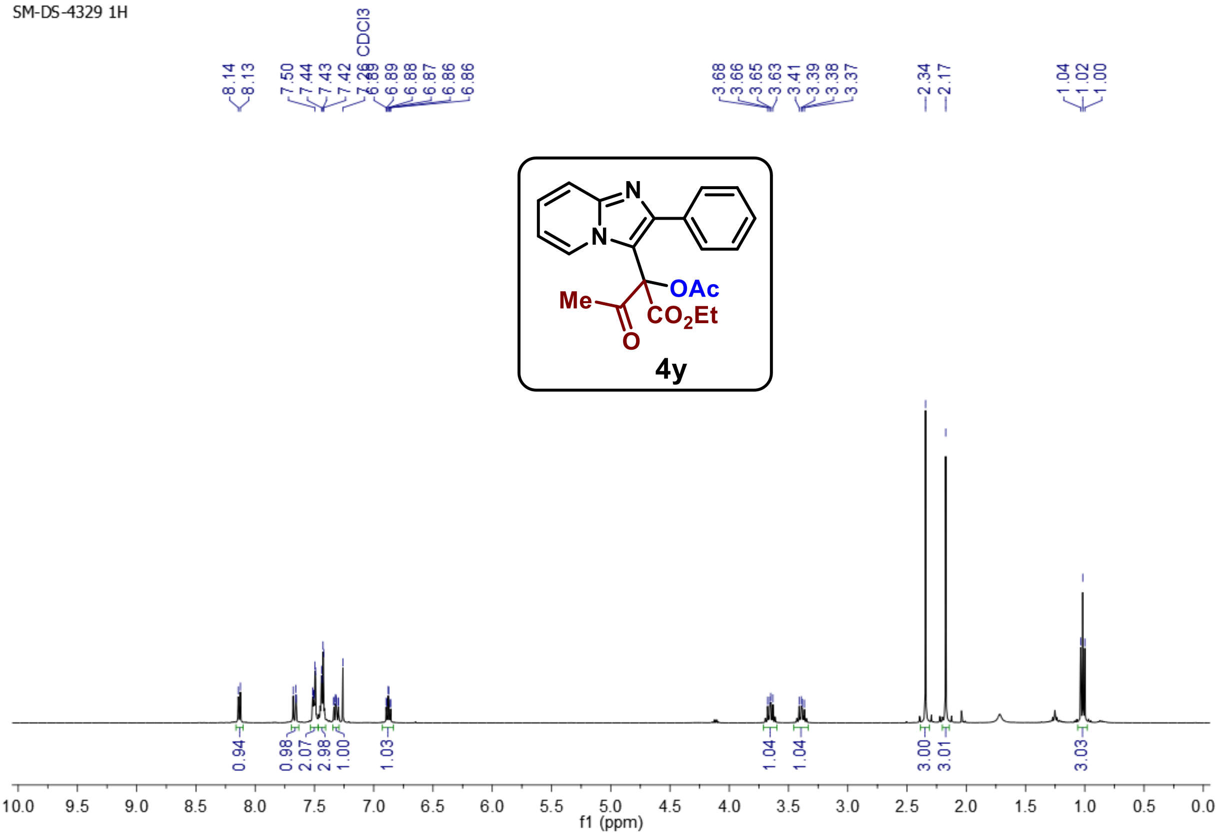

$^{13}C\{^1H\}$ NMR of **4y** (101 MHz, $CDCl_3$):

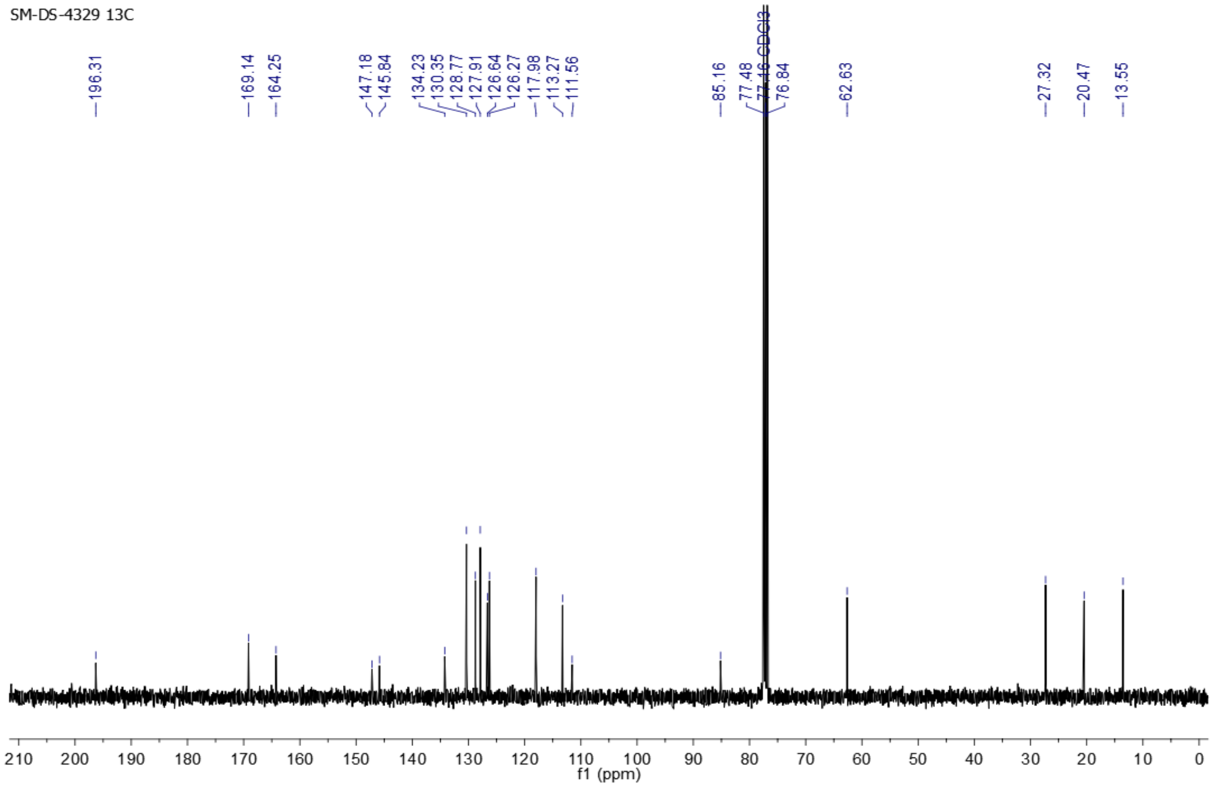

^{1}H NMR of **5** (400 MHz, $CDCl_3$):

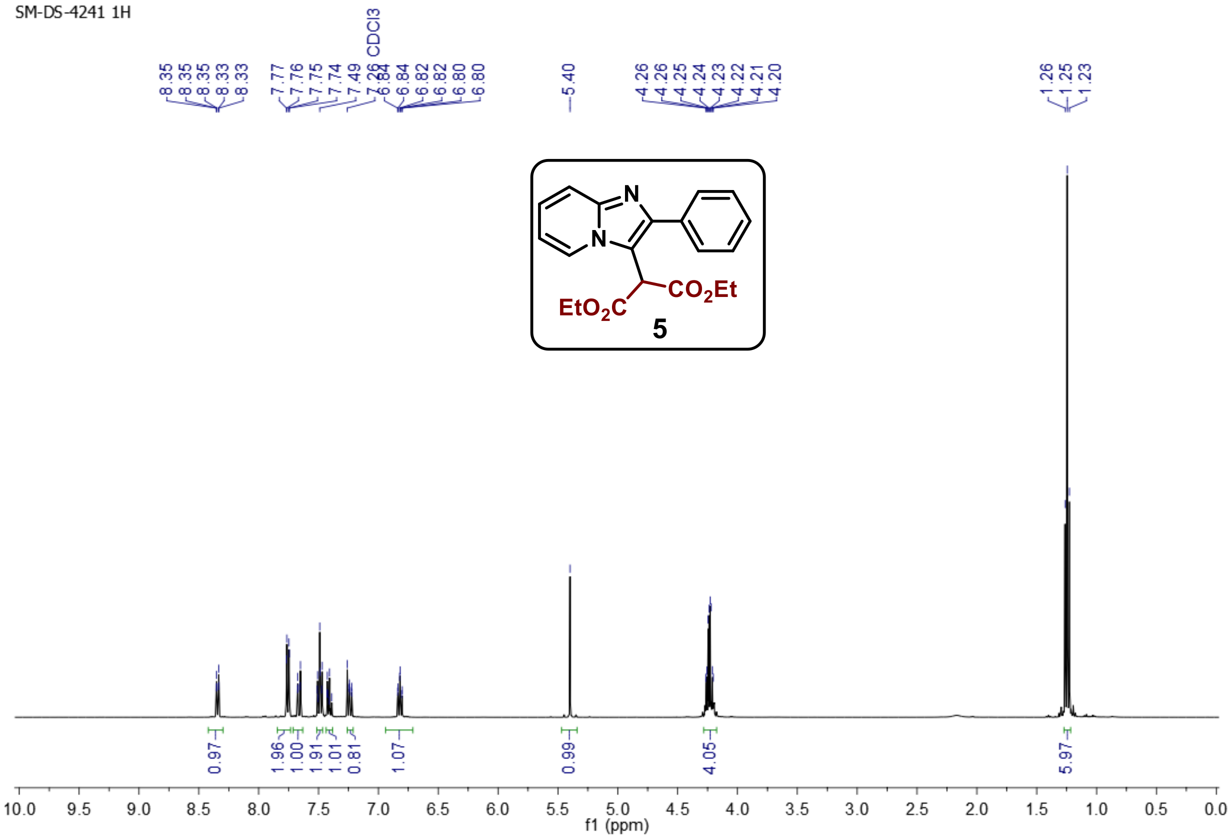

$^{13}C\{^{1}H\}$ NMR of **5** (101 MHz, $CDCl_3$):

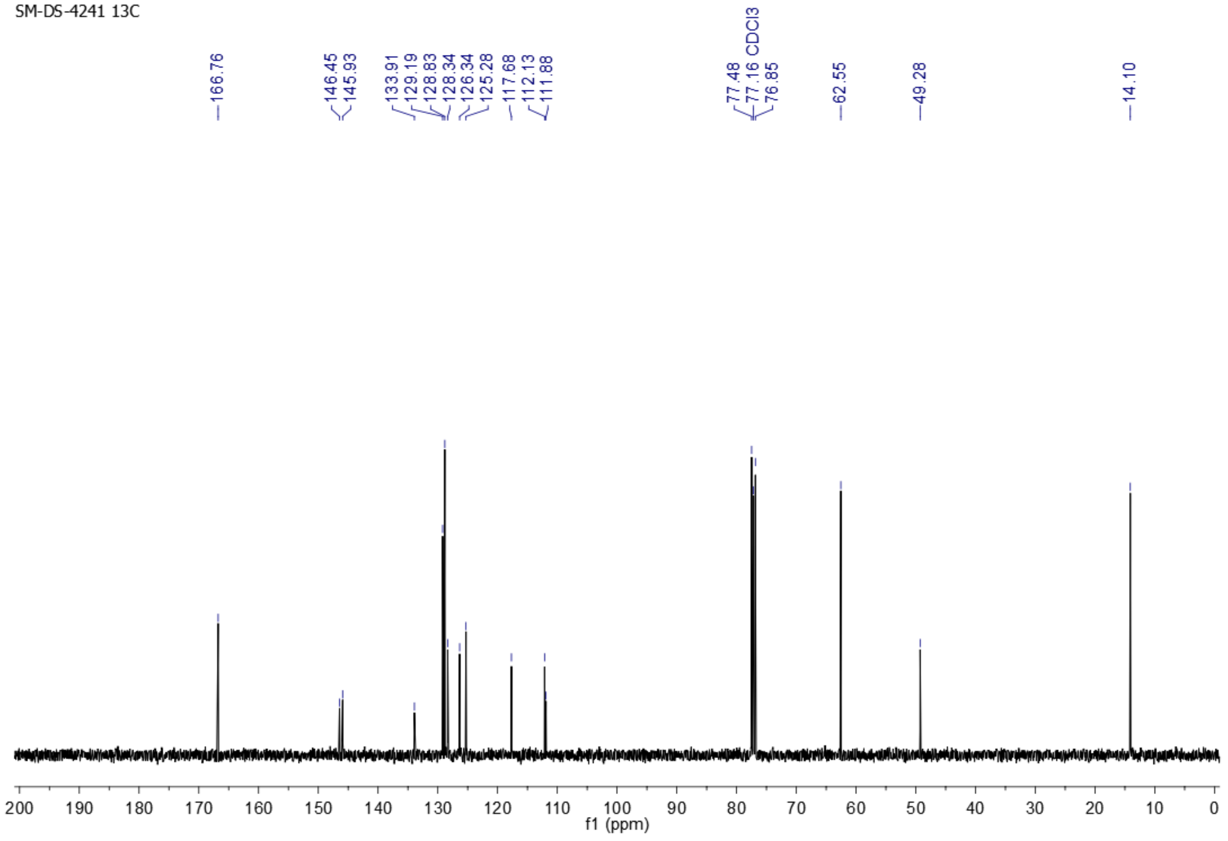

^{1}H NMR of **6** (400 MHz, $CDCl_3$):

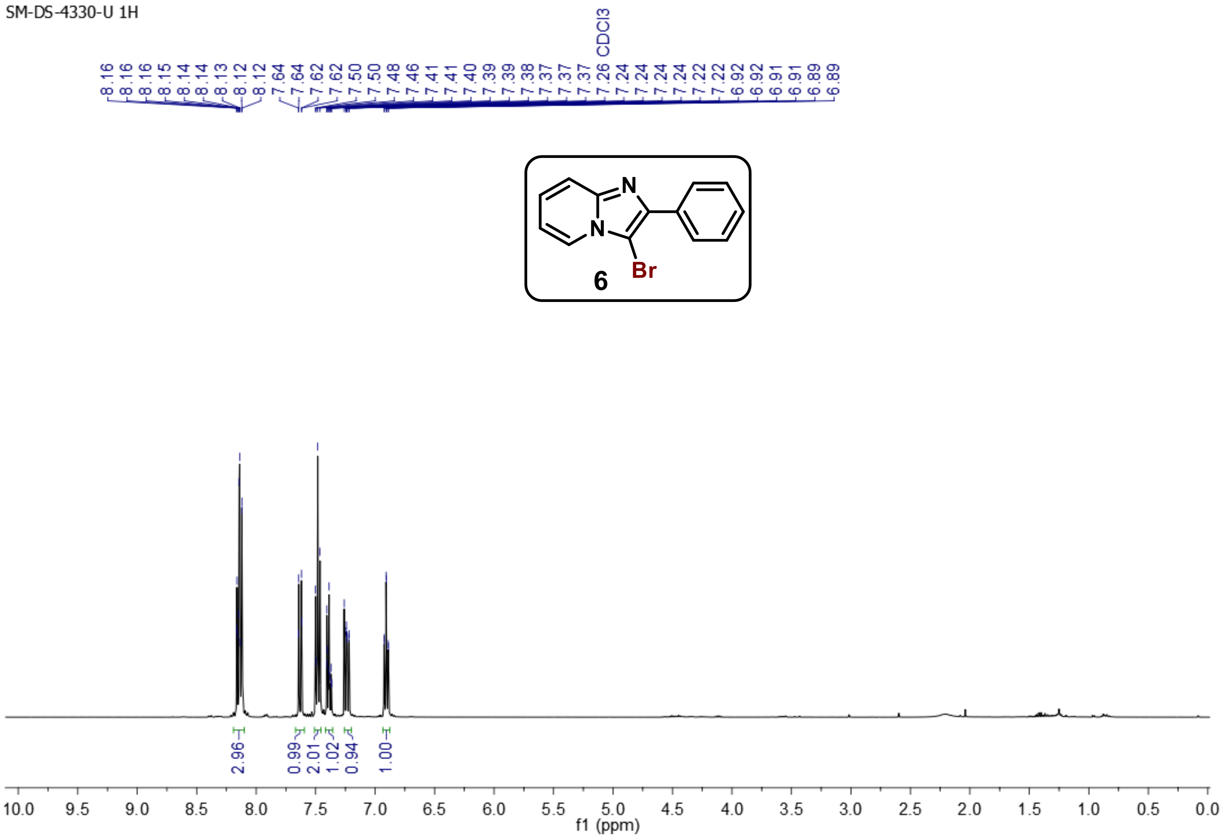

^{13}C{^{1}H} NMR of **6** (101 MHz, $CDCl_3$):

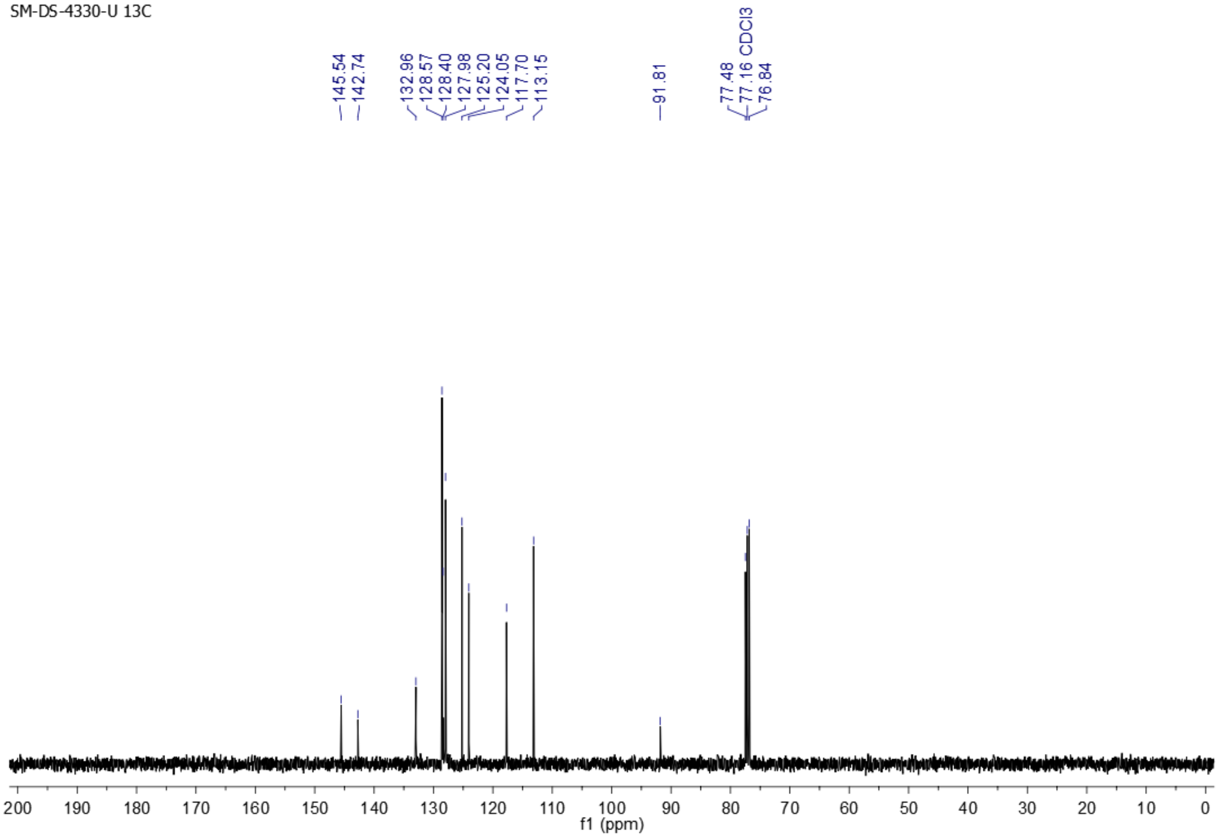

^{1}H NMR of **9** (400 MHz, $CDCl_3$):

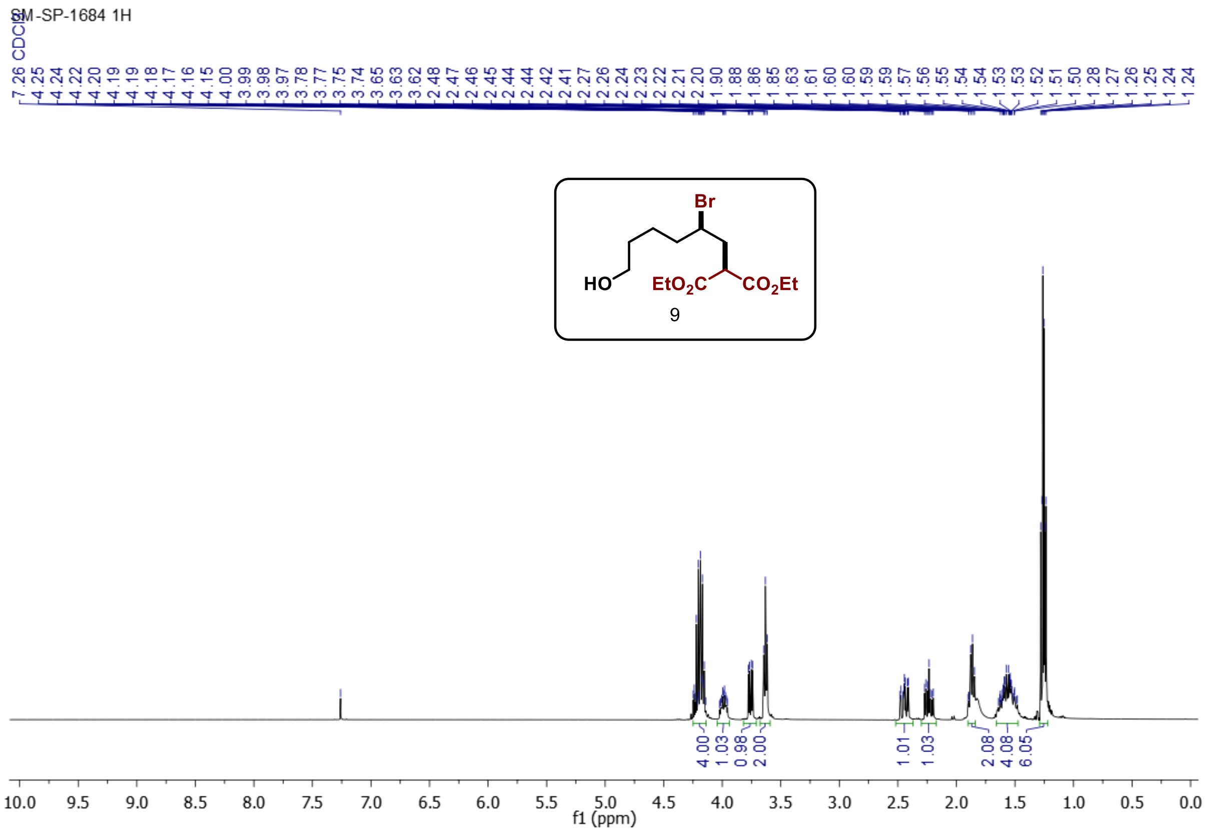

^{13}C{^{1}H} NMR of **9** (101 MHz, $CDCl_3$):

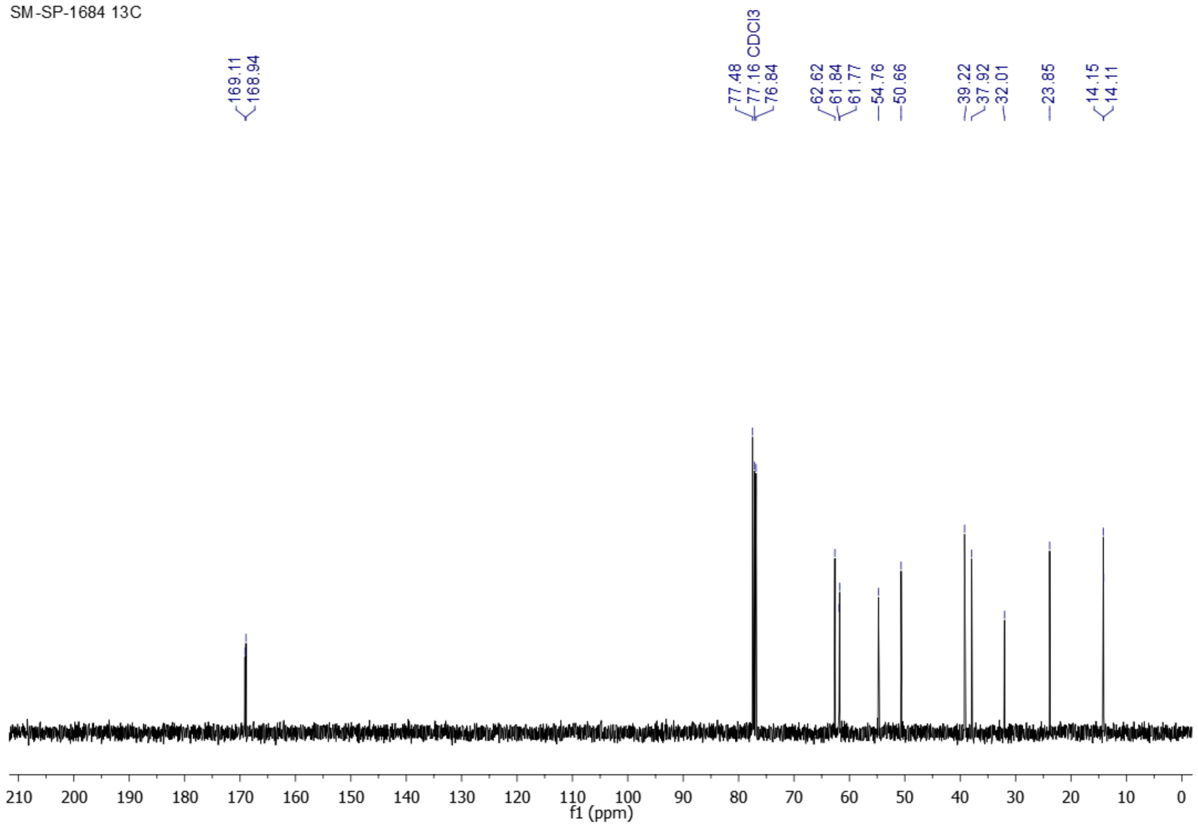

^{1}H NMR of **10** (400 MHz, $CDCl_3$):

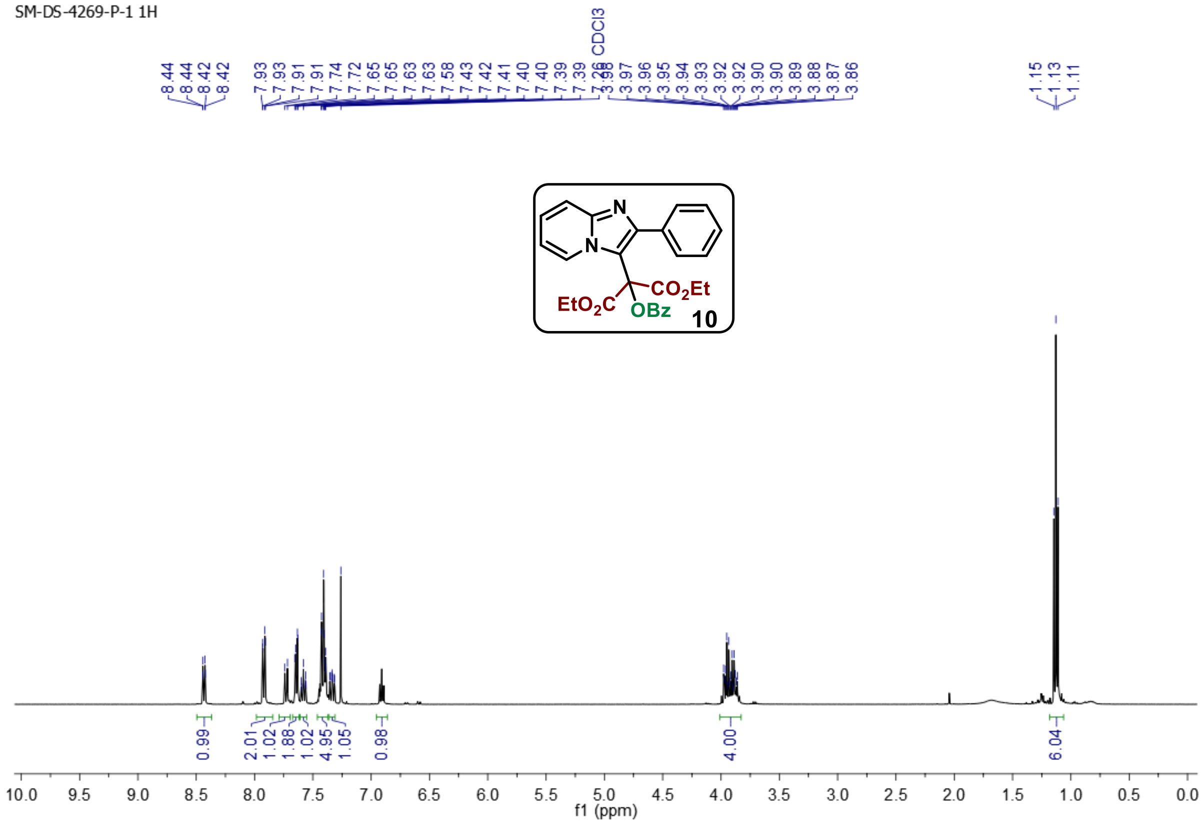

$^{13}C\{^{1}H\}$ NMR of **10** (101 MHz, $CDCl_3$):

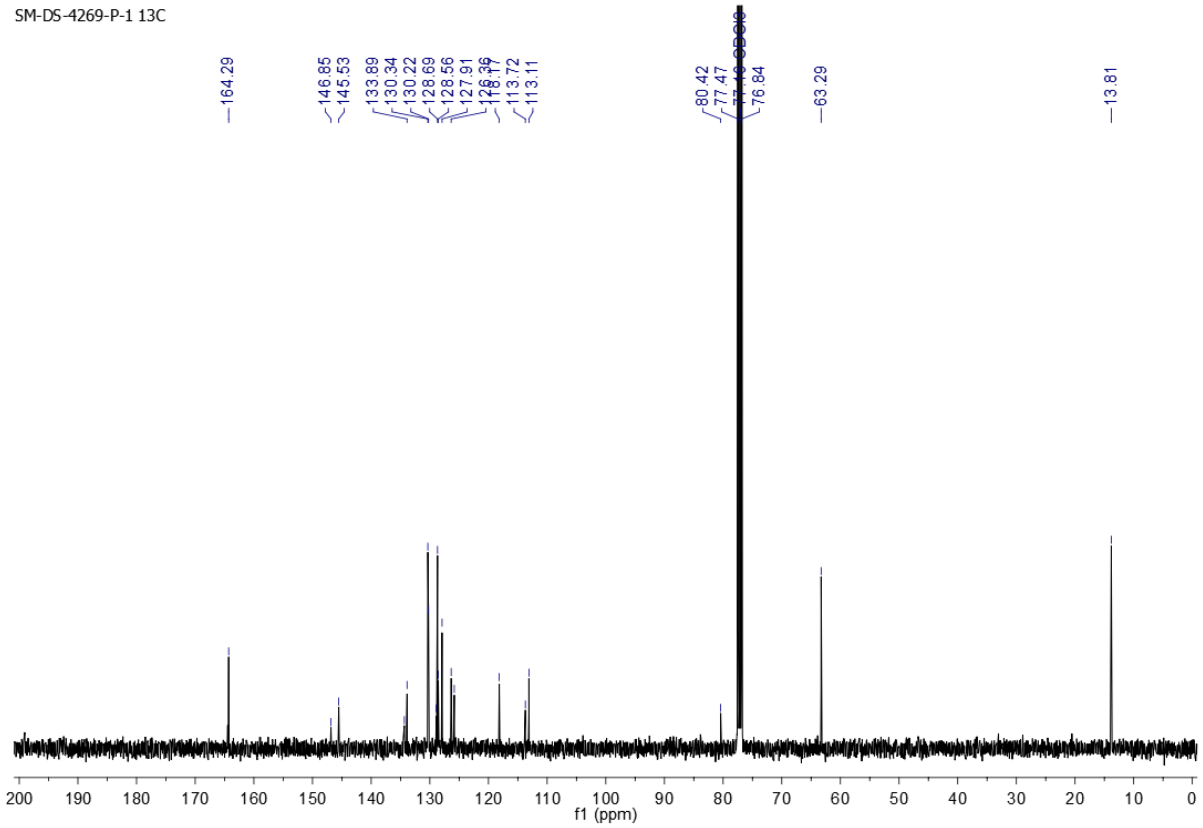

^{1}H NMR of *N*-(pyridin-2-yl)benzamide (400 MHz, $CDCl_3$):

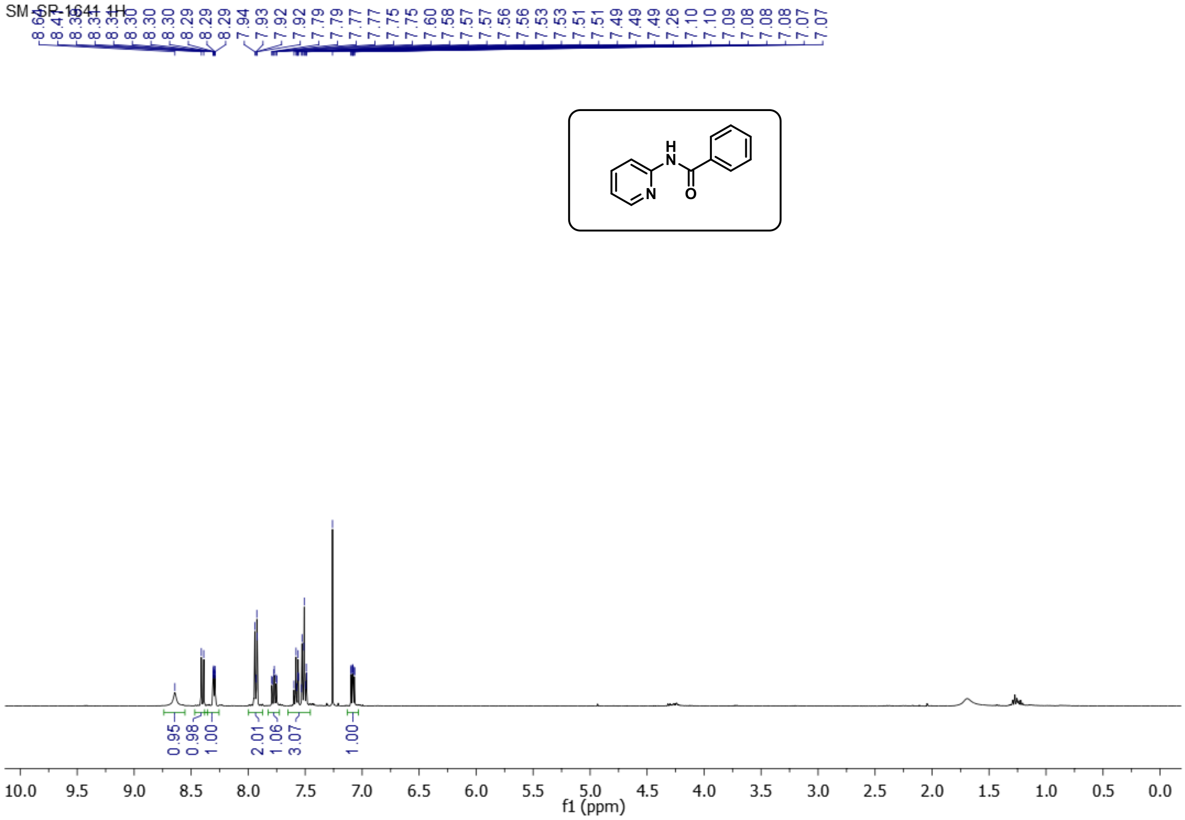

^{13}C{^{1}H} NMR of *N*-(pyridin-2-yl)benzamide (101 MHz, $CDCl_3$):

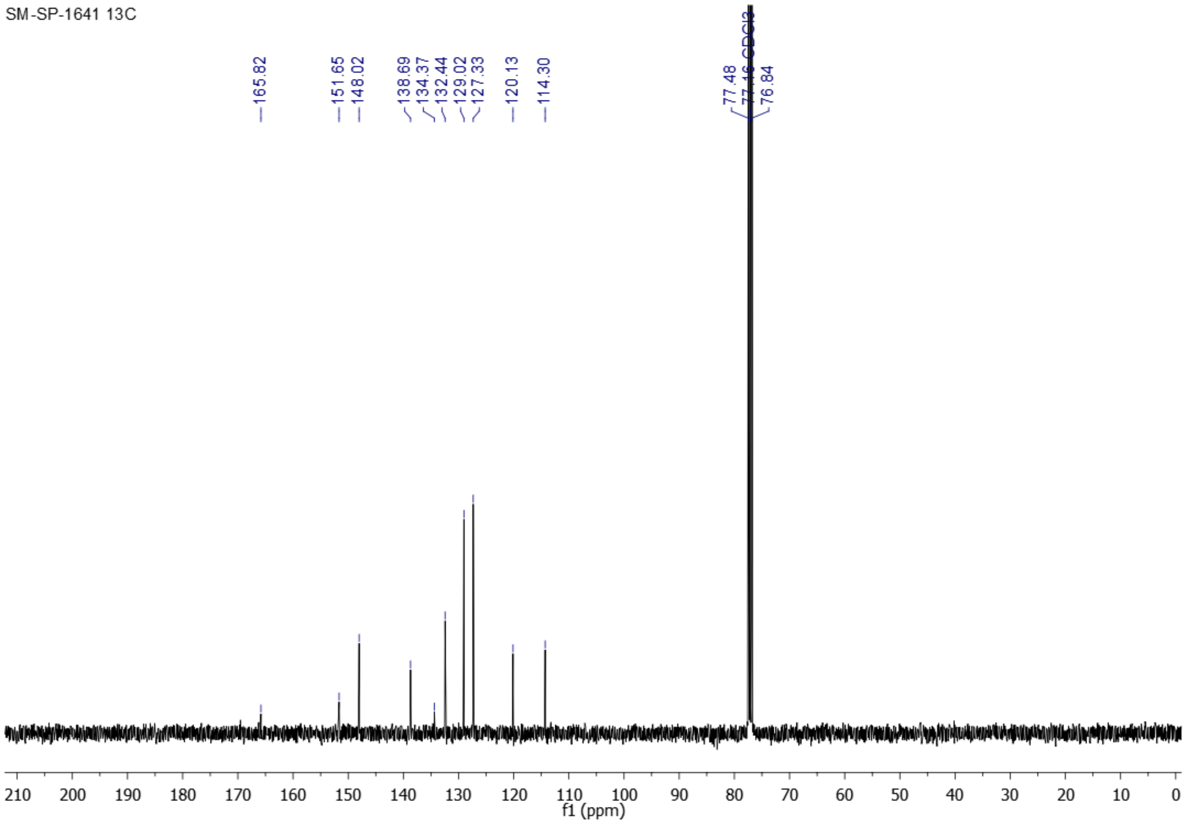

Supplement: File 1 — Experimental section and characterization of synthesized compounds. [file Beilstein_J_Org_Chem-19-666-s001.pdf]
